# Supplementary material for: Flavin-dependent halogenases catalyze enantioselective olefin halocyclization
Source: Nat Commun. 2021 Jun 1;12:3268. doi: 10.1038/s41467-021-23503-3 (PMC8169660; doi:10.1038/s41467-021-23503-3)
Supplement: Supplementary file 1 — Supplementary Information [file 41467_2021_23503_MOESM1_ESM.pdf]

Supplementary Information for

Flavin-Dependent Halogenases Catalyze Enantioselective Olefin Halocyclization

Dibyendu Mondal[1], Brian F. Fisher[1,†], Yuhua Jiang[1], Jared C. Lewis[1,*]

Table of Contents

I. Materials and Instruments 3

A) Materials 3

B) Instruments 3

C) Software 4

II. FDH Expression 4

A) Cloning 4

Introducing K79A into 4V+S 4

B) Protein Expression 4

III. Bioconversion Conditions and Analysis 6

A) High throughput screening 6

Reaction Setup 6

B) Medium-Throughput Screening 7

Reaction Setup 7

C) Preparative Bioconversions for Reported Yields and Selectivities 7

Manuscript Figure 3 Procedure 7

Large Scale Reaction setup 8

IV. Computational Procedures 9

V. Supplementary Figures 12

A) Supplementary Figure 1: Screening of substrate 1. 12

B) Supplementary Figure 2: Enantioselectivity of variant 3-LR. 13

C) Supplementary Figure 2: Activity data for select substrates (1, 2, 4, and 5) using a panel of purified FDHs. 17

D) Supplementary Figure 4: Analysis of the trisubstituted compound. 18

E) Supplementary Figure 5: Chlorolactonization activity of FDH variants. 19

F) Supplementary Figure 6: Examination of chlorolactonization by FDH variants in the 4V+S lineage. Only 4V+S possesses chlorolactonization activity. 19

G) Supplementary Figure 7: Additional docking poses 20

H) Supplementary Figure 8: LC analysis of the *p*-fluoro compound 3.[3] 21

I) Supplementary Figure 9: Steady state kinetics for 4V+S bromolactonization. 21

J) Supplementary Figure 10: Comparison time courses for bromolactonization of 1 by 4V+S variants. 22

K) Supplementary Figure 11: Comparison of bromolactonization by 4V+S, RebH, and their K79A mutants. 23

L) Supplementary Figure 12: Comparison of chlorolactonization by 4V+S, RebH, and their K79A mutants. 24

M) Supplementary Figure 13: RebH-K79A can brominate electron rich substrates. 26

N) Supplementary Figure 14: Improved selectivity for bromolactonization in the presence of glutathione. 26

O) Supplementary Figure 15: Time course experiment for bromolactonization in the presence of a previously reported 4V+S substrate (manuscript reference 29). 27
VI. Supplementary Synthetic Procedures 27
A) General synthetic procedures of Substrates 1,2,3,4 and 6.[4,3] 27
B) General synthetic procedure for the Wittig reaction.[4,3] 28
C) General synthetic procedure for Synthesis of Substrate 8.[5] 30
D) General synthetic procedure for Synthesis of Substrate 7:[6] 31
E) General synthetic procedure for synthesis of racemic standards 32
VII. Calibration Curves 35
VIII. Chiral HPLC Analysis 40
IX. NMR Spectra 45
X. Supplementary References 66

I. Materials and Instruments

A) Materials

Skirted 96-well PCR plates (product number 82006-704) were purchased from VWR International (Radnor, PA). Eppendorf unskirted 96-well PCR plates (product number 951020362) were purchased from Fisher Scientific. Greiner Bio-One polypropylene 96-well V-bottom plates (product number 651201) were purchased from Fisher Scientific. Agilent 0.2 μm PVDF 96-well filter plates (product number 203980-100) were purchased from Agilent. Dialysis tubing (32 mm width; MWCO 6,000-8,000) was purchased from Fisher Scientific.

NAD, FAD, and antibiotics were purchased from Chem-Impex International Inc. (Wood Dale, IL). Antibiotics were prepared as 1000x stock solutions: 1000x chloramphenicol was prepared at 25 mg/mL in EtOH, and 1000x kanamycin was prepared at 50 mg/mL. Substrates were purchased from Sigma-Aldrich,Chem-Impex, AK Scientific, Enamine and Santa Cruz Biotechnologies.

GDH-105 (hereafter, GDH; 50 U/mg) was obtained from Codexis, Inc. (Redwood City, CA). Catalase from bovine liver was obtained from Millipore Sigma (2,000-5,000 U/mg; stock solutions were prepared assuming 2,000 U/mg; product number C9322). Luria broth (LB) and Terrific broth (TB) media were purchased from Research Products International (Mt. Prospect, IL). Qiagen Miniprep Kits were purchased from QIAGEN Inc. (Valencia, CA) and used according to the manufacturer's instructions. Protein ladder (Blue Pre-stained Protein Standard, Broad Range (11-190 kDa); product number P7706) was purchased from New England Biolabs (Ipswitch, MA).

Stock solutions of 10 mM NAD and 10 mM FAD were prepared in 25 mM HEPES pH 7.4 buffer (reaction buffer) and stored at -20 °C until use. Stock solutions of 1.5 M NaCl, 1.5 M NaBr, and 1 M glucose were prepared in reaction buffer and stored at 4 °C until use. Stock solutions of substrate were prepared at 100 mM in DMSO; for the high-throughput screen, substrate stocks were then diluted to 1 mM in reaction buffer, manually arrayed into 96-well plates as described later. RebF was expressed in *E. coli* BL21(DE3) as an MBP fusion from pLIC-MBP and stored at 140 μM in reaction buffer with 10% glycerol at -78 °C. GDH was prepared as 180 U/mL stock solution in reaction buffer immediately before reaction setup. For high-throughput screening, stock solutions of FDH were stored in reaction buffer with 10% glycerol and arrayed in 96-well unskirted PCR plates as described later. Catalase stock solutions were prepared at 3500 U/mL in reaction buffer immediately before reaction setup.

B) Instruments

Thermal plate sealing with aluminum foil was performed using a Packard MicroMate 496 manual plate sealer or by pressing an oven-heated (≈200 °C) aluminum block onto the plate with foil atop it. Measurement of DNA/protein concentration was performed using a Tecan Infinite 200 PRO plate reader on a Tecan NanoQuant plate. High-throughput LC-ESI-MS analysis was performed using an Agilent system equipped with a 1290 Infinity II Multisampler, a 1260 Infinity binary pump, and a 6130 single quadrupole mass spectrometer with an ESI/APPI multimode source. Analytical-scale reactions were analyzed by LC-MS using either (1) an Agilent system equipped with a 1290 Infinity II Multisampler (dual-needle configuration), a 1290 Infinity II high-speed pump, a 1260 Infinity II diode array detector, and a 6135X single quadrupole mass spectrometer with an Agilent Jet Stream ESI source or (2) an Agilent 1290 UHPLC with an Agilent Eclipse Plus C18 column. CHIRAL column information. Chiralpak IH-U (3mm x 50 mm), Chiralpak IG-U (3mm x 50 mm), Chiralpak AD-H (4.6mm x 250 mm). Preparative-scale bioconversions were purified using either: An Agilent 1100 HPLC equipped with a Supelco Discovery C18 semipreparative column (25 cm x 10 mm, 5 μm particle size) and an Agilent 1260 Infinity

II fraction collector using 0.1% formic acid in H_2O as the A solvent and 0.1% formic acid in acetonitrile as the B solvent.

C) **Software**

HPLC traces were processes using Agilent Chemstation Rev. C.01.08(224). NMR spectra were processed using MestReNova 11.0. Plots were generated using GraphPad Prism 7.0 and Microsoft Excel (version 2013). Geometry optimization was conducted using Spartan 18, Wavefunction Inc., Irvine CA. Docking simulations were conducted using Autodock Vina as implemented in UCSF Chimera candidate version 1.13.1.

II. FDH Expression

A) **Cloning**

Introducing K79A into 4V+S

Forward and reverse primers used were 5’ -GAGTGCAACGCGAGCTACGCGGTCGCCATC - 3’ and 5’ – GTAGCTCGCGTTGCACTCCCGCACCCACTC – 3’, respectively. The PCR conditions were adapted from PrimeSTAR Max DNA Polymerase (Takara bioscience, Cat. #R045A) protocol.

Approximately 200 ng parent template, 0.3 µM forward primer, 0.3 µM reverse primer, molecular biology grade water and 1X Primestar Max. PCR was performed in a volume of 50 µL with the following procedure: 98 °C 10 s, (98 °C 10 s, 62 °C 15 s, 72 °C 220 s) for 16 cycles, 72 °C 5 min. The resulting 4V+S+K79A plasmid was cleaned up and eluted with 35 µL hot water. This product was then subjected to DPN1(1 µL) digestion in cut smart buffer at 37 °C for 1 hour. The plasmid was then gel purified and eluted with 20 µL hot water.

Transformation

Electro-competent BL21(DE3) *E. coli* cells were prepared using a protocol given on the NEB site (doi.org/10.17504/protocols.io.crtv6m). Electro-competent cells were transformed with pET28b containing FDH insert. Aliquots of competent cells were transferred to 5 mL polypropylene culture tubes on ice, and approximately 10 ng of plasmid was added to each tube. Competent cells were incubated with plasmid on ice for 1 min, and the cells were transformed via electroporation and immediately recovered using 750 µL SOC medium. The culture tubes were incubated at 37 °C with shaking at 250 rpm for 1 hr to recover. After recovery, 100 µL cells were added to agar plates (with 50 µg/mL kanamycin) and spread using 3 mm glass beads. After drying, the agar plates were transferred to a 37 °C incubator and grown overnight. Single colonies were picked and grown in 5 mL TB with kanamycin overnight. On the next day 500 uL of culture was transferred to a sterile 2ml Eppendorf vial and to it added 500 µL autoclaved 50% glycerol, and this glycerol stock vial was kept at -80 °C.

B) **Protein Expression**

Small-scale FDH expression

96-well plates containing glycerol stocks of BL21(DE3) + pGro7 FDH[1] expression cultures were stamped into autoclaved 2 mL 96-well deep well plates containing 1 mL LB with kanamycin and chloramphenicol. These inoculated plates were sealed with an AeraSeal adhesive film and incubated overnight at 37 °C, 220 rpm overnight. The overnight cultures (20 µL) were used to inoculate 2 mL antibiotic-containing TB media in 96-well deep well plates. Inoculated expression cultures were sealed with an AeraSeal adhesive

film and incubated at 37 °C, 235 rpm until OD_{600} ≈0.6-0.8, at which point the incubator was cooled to 15 °C. After the cultures were cooled sufficiently (≈15 min) protein expression was induced with 2 mg/mL L-arabinose and 10 µM IPTG and kept shaking at 30 °C. Protein expression proceeded for 20 hr, at which point the cells were pelleted by centrifugation at 2400× g, 4 °C for 15 min. The supernatant was discarded, and the cell pellets were resuspended in 100 µl lysis buffer (0.75 mg/mL lysozyme, 25 mM HEPES, pH 7.4). Cells were incubated in lysis buffer at 37 °C, 250 rpm for 30 min. After lysis, the suspensions were frozen by immersing the 96-well plates in liquid N_2, thawed at room temperature for 15 min, then transferred to a warm water bath. Once thawing was complete, 20 µL DNAse buffer (1 mg/mL DNAse, 25 mM HEPES, pH 7.4) was added, and the 96-well plates were incubated at 37 °C, 250 rpm for 15 min. The insoluble fraction of the cell lysate was pelleted by centrifugation at 2400× g, 4 °C for 15 min. The supernatant was isolated, and the insoluble fraction of the cell lysate was discarded.

Medium-scale FDH expression

14 mL culture tubes containing 5 mL LB with kanamycin and chloramphenicol were inoculated with a glycerol stock of BL21(DE3) + pGro7 + pET28b(FDH) and incubated overnight at 37 °C, 250 rpm. The next day, 100 mL TB with antibiotics in a 500 mL Erlenmeyer was inoculated with 1 mL of the overnight cultures. The inoculated expression cultures were incubated at 37 °C, 250 rpm until OD_{600} ≈0.6-0.8, at which point the incubator was cooled to 30 °C. Once the liquid cultures were cool (about 15 min), protein expression was induced with 2 mg/mL L-arabinose and 100 µM IPTG, and the expression cultures were incubated for 20 hr. Once protein expression was complete, the expression cultures were transferred to 50 mL centrifuge tubes and centrifuged at 2400× g, 4 °C for 15 min. The supernatant media was discarded, and the cell pellets were resuspended in 10 mL 25 mM HEPES, pH 7.4. Cell suspensions were sonicated on ice using a QSonica S-4000 with a 0.5” horn at 40W using 1 min on/1 min off cycles for 5 min total cycle time. Cell lysates were clarified by centrifuging at 24,000 × g in a high-speed fixed-angle rotor for 40 min at 4 °C. The soluble fraction of the lysate was decanted into a new 50 mL centrifuge tube, then transferred to 10 mL polypropylene frit-bottomed spin columns capped at the bottom and containing 500 µL Ni-NTA resin pre-equilibrated with equilibration buffer (20 mM phosphate, 300 mM NaCl, 10 mM imidazole, pH 7.4). The columns were capped on top, inverted a few times to mix evenly, and transferred back to the centrifuge tubes which were then capped. Protein was bound to resin by gentle mechanical inversion of these centrifuge tubes for 1 hr at 4 °C. After the binding step, the Ni-NTA suspensions were transferred back to uncapped spin columns and allowed to drain by gravity into a waste basin. 10 mL of wash buffer (20 mM phosphate, 300 mM NaCl, 25 mM imidazole, pH 7.4) was added to the columns, which were allowed to drain by gravity into a waste basin. The spin columns were nested within new 50 mL centrifuge tubes, and 10 mL elution buffer (20 mM phosphate, 300 mM NaCl, 250 mM imidazole, pH 7.4) was added and allowed to drain into the centrifuge tubes by gravity. The eluted protein solutions were transferred to 4 mL Amicon Ultra 10K MWCO spin filters and concentrated by centrifugation at 4000xG for ≈15 min at 4 °C. Protein solution was diluted with 25 mM HEPES, pH 7.4, and centrifuged again. Buffer exchange in this manner was performed 3-5 times, after which glycerol was added for a final concentration of 10% v/v. Protein solutions were centrifuged at 4000 xG at 4 °C for 3 min prior to measuring concentration using absorbance at 280 nm using a Tecan NanoQuant plate with protein extinction coefficients.

III. Bioconversion Conditions and Analysis

A) **High throughput screening**

Reaction Setup

**NaX**	5 eq
**glucose**	20 mM
**NAD**	10 μM
**FAD**	10 μM
**GDH**	9 U/mL
**Catalase**	35 U/mL
**MBP-RebF**	2.5 μM
**FDH**	50 μM
**substrate**	1000 μM
**Rxn Volume**	70 μL

Small molecule mixture: A mixture of the small molecule components of the FDH bioconversions (NaBr, Appropriate substrate, glucose, NAD, and FAD) was prepared in reaction buffer. This small molecule mix was manually arrayed into 96-well plate.

Enzyme mixture: A mixture of the cofactor regeneration components of the FDH bioconversions (GDH, MBP-RebF and FDH) was prepared in reaction buffer. The cofactor regeneration mix was manually arrayed into 96-well plate containing the small molecule mixture.

After all reactions had been prepared, the 96-well plates were sealed with adhesive film and shaken on a plate shaker at 650 rpm for 18 hr.

Reaction workup

After 18 h of shaking at rt the 96-well plate was centrifuged at 2400× g, 4 °C for 5 min. Then the reactions were quenched, and proteins precipitated by adding 70 μL MeOH premixed with appropriate internal standards (see section VII) to each well of the 96-well reaction plate manually. Reaction plates were then centrifuged at 2400× g, 4 °C for 5 min to pellet precipitated protein. Using a multichannel pipette, 80 μL of supernatant from the reaction plates were transferred to new 96-well filter plate retaining the well layout of the previous plate (this process removed solids sufficiently for LC analysis; system pressure of the LC-MS did not increase significantly after >10,000 injections of these reactions). The filter plate with a new 96 V-bottom deep well plates were then centrifuged at 2400× g, 4 °C for 5 min. The collected filtrate were then sealed with aluminum foil and analyzed using LCMS.

Analysis was performed using an Agilent 1100 HPLC equipped with an Agilent Eclipse Plus C18 column (3.5 μm particle size; 4.6 x 150 mm), eluting with a gradient of 30-70% B over 4.5 min. Product (P) assay yields (AY) were determined relative to internal standard (IS) with appropriate response factors (RF) using the formula $AY = RF \cdot (integral_P/integral_{IS}) + C$. RF values are provided in section VII. Authentic racemic products were used for RF determination were prepared as described in section VI, and HPLC traces for the racemic products (needed to identify product enantiomers in bioconversions) and bioconversions are provided in section VIII.

B) **Medium-Throughput Screening**

Reaction Setup

**NaX**	5 eq
**glucose**	20 mM
**NAD**	100 μM
**FAD**	100 μM
**GDH**	9 U/mL
**Catalase**	35 U/mL
**MBP-RebF**	2.5 μM
**FDH**	50 μM
**substrate**	1000 μM
**Rxn Volume**	70 μL

Small molecule mixture: A mixture of the small molecule components of the FDH bioconversions (NaBr, Appropriate substrate, glucose, NAD, and FAD) was prepared in reaction buffer. This small molecule mix was manually arrayed into 96-well plate using a multi-channel pipette.

Enzyme mixture: A mixture of the cofactor regeneration components of the FDH bioconversions (GDH, MBP-RebF and FDH) was prepared in reaction buffer. The cofactor regeneration mix was manually arrayed into 96-well plate containing the small molecule mixture using a multi-channel pipette.

After all reactions had been prepared, the 96-well plates were sealed with adhesive film and shaken on a plate shaker at 650 rpm for 18 hr.

Reaction workup

After 18 h of shaking at rt the 96-well plate was centrifuged at 2400× g, 4 °C for 5 min. Then the reactions were quenched, and proteins precipitated by adding 70 μL MeOH premixed with appropriate internal standards to each well of the 96-well reaction plate manually. Reaction plates were then centrifuged at 2400× g, 4 °C for 5 min to pellet precipitated protein. Using a multichannel pipette, 80 μL of supernatant from the reaction plates were transferred to new 96-well filter plate retaining the well layout of the previous plate (this process removed solids sufficiently for LC analysis; system pressure of the LC-MS did not increase significantly after >10,000 injections of these reactions). The filter plate with a new 96 V-bottom deep well plates were then centrifuged at 2400× g, 4 °C for 5 min. The collected filtrate were then sealed with aluminum foil and analyzed using LCMS.

Analysis was performed using an Agilent 1100 HPLC equipped with an Agilent Eclipse Plus C18 column (3.5 μm particle size; 4.6 x 150 mm), eluting with a gradient of 30-70% B over 4.5 min.

C) **Preparative Bioconversions for Reported Yields and Selectivities**

Manuscript Figure 3 Procedure

Analytical-scale bioconversion and LC analysis were conducted using substrate/small molecule/FDH/cofactor regeneration mixtures similar to the high-throughput screen reaction setup. Reactions were allowed to shake at 650 rpm for different interval of time based on the substrate.

**NaBr**	5 eq
**glucose**	20 mM
**NAD**	100 µM
**FAD**	100 µM
**GDH**	9 U/mL
**MBP-RebF**	2.5 µM
**Catalase**	35 U/mL
**FDH**	50 µM
**Substrate**	1 mM
**Glutathione**	1 eq
**Rxn Volume**	70 µL

Reaction workup

Reaction mixtures were transferred to a vial and quenched with equal volume of 100 µM internal standard in MeOH. Precipitated protein was pelleted by centrifugation at 2400× g at 4 °C for 5 min. The supernatant was filtered through a 0.22 micron syringe filter placed atop a V-bottom 96-well plate. The filtered quenched reaction plate was heat-sealed with aluminum foil then kept for analysis.

UV traces (210, 220, or 230 nm) were manually reviewed and integrated. Conversion for each experiment was computed based on the internal standard added. Analysis was performed using an Agilent 1290 UPLC equipped with an Agilent Eclipse Plus C18 column (3.5 µm particle size; 4.6 x 150 mm), eluting with a gradient of 30-70% B over 4.5 min.

Large Scale Reaction setup

Preparative halocyclization was conducted using a substrate/small molecule/FDH/cofactor regeneration mixture similar to the high-throughput screen reaction setup. Reactions were allowed to shake at 140 rpm for 18 hours in a 500 ml conical flask.

**NaBr**	5 eq
**glucose**	20 mM
**NAD**	100 µM
**FAD**	100 µM
**GDH**	9 U/mL
**MBP-RebF**	2.5 µM
**Catalase**	35 U/mL
**4V+S**	80 µM
**substrate**	2 mM (13 mg)
**Glutathione**	1 eq
**Rxn Volume**	32 ml

Reaction workup

The reaction mixture was transferred to a falcon tube. The conical flask was rinsed with water and added to the tube. Protein was pelleted by centrifugation at 24,000 × g at 4 °C for 30 min. The supernatant was transferred into a separatory funnel and extracted with ethyl acetate 5 times. The precipitated protein was then resuspended in methanol, centrifuged at 24,000 × g at 4 °C for 30 min. The organic layers were

combined, dried over sodium sulfate, concentrated, and purified using semi-preparative HPLC. The product was isolated as 14.87 mg (83%) of colorless oil with 95:5 e.r.

The proton NMR spectrum matched that for the authentic racemic material. ^{1}H NMR, ($CDCl_3$, 600 MHz): δ 7.3 (2H), 6.9 (2H), 3.83 (3H), 3.73 (1H), 3.66 (1H), 2.80 (2H), 2.55 (2H). HRMS: Calculated: 284.005, Found, (M-H) 284.0041.

Semiprep UV chromatogram at A230:

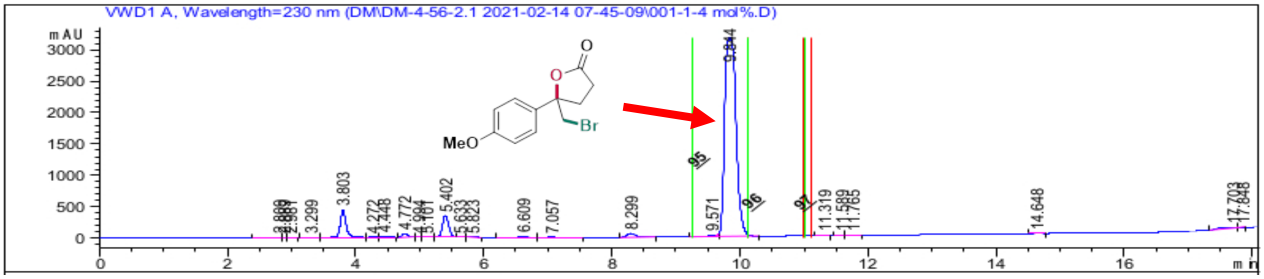

Chiral chromatogram at A230

Racemic

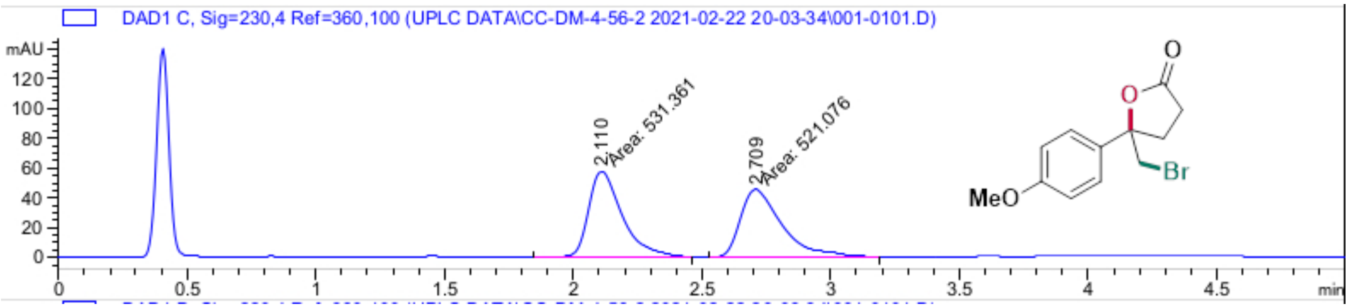

Bioconversion

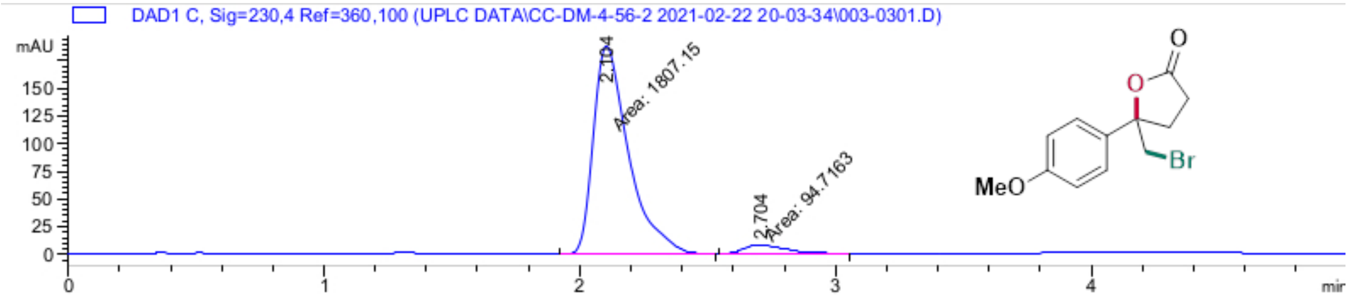

IV. Computational Procedures

A. Geometry Optimization

Geometry optimization of substrate **1** (deprotonated carboxylate) and the corresponding cationic Br^{+} adduct (deprotonated carboxylate) were conducted using Spartan 18, Wavefunction Inc., Irvine CA. The equilibrium geometry at ground state in polar solvent using the appropriate charge state was calculated

using DFT with the B3LYP functional and a 6-31G* basis set. Coordinates of the optimized structures follow:

1-CO_2^-

C	-1.156395	0.096814	-1.001097
C	-1.205606	0.510718	1.743559
C	0.016026	0.547285	-0.372566
C	-2.337762	-0.135527	-0.294339
C	-2.368908	0.076071	1.090054
C	-0.037817	0.734227	1.023833
O	-3.464011	-0.119735	1.881842
C	-4.675880	-0.546967	1.260475
C	1.256595	0.820606	-1.152359
C	1.193516	1.296312	-2.408687
C	2.599393	0.532962	-0.507003
C	2.851158	-0.956725	-0.225678
C	4.263063	-1.296033	0.335680
O	5.122227	-0.372128	0.366094
O	4.423083	-2.493856	0.701287
H	0.250083	1.531350	-2.894898
H	2.096652	1.471604	-2.988382
H	2.698203	1.090123	0.434655
H	3.394967	0.908003	-1.158005
H	-5.412339	-0.619018	2.062469
H	-5.017204	0.179450	0.513208
H	-3.213383	-0.490598	-0.825914
H	-1.144824	-0.097748	-2.069800
H	-1.237165	0.669220	2.817841
H	0.842483	1.077355	1.559189
H	2.106027	-1.352272	0.476410
H	2.714419	-1.533583	-1.152783
H	-4.556601	-1.527912	0.784915

1-Br^+-CO_2^-

C	0.226292	1.300995	-0.982268

C	0.119551	1.001121	1.805469
C	0.283823	-0.013534	-0.412652
C	0.140918	2.426786	-0.199699
C	0.083792	2.290275	1.210373
C	0.224785	-0.116161	1.022372
O	-0.006839	3.306361	2.056118
C	-0.051991	4.663628	1.562946
C	0.400195	-1.168659	-1.204769
C	0.507845	-2.525685	-0.616184
C	-0.919072	-3.065594	-0.255345
C	-0.975462	-3.698905	1.181543
O	-1.956144	-4.461279	1.354978
O	-0.090256	-3.359761	2.009712
H	1.084855	-2.532933	0.310300
H	0.979773	-3.204567	-1.331055
H	-0.120385	5.285580	2.454115
H	0.860880	4.895620	1.008141
H	0.115493	3.405736	-0.661499
H	0.276270	1.433528	-2.056268
H	0.061616	0.932001	2.886613
H	0.231580	-1.095793	1.493528
H	-1.663849	-2.260114	-0.264107
H	-1.249821	-3.800940	-0.993679
H	-0.932969	4.807295	0.931952
C	0.351434	-1.109344	-2.698788
Br	2.227621	-1.092345	-3.375345
H	-0.121655	-0.228738	-3.121995
H	-0.088283	-2.014574	-3.114508

B. **Docking Simulations**

Docking simulations were conducted using Autodock Vina as implemented in UCSF Chimera. Geometry optimized ligands were first imported into Chimera and prepped with Dock Prep using the default program settings, other than the appropriate charge. The crystal structure for RebH variant 3LSR (PDB ID 4LU6) was prepped with Dock Prep using the default program settings. Mutants of this enzyme were generated in Chimera using the Rotamers application; the highest probability rotamer was used. The appropriate prepped structures were loaded into Autodock Vina, and the receptor search volume was set to encompass the entire FDH active site. Selected models were exported as .pdb files for visualization and graphic preparation in Pymol (see Supplementary Figures).

V. Supplementary Figures

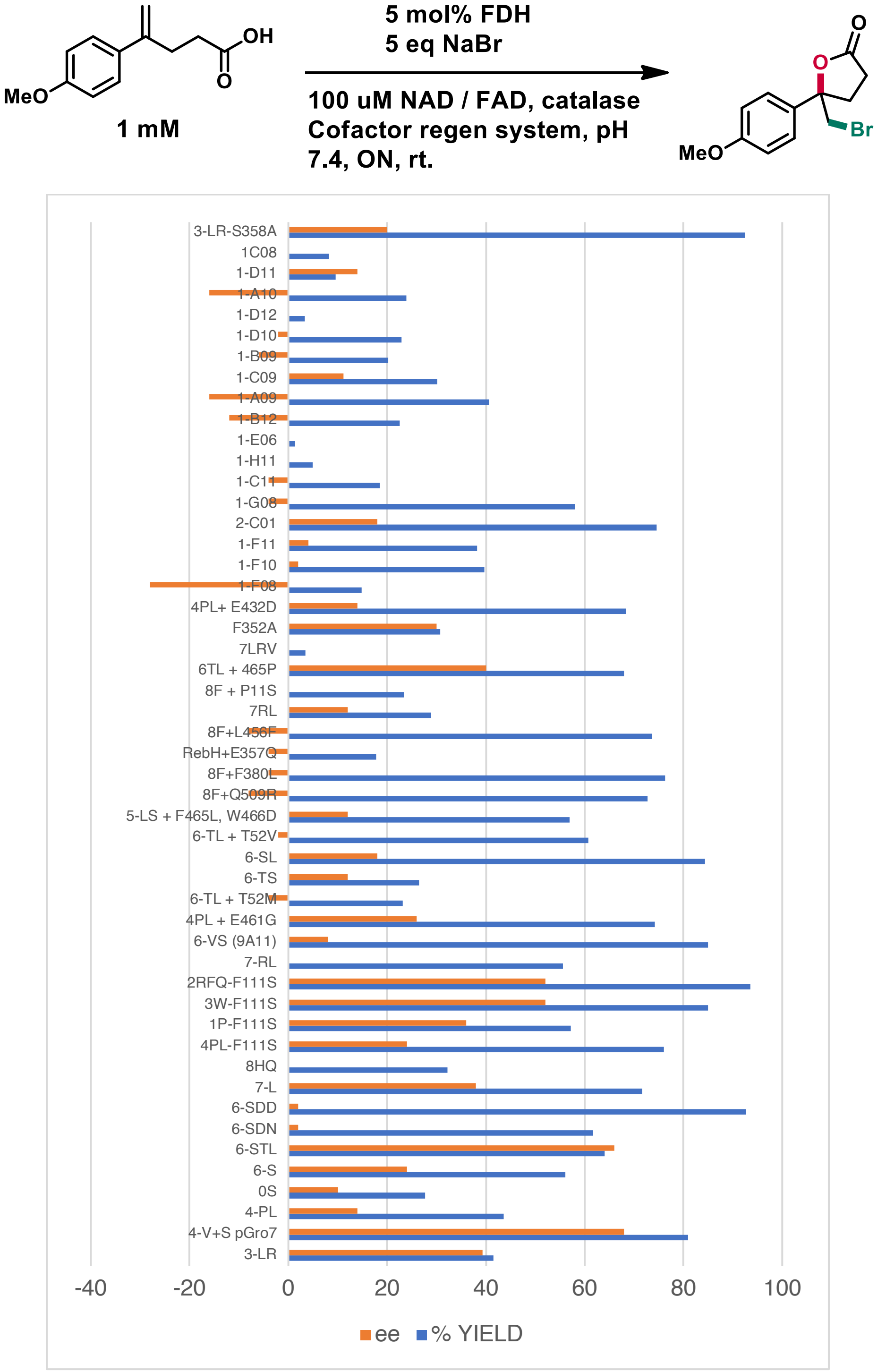

A) **Supplementary Figure 1:** Screening of substrate 1.

50 variants were purified and screened that provided significant yields of bromolactonization product **1a**.

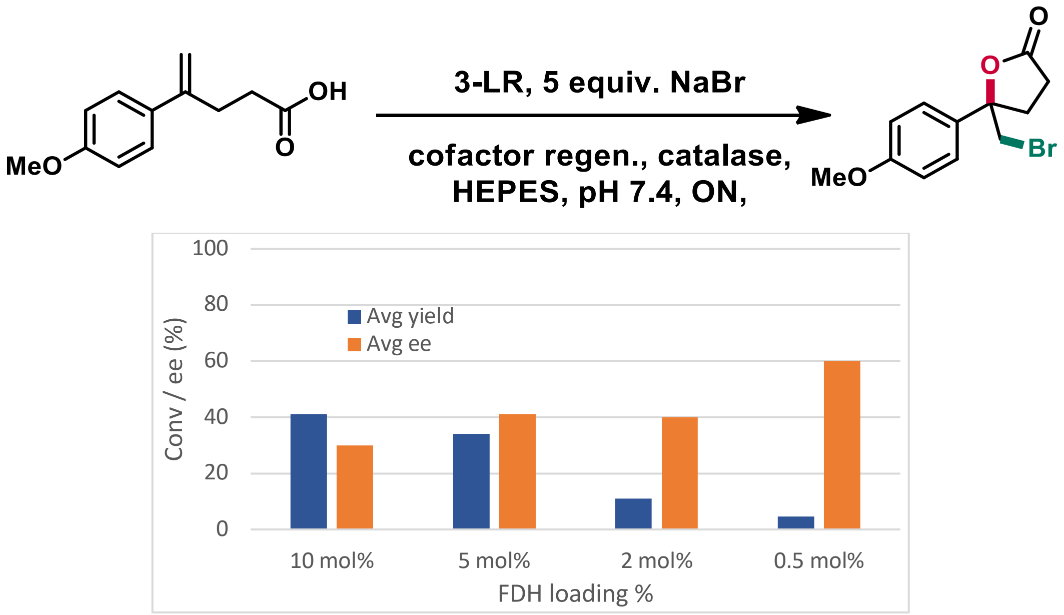

B) **Supplementary Figure 2:** Enantioselectivity of variant 3-LR.

Increasing substrate concentration relative to enzyme substantially improved the enantioselectivity of variant 3-LR, albeit at the expense of product yield.

Substrate **1**:

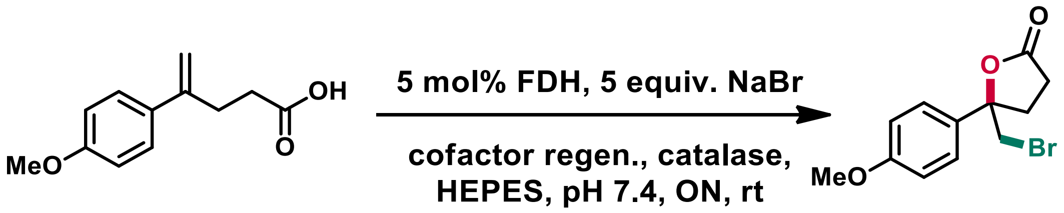

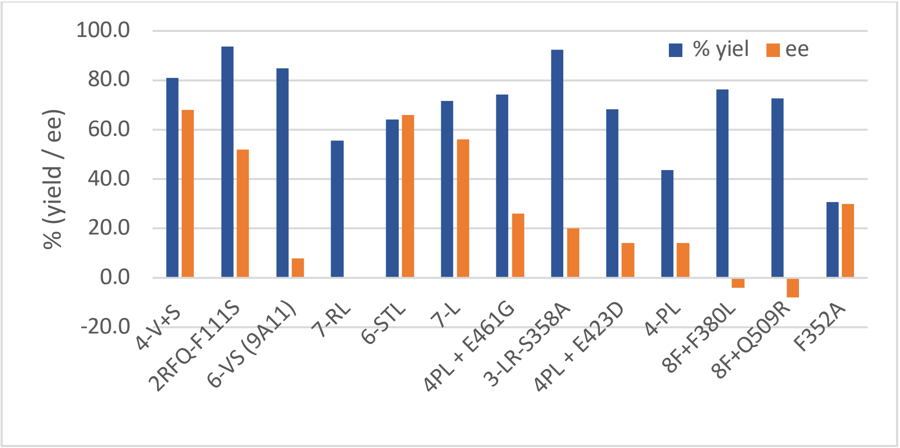

Substrate **2**:

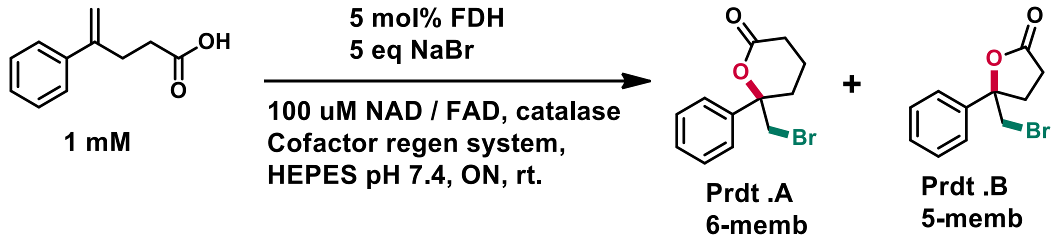

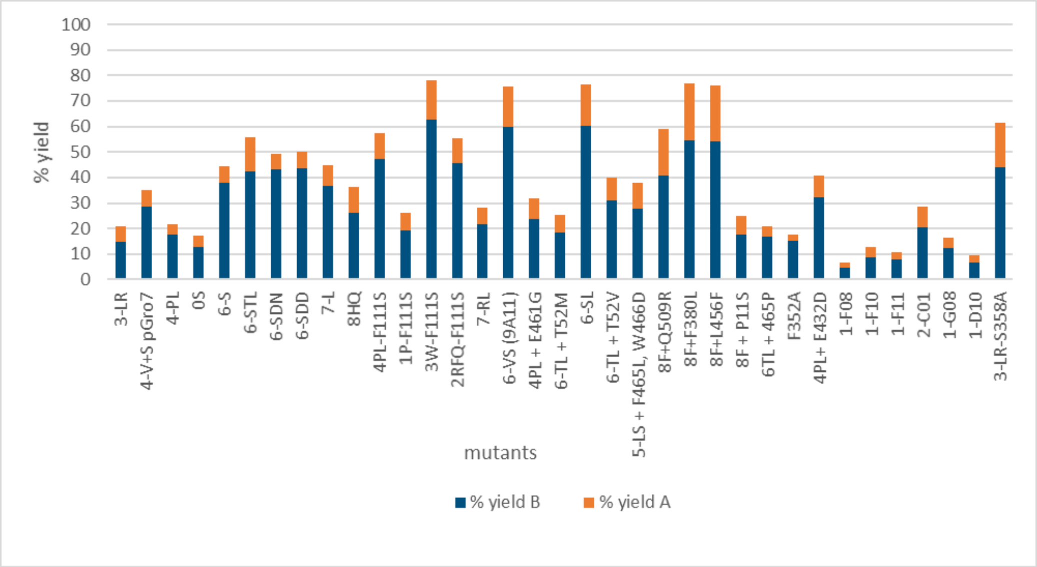

This chart shows the ratios of product A (6-membered lactone) and product B (5-membered lactone).

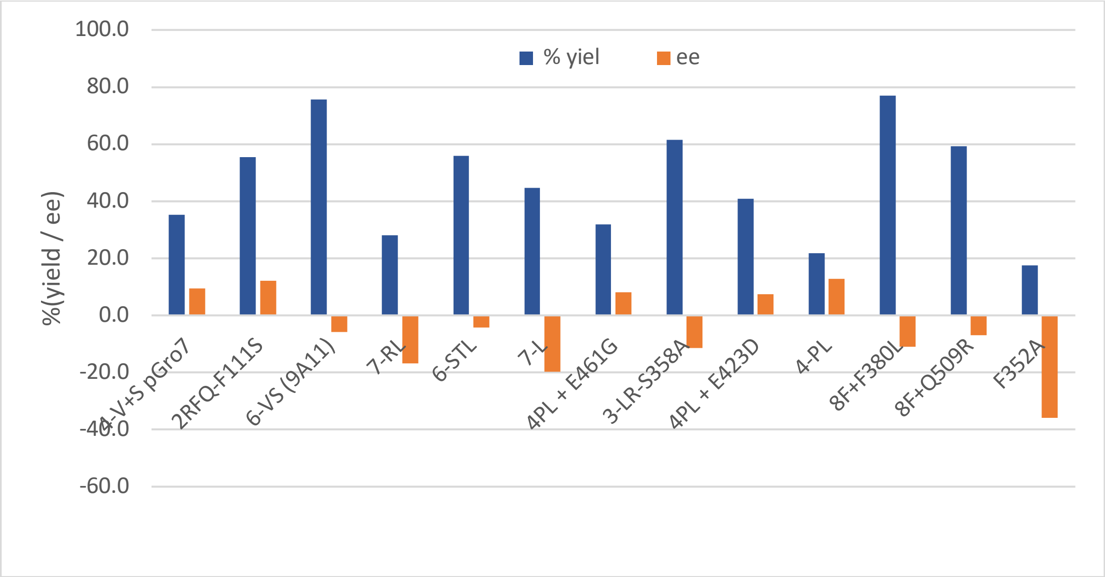

This chart shows total % yield and the ee of the major product (5-membered lactone).

Substrate **3**:

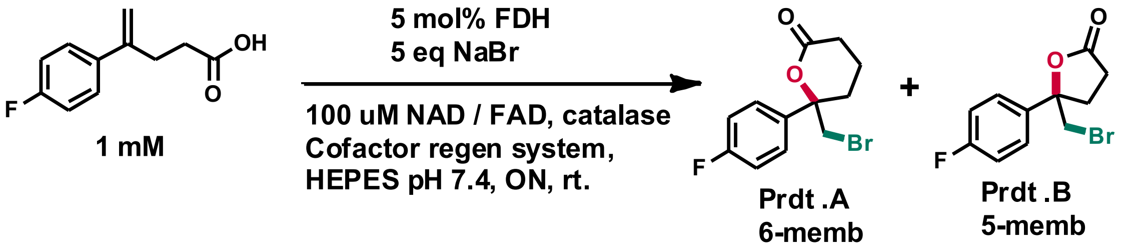

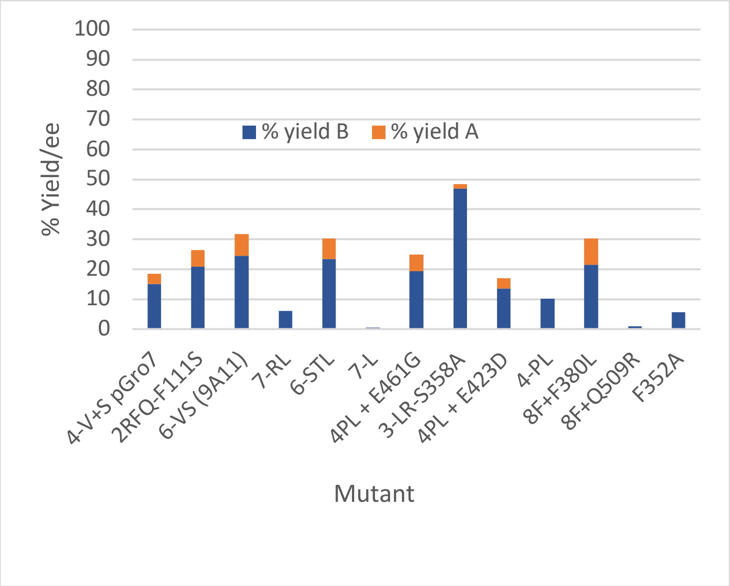

This chart shows the ratios of product A (6-membered lactone) and product B (5-membered lactone).

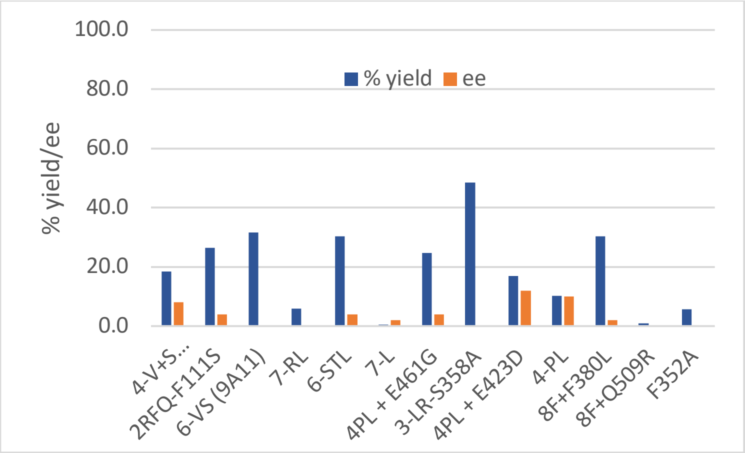

This chart shows total % yield and the ee of the major product (5-membered lactone).

Substrate **4**:

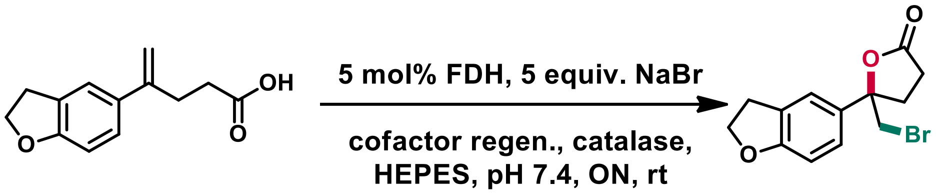

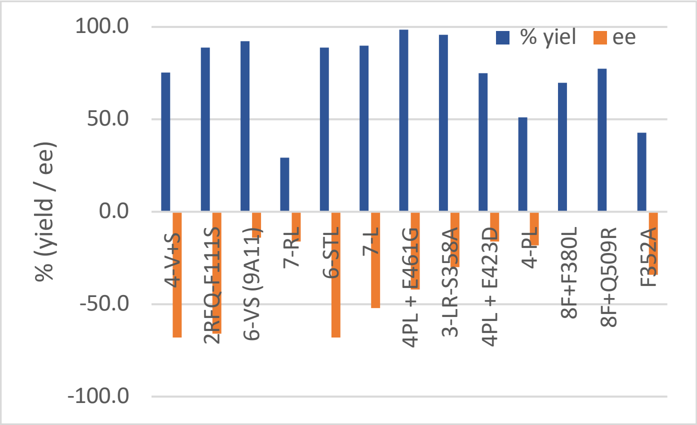

This chart shows total % yield and the ee of the major product (5-membered lactone).

Substrate **5**:

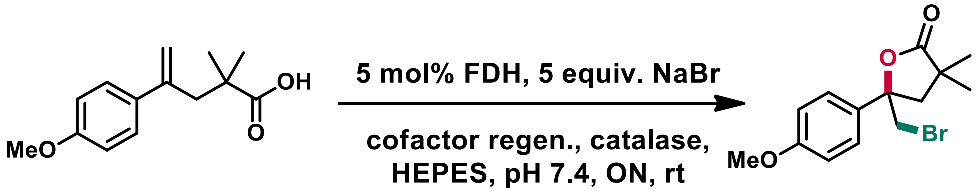

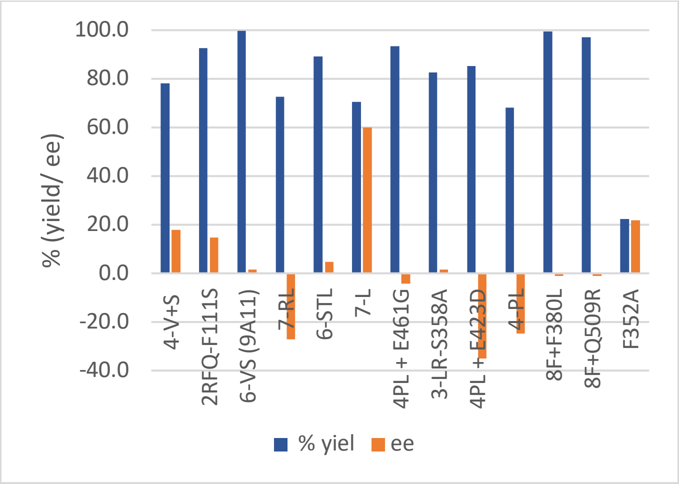

Note, screens were not conducted for substrates **6, 7,** and **8** because suitable activity was observed using previously identified variants.

C) **Supplementary Figure 2:** Activity data for select substrates (1, 2, 4, and 5) using a panel of purified FDHs.

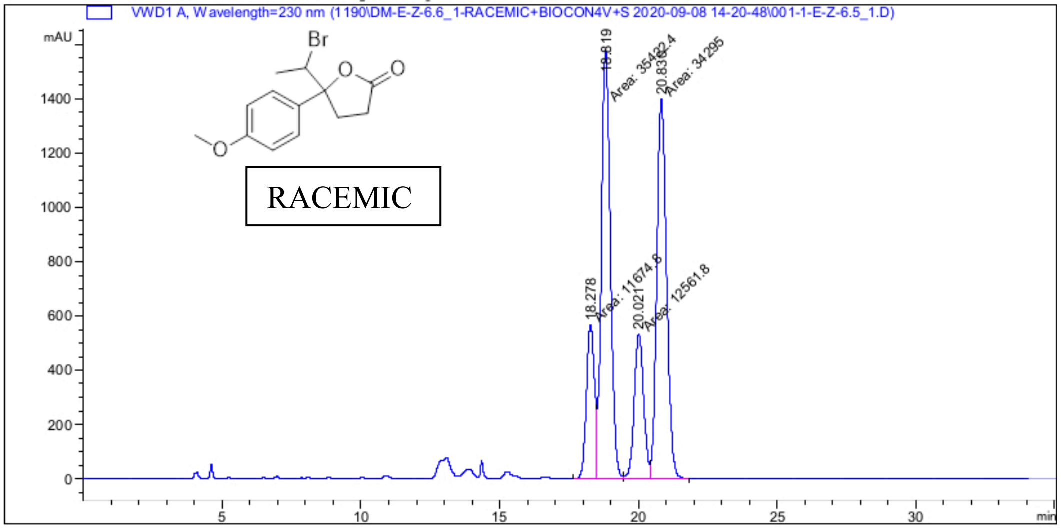

Peak #	RetTime [min]	Type	Width [min]	Area [mAU*s]	Height [mAU]	Area %
1	18.278	MF	0.3423	1.16748e4	568.49451	12.4261
2	18.819	MF	0.3738	3.54224e4	1579.24622	37.7019
3	20.021	FM	0.3923	1.25618e4	533.73810	13.3702
4	20.830	FM	0.4084	3.42950e4	1399.53857	36.5019

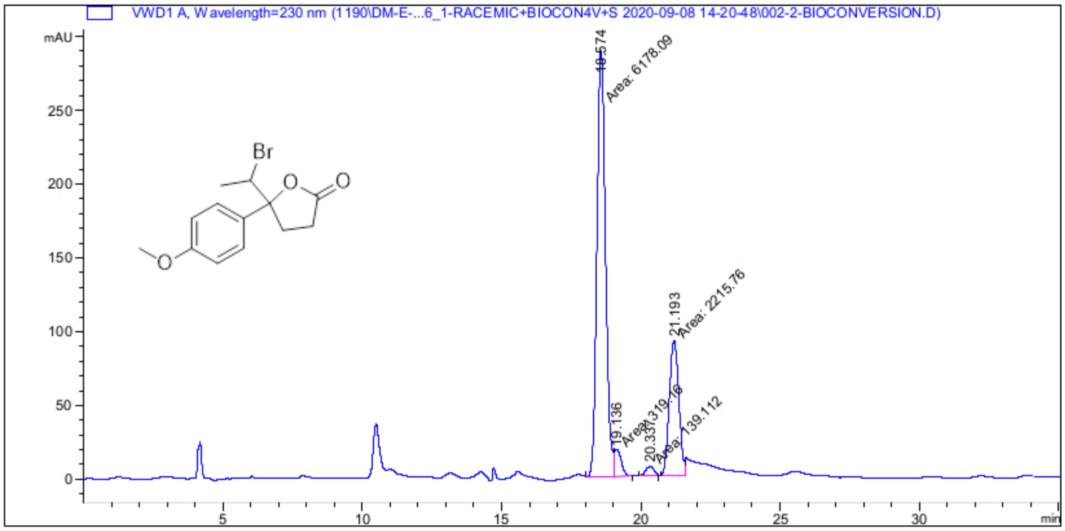

Peak #	RetTime [min]	Type	Width [min]	Area [mAU*s]	Height [mAU]	Area %
1	18.574	MF	0.3566	6178.09424	288.76981	69.7922
2	19.136	FM	0.2954	319.16031	18.00628	3.6055
3	20.337	MM	0.3490	139.11221	6.64306	1.5715
4	21.193	MF	0.4003	2215.75513	92.25744	25.0308

Chiral HPLC analysis (Lux-Cellulose, iPrOH:n-Hex=5/95, flow rate = 0.8 mL/min, l = 230 nm).

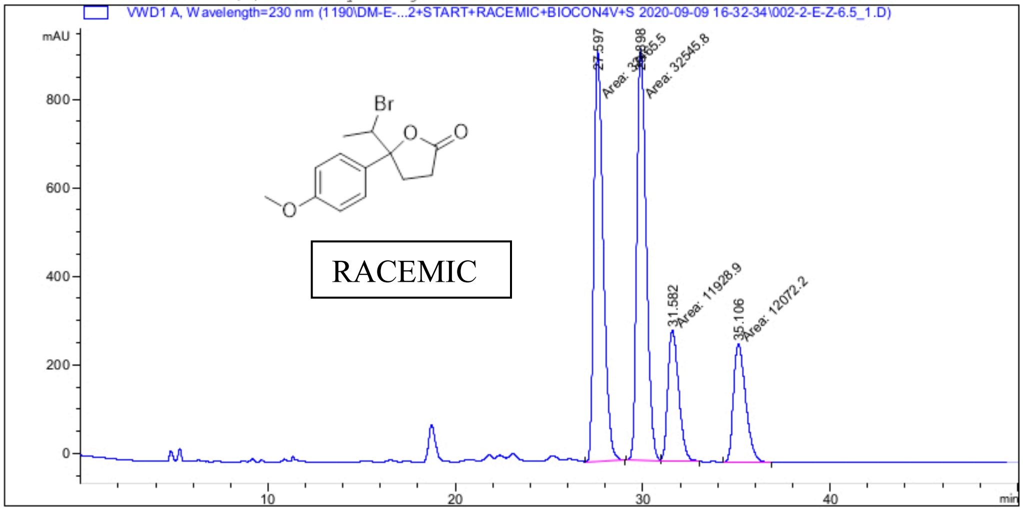

Peak #	RetTime [min]	Type	Width [min]	Area [mAU*s]	Height [mAU]	Area %
1	27.597	MM	0.5830	3.24655e4	928.09882	36.4730
2	29.898	MF	0.5827	3.25458e4	930.81006	36.5632
3	31.582	FM	0.6736	1.19289e4	295.13217	13.4014
4	35.106	MM	0.7563	1.20722e4	266.04025	13.5624

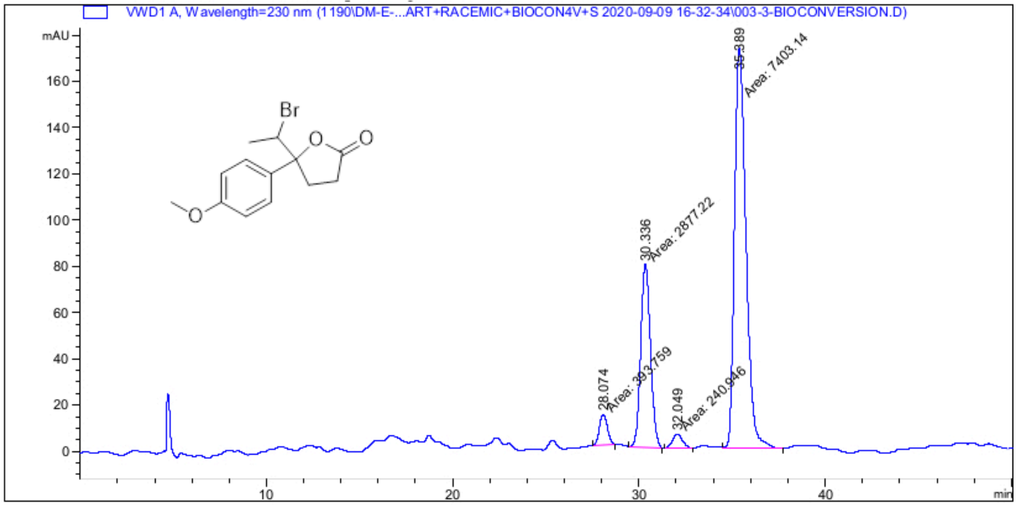

Peak #	RetTime [min]	Type	Width [min]	Area [mAU*s]	Height [mAU]	Area %
1	28.074	MM	0.4921	393.75876	13.33710	3.6075
2	30.336	MM	0.6049	2877.21533	79.27411	26.3600
3	32.049	MM	0.6349	240.94623	6.32468	2.2075
4	35.389	MM	0.7134	7403.14209	172.94783	67.8250

D) **Supplementary Figure 4:** Analysis of the trisubstituted compound.

Chiral HPLC analysis (Chiralpak AD-H, iPrOH:n-Hex=5/95, flow rate = 0.8 mL/min, l = 230 nm).

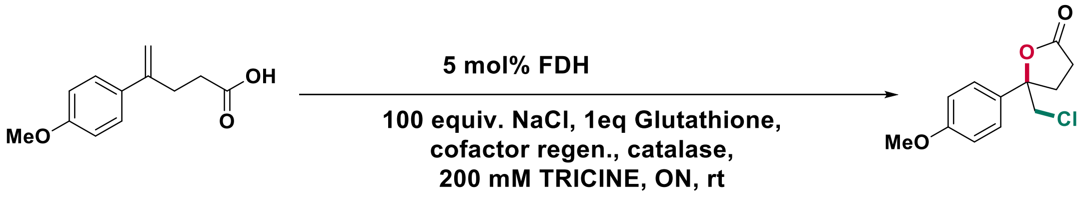

E) **Supplementary Figure 5:** Chlorolactonization activity of FDH variants.

A preliminary screen of several FDH variants that possessed bromolactonization activity revealed no chlorolactonization product for substrate **1**, so a broader screen of FDHs was not evaluated for this reaction. The following variants were examined: 2C01, 7L, 1F10, 1C03, 7RL, 1D11, F352A, 1C08, RebH, E04, 1G08, RadH, 4PL+E423D, 3LR, 8F+Q509R, 1B12, 4PL+E461G, and 01. Notably, 4V+S was not in this set. After 4V+S was identified as the top hit for bromolactonization, it was evaluated for chlorolactonization and found to possess modest activity for this reaction (32% yield, 89:11 e.r. as shown in Section VIII).

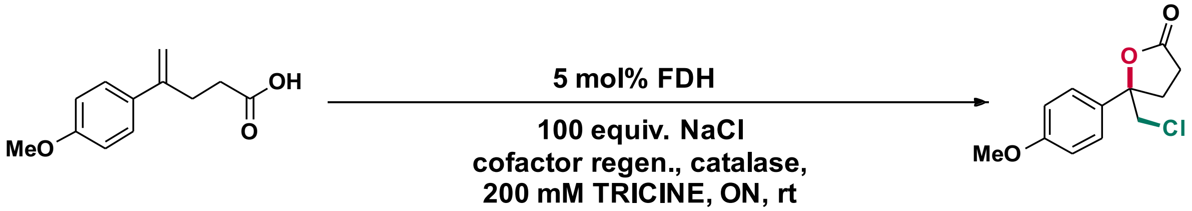

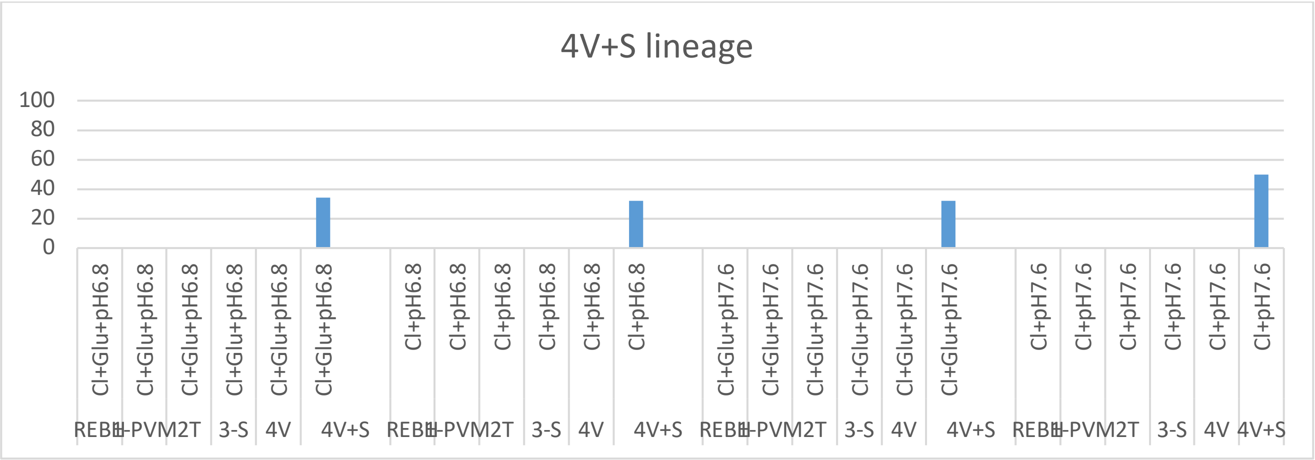

F) **Supplementary Figure 6:** Examination of chlorolactonization by FDH variants in the 4V+S lineage. Only 4V+S possesses chlorolactonization activity.

Reaction mixtures contained 1 mM substrate, 100 equiv. NaCl, 5 mol% FDH, and a cofactor regeneration system comprising a flavin reductase, a glucose dehydrogenase, and glucose. Glutathione (1 mM), catalase, and 200 mM Tricine buffer were used. Product assay yields are the average of triplicate measurements determined by UPLC using 2,6-dimethyl benzonitrile internal standard.

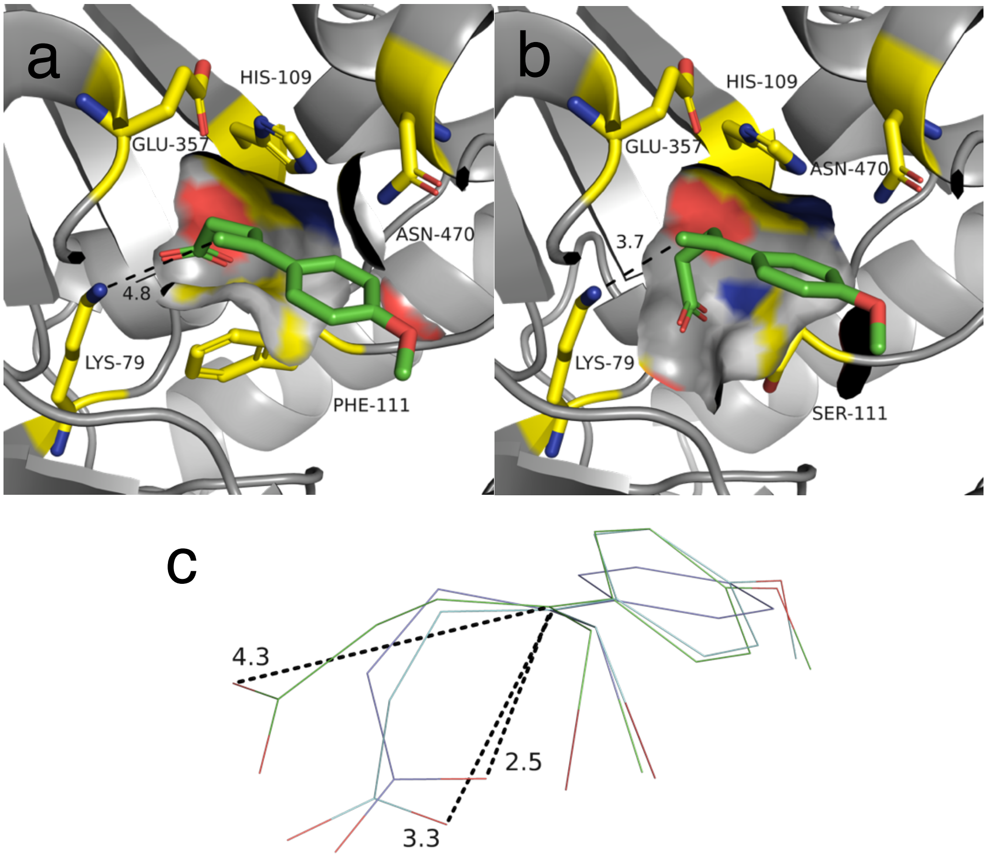

G) **Supplementary Figure 7:** Additional docking poses.

a) Substrate **1** docked into the structure of 3-LSR. b) Substrate **1** docked into the structure of 3-LSR F111S. Note that in addition to orienting the carboxylate moiety in a conformation suitable for halocyclization, the F111S mutation also leads to the substrate binding closer to K79. c) Overlay (C_{phenyl}-C_{benzyl}-C_{CH2X}) of the cationic brominated intermediates derived from substrate **1a** docked into 3-LSR (green) and 3-LSR F111S (cyan) and the calculated transition state for chlorolactonization of the corresponding des-methoxy substrate.[2] The $O_{carboxylate}$-C_{benzyl} distances highlights the similarity of intermediate binding in the F111S mutant to the calculated halocyclization transition state.

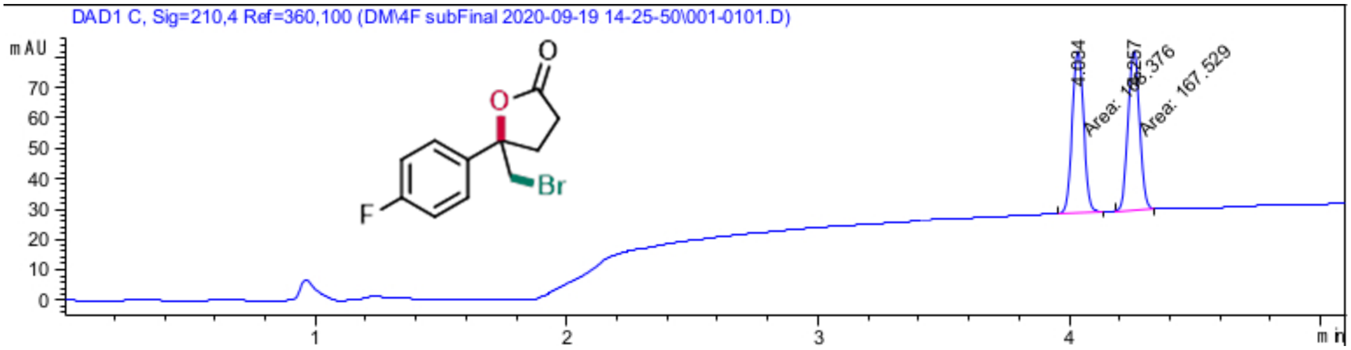

This is the chromatogram of the racemic mixture and analyzed through Chiralpak IC-3 (Part no 83524) column and observed at 210 nm.

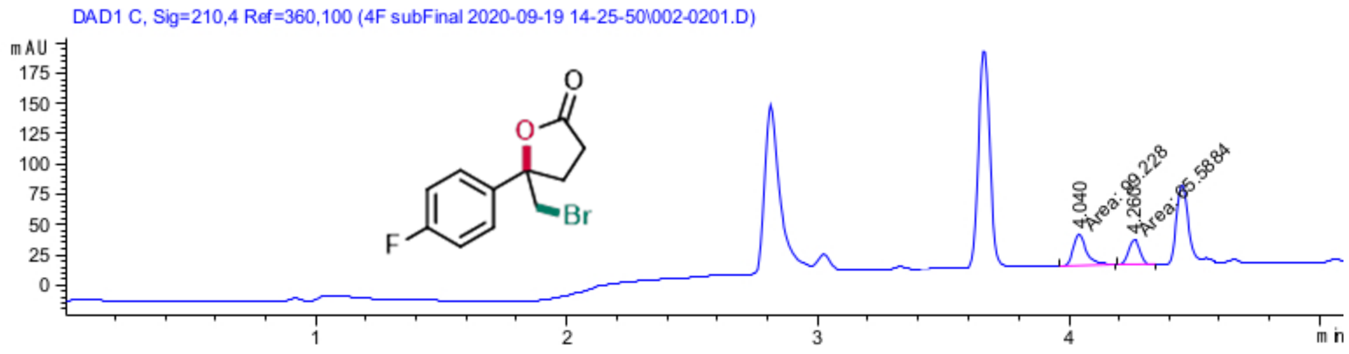

H) **Supplementary Figure 8:** LC analysis of the *p*-fluoro compound **3**.[3]

This is the chromatogram of the reaction mixture catalyzed by 4PL+E423D and analyzed through Chiralpak IC-3 (Part no 83524) column and observed at 210 nm. Reference 3 reported the crystal structure of the *R*-enantiomer of compound **3**. HPLC analysis of the racemic compound in that report using the same column used in our analysis indicated that the *R*-enantiomer eluted before the S-enantiomer. Because we see that the first peak is the larger of the two, we conclude that 4PL+E423D provides predominately the R-enantiomer of compound **3**.

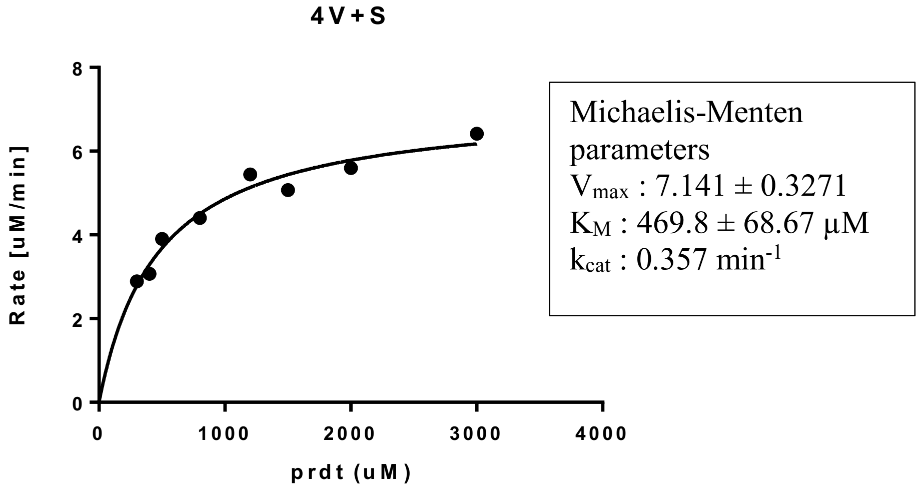

I) **Supplementary Figure 9:** Steady state kinetics for 4V+S bromolactonization.

Determination of kinetic parameters: Rates were determined by monitoring the conversion of substrate 1 in the presence of NAD (100 µM final concentration), FAD (100 µM final concentration), NaBr (5 eq), MBP-RebF (2.5 µM final concentration), glucose dehydrogenase (9 U/mL final concentration GDH), glucose (20 mM final concentration), and *p*-bromoanisole as an internal standard (0.1 mM final concentration) at a final volume of 70 µL in a microtiter plate. 4V+S was added at a final concentration of either 20 µM. Plates were sealed using a plate sealer and shaken at 650 rpm at room temperature. Reaction mixtures were quenched addition of 70 µL of MeOH. All time points were collected in triplicate. The precipitated protein was then removed by centrifugation and the reactions were filtered and analyzed by UPLC method described in Low-Throughput Screening C. Product formation was determined by calculating the ratio of product to internal standard and fitting that value to a calibration curve prepared from known concentrations of product **1a**. The kinetic parameters (K_M and k_{cat}) for 4V+S were determined from the substrate concentrations and the observed initial rates.

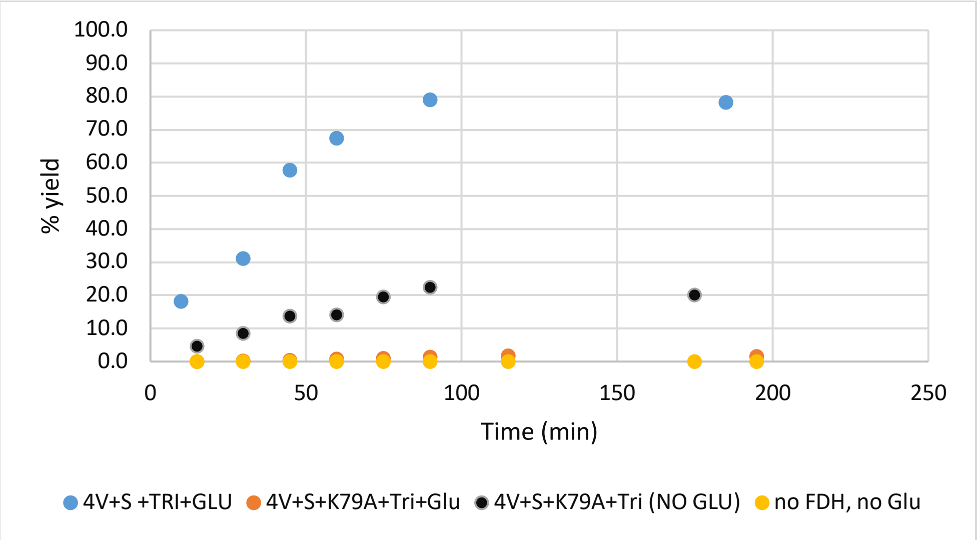

J) **Supplementary Figure 10:** Comparison time courses for bromolactonization of **1** by 4V+S variants.

This figure shows that in absence of glutathione, bromolactonization activity for the K79A mutant was observed, but the reaction was significantly slower than that of 4V+S in the presence of glutathione. No reaction was observed in the absence of FDH, indicating that this reaction did not result from side reactions involving reduced flavin cofactor, O_2, and halide ions in solution. Conditions are the same as those used in manuscript Figure 3 with modifications as noted in the graph.

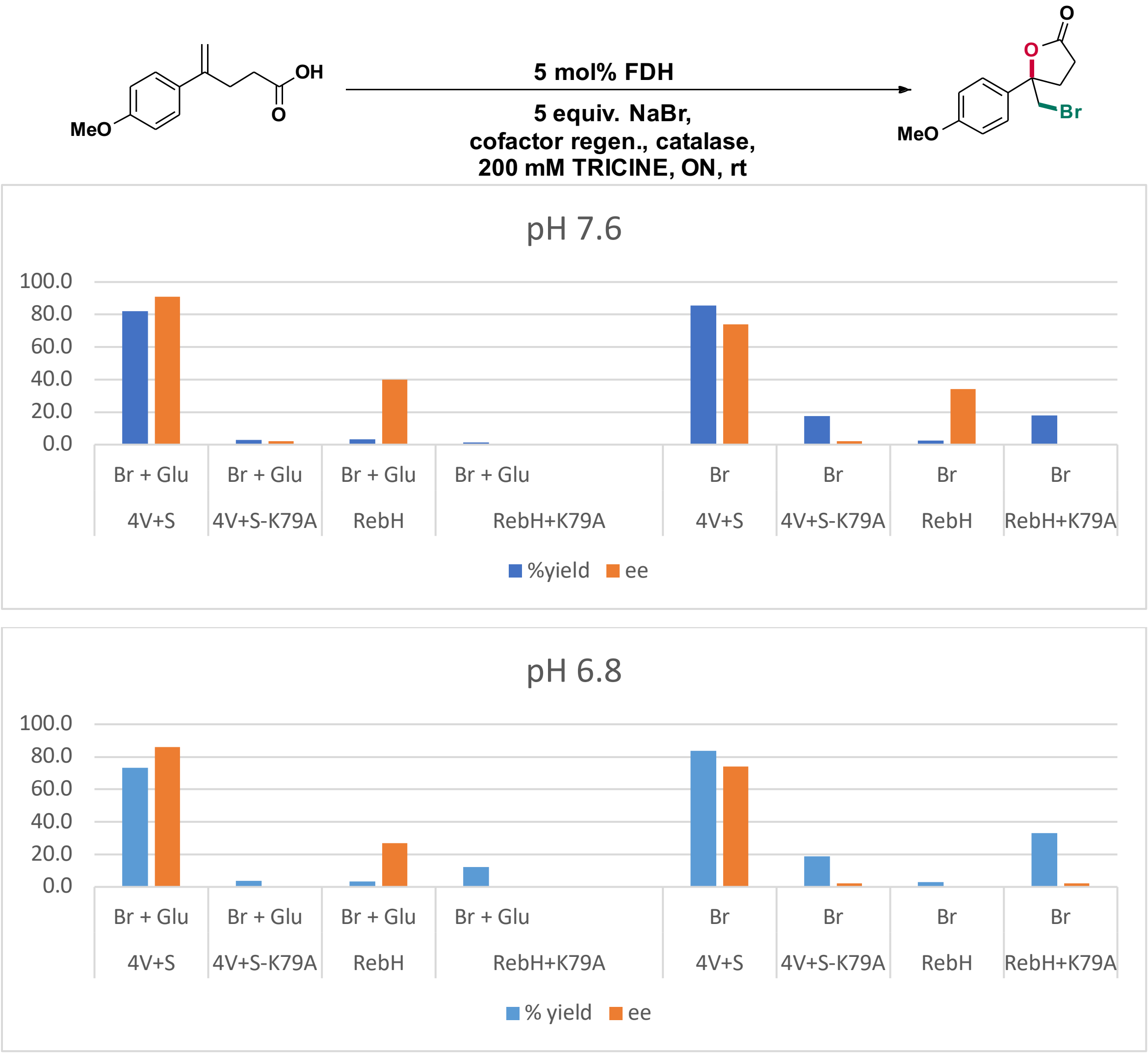

K) **Supplementary Figure 11:** Comparison of bromolactonization by 4V+S, RebH, and their K79A mutants.

Reaction mixtures contained 1 mM substrate, 5 equiv. NaBr, 5 mol% FDH, and a cofactor regeneration system comprising a flavin reductase, a glucose dehydrogenase, and glucose. Glutathione (1 mM), catalase, and 200 mM Tricine buffer were used. Product assay yields and selectivities are the average of triplicate measurements determined by UPLC using 2,6-dimethyl benzonitrile internal standard.

4V+S provides optimal yield and enantioselectivity in the presence of glutathione. The K79A mutant of this enzyme provides a modest yield of essentially racemic product in the absence of glutathione, which we ascribed to reaction by free HOBr. RebH and its K79A variant provide only trace product in the presence of glutathione, but a similar yield of racemic product (~20%) is observed for the K79A variants of both 4V+S and RebH in the absence of glutathione. The bromolactonization results for 4V+S and its K79A variant in the absence of glutathione show that the active brominating species in FDH catalysis provides greater conversion than HOBr released by the enzyme. The bromolactonization results for 4V+S in the presence and absence of glutathione show that the small amount of racemic product proposed to

result from HOBr released from the enzyme (with K79 present) in the absence of glutathione is sufficient to significantly erode product enantioselectivity. In most cases, slightly higher yields are observed for the K79A mutants at pH 6.8 relative to 7.6, which is consistent with the $[HOBr]/[BrO^-]$ ratio expected at the two pH values (79:1 and 13:1, respectively). While one might expect the putative racemic background reaction to be exaggerated with the K79A mutants, the bromomactonization results show that this is not necessarily the case. RebH K79A indeed provides a significantly higher yield of racemic bromolactonization product (18%) relative to the trace observed with the wt enzyme in the absence of glutathione. On the other hand, significantly higher bromolactonization yield is observed for 4V+S relative to 4V+S K79A (86% vs 17%) in the absence of glutathione at pH 7.6 (similar at pH 6.8). We suspect that this difference reflects the significantly improved ability of the 4V+S active site to accommodate halocyclization relative to RebH.

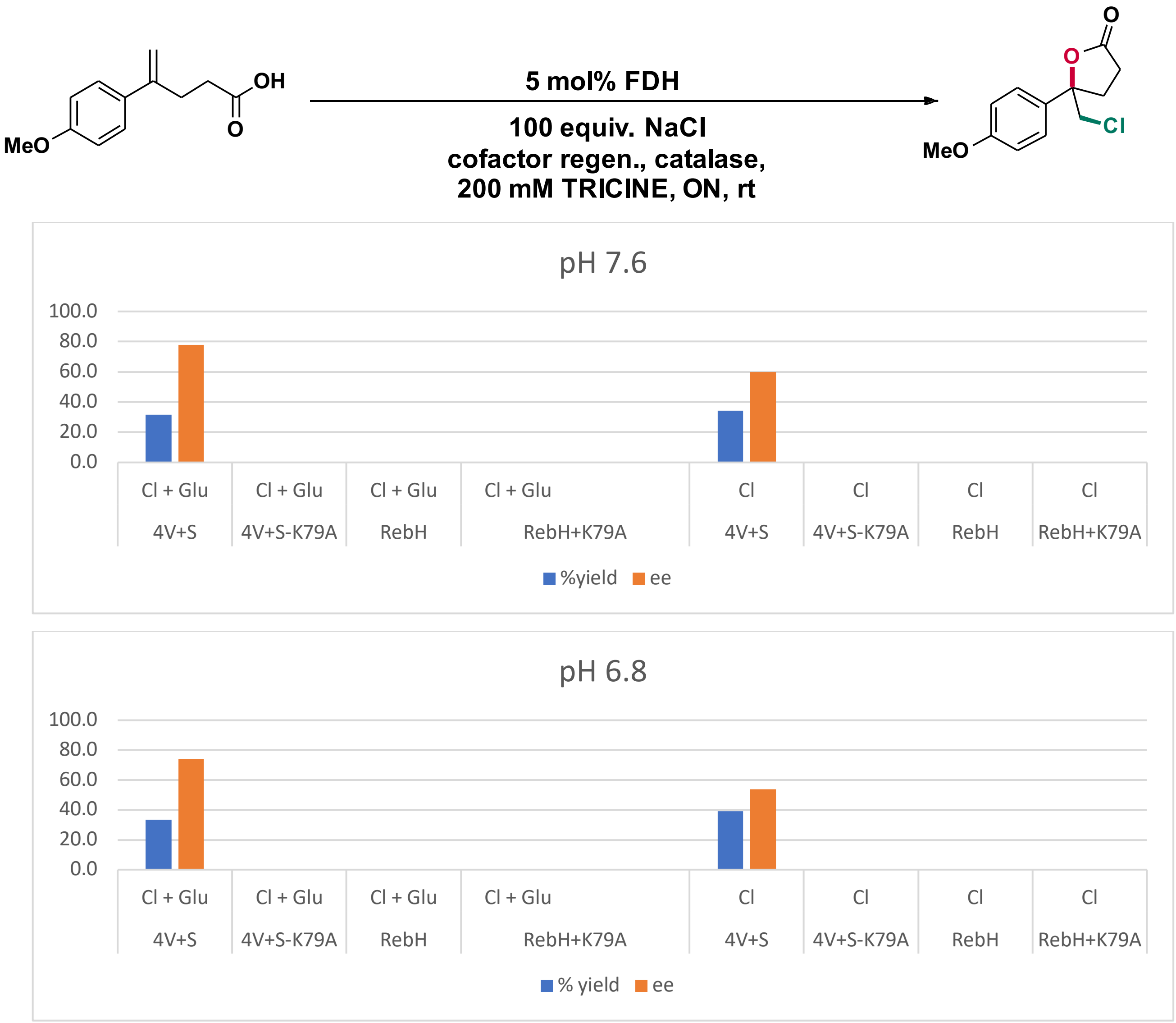

L) **Supplementary Figure 12:** Comparison of chlorolactonization by 4V+S, RebH, and their K79A mutants.

Reaction mixtures contained 1 mM substrate, 100 equiv. NaCl, 5 mol% FDH, and a cofactor regeneration system comprising a flavin reductase, a glucose dehydrogenase, and glucose. Glutathione (1 mM), catalase, and 200 mM Tricine buffer were used. Product assay yields and selectivities are the average of triplicate measurements determined by UPLC using 2,6-dimethyl benzonitrile internal standard.

Chlorolactonization was only observed for 4V+S, and while similar yields were obtained under all conditions evaluated, a significant reduction in enantioselectivity was observed in the absence of glutathione. It is notable that no chlorolactonization is observed for 4V+S K79A (or RebH K79A) in the absence of glutathione at pH 7.6 or 6.8. On the other hand, 4V+S only provides 35% yield for chlorolactonization in the absence of glutathione. A similar (80%) reduction in yield for the corresponding K79A variant as observed for bromolactonization would lead to a chlorolactonization yield of around 7%. However, the expected [HOX]/[XO^-] ratio is ~15 times higher for X=Br than for X=Cl at the pH values used, which could explain the lack of racemic background reaction observed for the latter. It is not currently clear why chlorolactonization enantioselectivity decreases in the absence of glutathione since there does not seem to be sufficient racemic background reaction based on the 4V+S K79A results to account for the observed decrease.

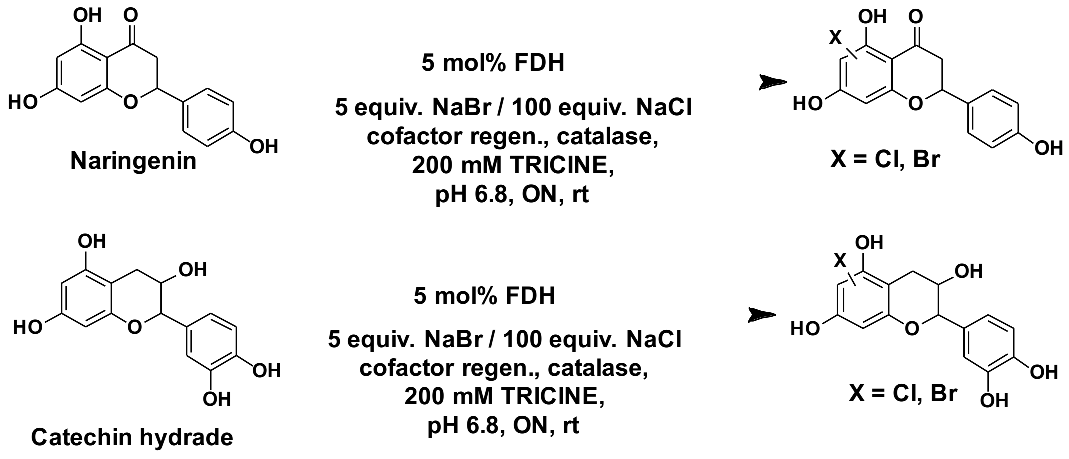

Reaction mixtures contained 1 mM substrate, 100 equiv. NaCl, 5 mol% FDH, and a cofactor regeneration system comprising a flavin reductase, a glucose dehydrogenase, and glucose. Catalase, and 200 mM Tricine buffer pH 6.8 were used. Two brominated products and a single chlorinated product are observed in both cases. Conversions were determined by % Conv = {(AUC of P1 + AUC of P2)/(AUC of St)}*100. Regioisomeric percentages were determined by % P1 = {(AUC of P1) / (AUC of P1 + AUC of P2))} *100.

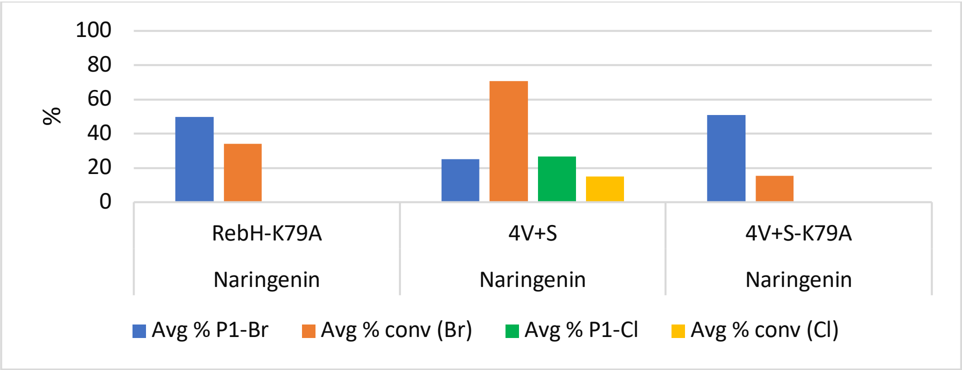

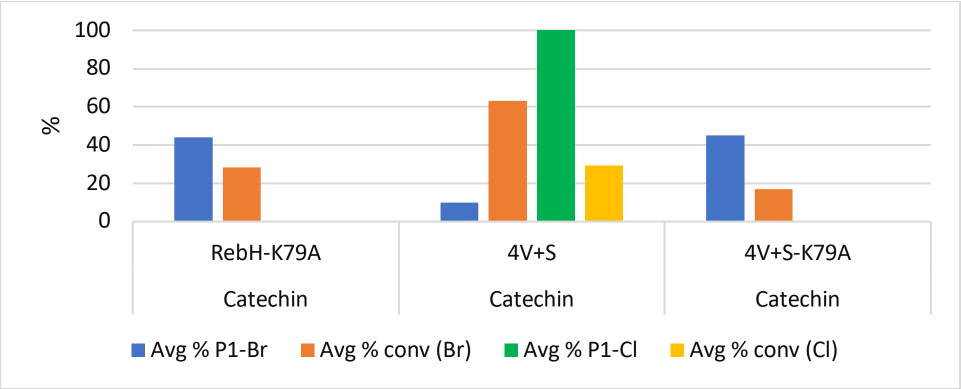

M) **Supplementary Figure 13:** RebH-K79A can brominate electron rich substrates.

This result shows that RebH K79A and 4V+S K79A are both active aromatic bromination catalysts. Their identical bromination selectivity (%P1-Br) with one another and their different selectivity than 4V+S suggest that they can both release HOBr into solution.

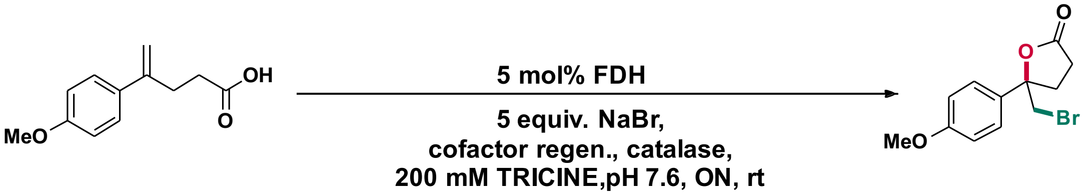

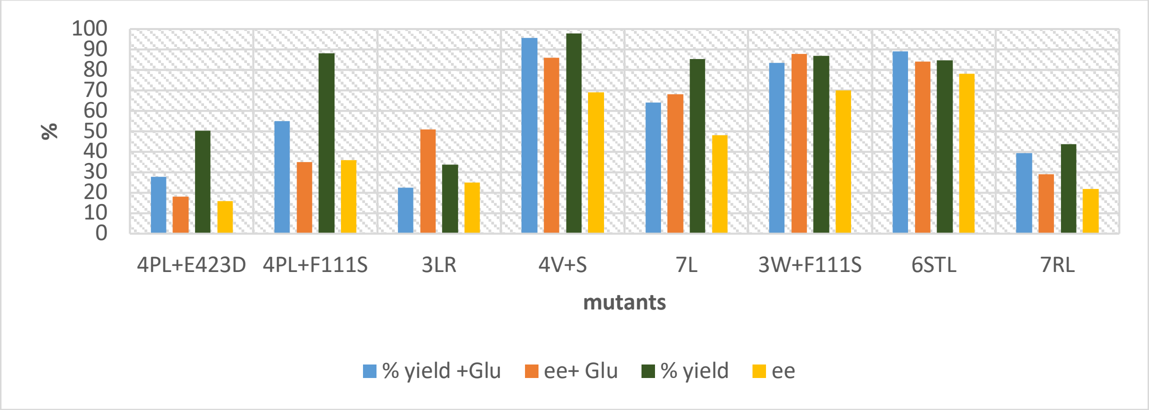

N) **Supplementary Figure 14:** Improved selectivity for bromolactonization in the presence of glutathione.

Reaction mixtures contained 1 mM substrate, 5 equiv. NaBr, 5 mol% FDH, and a cofactor regeneration system comprising a flavin reductase, a glucose dehydrogenase, and glucose. Glutathione (1 mM), catalase, and 200 mM Tricine buffer were used. Product assay yields and selectivities measurements were determined by UPLC using 2,6-dimethyl benzonitrile internal standard.

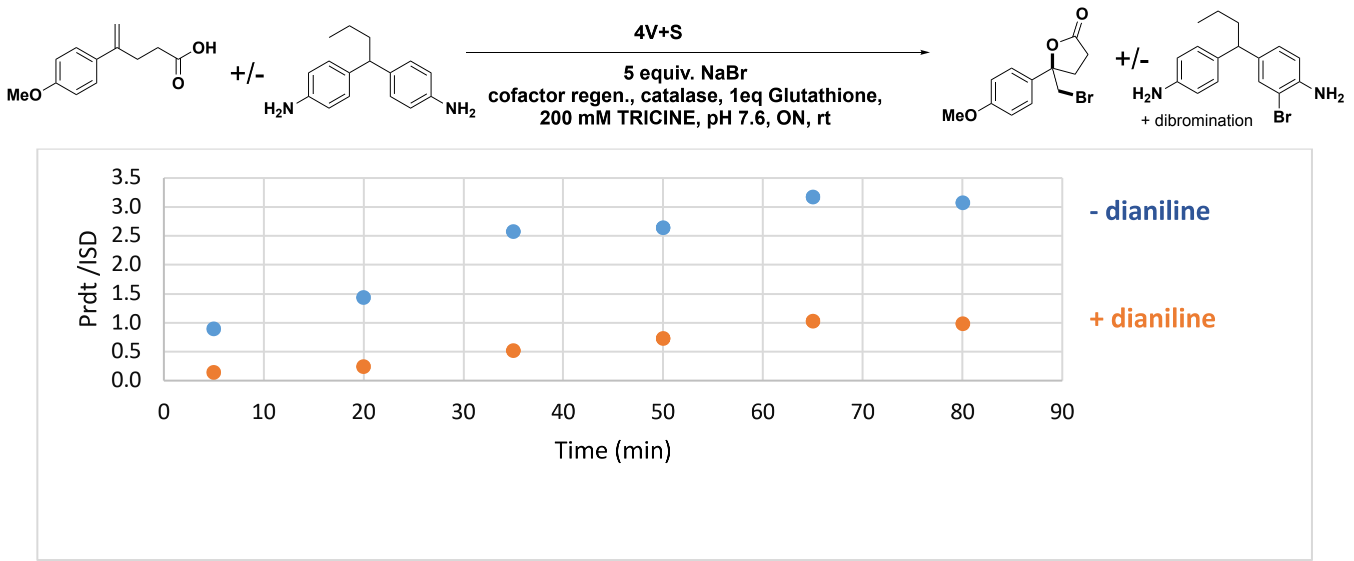

O) **Supplementary Figure 15:** Time course experiment for bromolactonization in the presence of a previously reported 4V+S substrate (manuscript reference 29).

Reaction mixtures contained 1 mM substrate, 5 equiv. NaBr, 5 mol% FDH, and a cofactor regeneration system comprising a flavin reductase, a glucose dehydrogenase, and glucose. Glutathione (1 mM), catalase, and 200 mM Tricine buffer were used. Bromolactonization product assay yields were determined by LC-MS using 2,6-dimethyl benzonitrile internal standard.

VI. Supplementary Synthetic Procedures

A) General synthetic procedures of Substrates 1,2,3,4 and 6.[4,3]

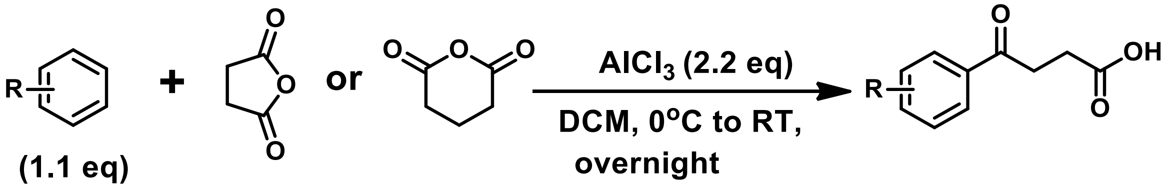

To an oven-dried 250mL round-bottomed flask containing succinic anhydride (30 mmol) or glutaric anhydride (30 mmol) and arene (33 mmol, 1.1 equiv.) in DCM (100 mL) was added $AlCl_3$ (66 mmol, 2.2 equiv.) portion-wise at room temperature over 5 minutes. The reaction mixture was allowed to stir until complete conversion was observed by TLC. The reaction was poured over 1N HCl on ice, extracted with ether (200 mL), and washed with 1N HCl (3 x 50 mL). The organic layer was dried ($MgSO_4$) and

concentrated to give the product as a solid that was subsequently washed with hexanes and vacuum filtered to afford the crude product.

B) General synthetic procedure for the Wittig reaction.[4,3]

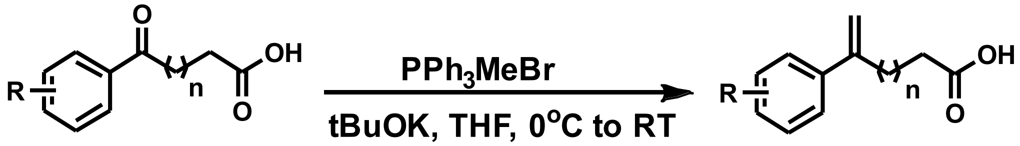

PPh_3MeBr (21.89 mmol) was added to a suspension of tBuOK (43.78 mmol) in THF (42 mL, 0.4 M) at 0 °C, and the mixture was stirred for 30 min. Substituted 4-oxo-4-phenylbutanoic acid (3.00 g, 16.84 mmol) was then added to the reaction mixture at 0 °C, and the resulting mixture was allowed to warm to rt and stirred for 16 h. After evaporation of THF, CH_2Cl_2 (25 mL) and NaOH (1N, 25 mL) were added. The aqueous layer was washed with CH_2Cl_2 (3 x 30 mL) and acidified with HCl (12 N). The aqueous layer was then extracted with CH_2Cl_2 (3 x 30 mL) and the combined organic portions were dried and concentrated to give a substituted 4-phenylpent-4-enoic acid that was used without further purification. The characterization data was in complete agreement with that previously reported in the literature.[3,4]

Analytical data for substrate **1** (^{1}H NMR spectrum matches reported spectrum)[4]:

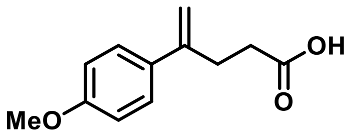

^{1}H NMR (DMSO, 400 MHz): δ 7.33 (2H), 6.84 (2H), 5.20 (1H), 4.99 (1H), 3.77 (3H), 2.76 (2H), 2.40 (2H).

Analytical data for substrate **2** (^{1}H NMR spectrum matches reported spectrum)[4]:

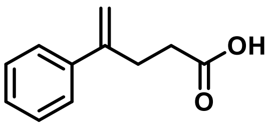

^{1}H NMR ($CDCl_3$, 400 MHz): δ 7.39 (2H), 7.33 (2H), 7.29 (1H), 5.32 (1H), 5.11 (1H), 2.87 (2H), 2.54 (2H).

Analytical data for substrate **3** (^{1}H NMR spectrum matches reported spectrum)[4]:

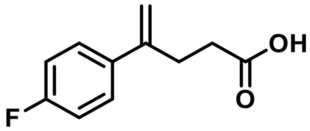

^{1}H NMR (DMSO, 400 MHz): δ 7.39 (2H), 7.02 (2H), 5.24 (1H), 5.07 (1H), 2.77 (2H), 2.41 (2H).

Analytical data for substrate **4**:

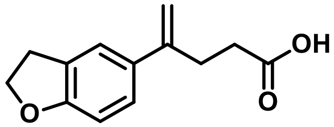

^{1}H NMR, ($CDCl_3$, 500 MHz): δ 7.27 (1H), 7.16 (1H), 6.75 (1H), 5.21 (1H), 4.98 (1H), 4.56 (2H), 3.20 (2H), 2.80 (2H), 2.52 (2H). ^{13}C NMR (126 MHz, $CDCl_3$): δ 179.65, 159.94, 146.41, 133.09, 127.31, 126.09, 122.86, 111.27, 109.11, 71.53, 33.22, 30.54, 29.78. HRMS: Calculated:218.094, Found, $(M-H)^-$: 217.086.

Analytical data for substrate **5**:

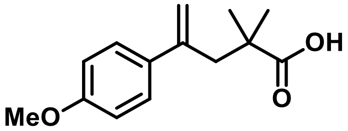

^{1}H NMR ($CDCl_3$, 500 MHz): δ 9.12 (1H), 7.22 (2H), 6.74 (2H), 5.16 (1H), 4.96 (1H), 3.72 (3H), 2.74 (2H), 1.13 (6 H). ^{13}C NMR (126 MHz, $CDCl_3$): δ 184.06, 158.97, 145.20, 134.84, 127.76, 115.66, 113.43, 55.11, 45.19, 42.73, 25.13. HRMS: Calculated:234.126, Found, $(M-H)^-$:233.117.

Analytical data for substrate **6** (^{1}H NMR spectrum matches reported spectrum)[4]:

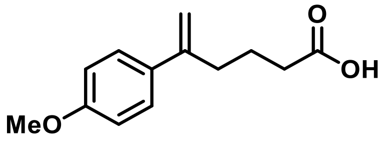

^{1}H NMR (DMSO, 400 MHz): δ 7.37 (2H), 6.88 (2H), 5.24 (1H), 5.10 (1H), 3.81 (3H), 2.56 (2H), 2.32 (2H), 1.74 (2 H).

C) General synthetic procedure for Synthesis of Substrate **8**.[5]

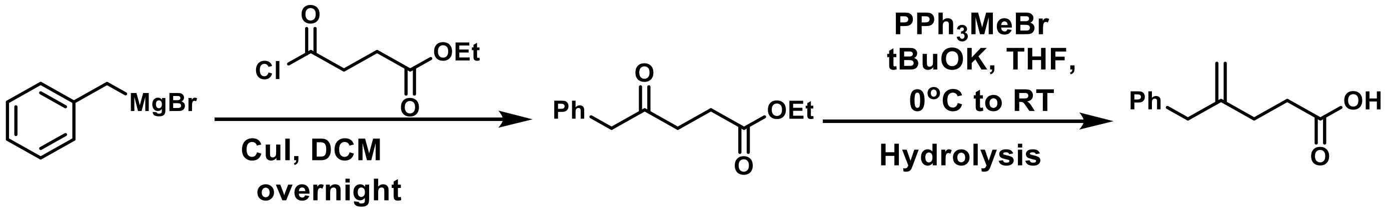

Compound **8** was prepared according to the reported procedure.[5] A solution of 1.0 M BnMgCl in THF (60 mL, 60 mmol) was added to a suspension of CuI (5.71 g, 30 mmol) and ethyl succinylchloride (5.36 g, 30 mmol) in THF (60 mL) at 0 °C. After stirring for 3 h at room temperature, the reaction was quenched with saturated aqueous NH_4Cl. The mixture was extracted with ethyl acetate (3 x 50 mL), dried over anhydrous Na_2SO_4, filtered, and concentrated. The resulting oil was then purified by column chromatography on silica gel (hexane–EtOAc 10:1) to give ethyl 4-oxo-5-phenylpentanoate as pale-yellow oil (3.5 g). $MePPh_3Br$ (9.76 g, 27.3 mmol) was added to a suspension of tBuOK (4 g, 36.4 mmol) in THF (50 mL) at 0 °C, and the mixture was stirred for 30 min. At the same temperature, a solution of ethyl 4-oxo-5-phenylpentanoate (3.5 g, 18.2 mmol) in THF (5 mL) was added. The mixture was warmed to ambient temperature and stirred overnight. After evaporation of THF, CH_2Cl_2 (25 mL) and NaOH (1N, 25 mL) were added. The aqueous layer was washed with CH_2Cl_2 (3 x 30 mL) and acidified with HCl (12 N) to a pH = 2. The aqueous layer was then extracted with CH_2Cl_2 (3 x 30 mL) and the combined organic portions were dried and concentrated to give ethyl 4-benzylpent-4-enoate. To a solution of ethyl 4-benzylpent-4-enoate (671 mg, 2.9 mmol) in MeOH (6 mL) was added a solution at 0 °C. After stirring for 2 h at ambient of KOH (811 mg, 14.5 mmol) in H_2O (1.2 mL) temperature, the reaction was quenched with concentrated aqueous HCl. The mixture was dried over anhydrous Na_2SO_4, filtered and extracted with EtOAc. The organic layers concentrated. The residue was purified by column chromatography on silica gel (hexane–EtOAc 5:1) to give **8** as pale-yellow oil. (350 mg). The characterization data was in complete agreement with that previously reported in the literature[5]: ^{1}H NMR ($CDCl_3$, 400 MHz): δ 7.30-7.17 (5H), 4.83 (2H), 3.36 (2H), 2.49 (1H), 2.30 (1H).

D) General synthetic procedure for Synthesis of Substrate **7**:[6]

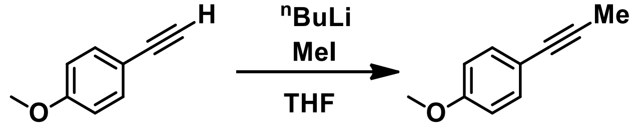

4-Ethynylanisole
1

2

Compound **2** was prepared by adapting the reported procedure.[6] To an oven-dried 50 mL round-bottomed flask flushed with nitrogen was added 422 mg 4-Ethynylanisole (3.20 mmol, 1.00 equiv.) and THF (4 mL). The reaction mixture was cooled to -20 °C and 2.8ml *n*-BuLi (2.0 M in n-hexane, 4.50 mmol, 1.40 equiv.) was added dropwise with stirring. The solution was stirred at -20 °C for 1 h, and 1.32 g MeI (9.30 mmol, 3.00 equiv.) was added to the solution dropwise to maintain a temperature below -18 °C. The solution was stirred at room temperature for 8 h. The reaction mixture was quenched with saturated NH_4Cl and extracted with ethyl acetate. The combined organic layers were washed with brine, dried over Na_2SO_4, and concentrated. The compound **2** was obtained in 84% yield (390 mg) as clear oil after flash column chromatography using hexanes as eluent.

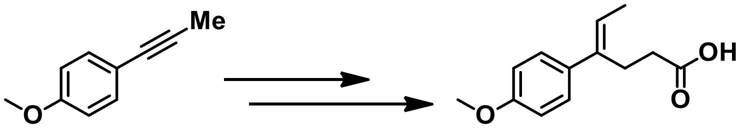

2

Substrate 7

Compound **7** was prepared according to the reported procedure.[7] To a suspension of 390 mg 1-methoxy-4-(prop-1-yn-1-yl)benzene (2.67 mmol, 1.00 equiv.) and 1.35 g sodium iodide (9 mmol, 3.30 equiv.) in acetonitrile (20 ml) was added 1.66 g cerium chloride heptahydrate (4.50 mmol, 1.70 equiv.). The mixture was heated at reflux for 24h. The reaction mixture was diluted with ethyl acetate and 0.5% HCl. The mixture was extracted with ethyl acetate, washed by saturated $NaHCO_3$ and saturated NaS_2O_3, and then dried over $MgSO_4$. The product was afforded in 45% yield (329 mg) as yellow oil by flash column chromatography using hexane as eluent.

To a solution of 160 mg 1-(1-iodoprop-1-en-1-yl)-4-methoxybenzene (0.58 mmol, 1.00 equiv.) and 19.7 mg Tetrakis(triphenylphosphine) palladium (0) (0.017 mmol, 0.03 equiv.) in THF (5 ml) under nitrogen atmosphere in ice bath was added 2.34 ml 3-ethoxy-3-oxopropylzinc bromide solution (1.16 mmol, 2.00 equiv.). The mixture was stirred at 50 °C for 6 h. The reaction was quenched by addition of saturated NH_4Cl and extracted with ethyl acetate. The combined extracts were dried over $MgSO_4$. The product was

obtained in 72% yield (104 mg) as yellow oil after flash column chromatography using hexanes/ethyl acetate as eluent.

To a solution of 52 mg ethyl-4-(4-methoxyphenyl) hex-4-enoate (0.21 mmol, 1.00 equiv.) in methanol (2 ml) was added 1M NaOH (1.5 ml). The reaction mixture was stirred at room temperature for 4 h. 1M HCl was added into the reaction mixture until pH<2. The product 7 was obtained in 91% yield (42 mg) as yellow solid after flash column chromatography using hexanes/ethyl acetate (v/v=10/1-1/1) as eluent.

Analytical data for compound **7** (major isomer):
^{1}H NMR, $CDCl_3$, 500 MHz: 7.18 (2H), 6.78 (2H), 5.64 (1H), 3.76 (3H), 2.78 (2H), 2.31 (2H), 1.76 (3H). ^{13}C NMR (126 MHz, $CDCl_3$): δ 179.81, 158.72, 138.19,129.78, 127.42, 123.06, 113.87, 55.38, 33.00, 24.71, 14.14. HRMS: Calculated: 220.110, Found, $(M-H)^-$: 219.102.

E) General synthetic procedure for synthesis of racemic standards

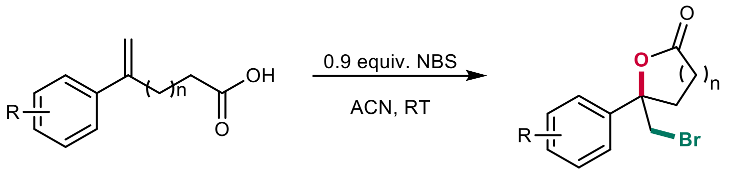

Authentic racemic products were prepared according to a reported procedure.[2] To a solution of alkenoic acid (0.1 mmol, 1.0 equiv) and ACN (5 mL) was added *N*-bromosuccinimide (0.9 equiv). The resulting mixture was stirred at RT and monitored by TLC. Upon completion of reaction, the reaction was concentrated *in vacuo*. The residue was purified by flash column chromatography to yield the corresponding lactone. NMR data for the resulting products matched those reported in the literature.[2,6,7]

Product **1a**:

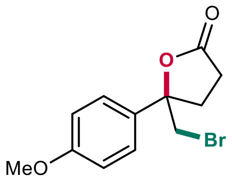

^{1}H NMR, (DMSO, 400 MHz): δ 7.36 (2H), 6.96 (2H), 3.88 (1H), 3.82 (3H), 3.80 (1H), 2.80 (2H), 2.56 (2H).

Product **1c**:

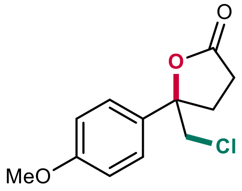

^{1}H NMR, ($CDCl_3$, 600 MHz): δ 7.32 (2H), 6.91 (2H), 3.82 (1H), 3.80 (3H), 3.80 (1H), 2.80 (2H), 2.55 (2H).

Product **2a**:

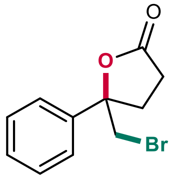

^{1}H NMR, ($CDCl_3$, 500 MHz): δ 7.43-7.38 (5H), 3.76 (1H), 3.70 (1H), 2.84 (1H), 2.57 (1H).

Product **3a**:

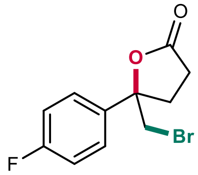

^{1}H NMR, ($CDCl_3$, 400 MHz): δ 7.40 (2H), 7.10 (2H), 3.70 (1H), 3.64 (1H), 2.84 (1H), 2.53 (1H).

Product **4a**:

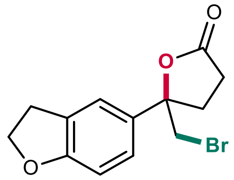

^{1}H NMR, ($CDCl_3$, 400 MHz): δ 7.27 (1H), 7.11 (1H), 6.77 (1H), 4.60 (2H), 3.70 (1H), 3.63 (1H), 3.22 (2H), 2.80 (2H), 2.53 (2H).

Product **5a**:

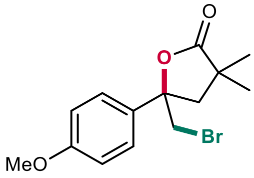

^{1}H NMR, ($CDCl_3$, 400 MHz): δ 7.34 (2H), 6.90 (2H), 3.82 (3H), 3.68 (1H), 3.56 (1H), 2.72 (1H), 2.47 (1H), 1.36 (3H), 0.99 (3H).

Product **6a**:

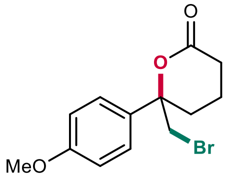

^{1}H NMR, ($CDCl_3$, 400 MHz): δ 7.30 (1H), 6.91 (1H), 3.82 (3H), 3.65 (1H), 3.58 (1H), 2.53-2.31 (4H), 1.88-1.80 (1H), 1.68-1.56 (1H).

Product **7a**:

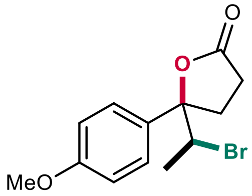

^{1}H NMR, ($CDCl_3$, 600 MHz), major isomer: δ 7.24 (2H), 6.88 (2H), 4.38 (1H), 3.80 (3H), 2.92-2.75 (1H), 2.71-2.62 (1H), 2.53-2.44 (2H), 1.50 (3H).

Product **8**a:

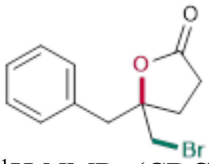

^{1}H NMR, ($CDCl_3$, 400 MHz): δ 7.30 (5H), 3.54-3.47 (1H), 3.14 (1H), 3.03 (1H), 2.54-2.46 (1H), 2.35-2.17 (2H), 2.09-2.00 (1H).

VII. Calibration Curves

Calibration curve of substrate **1** using *p*-bromoanisole as internal standard:

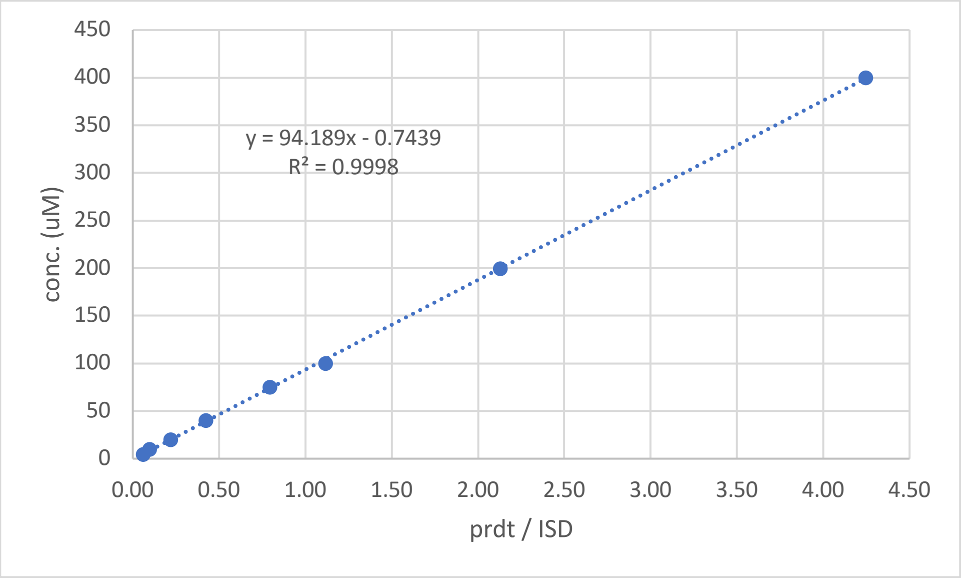

Calibration curve of substrate **2** using *p*-bromoanisole as internal standard:

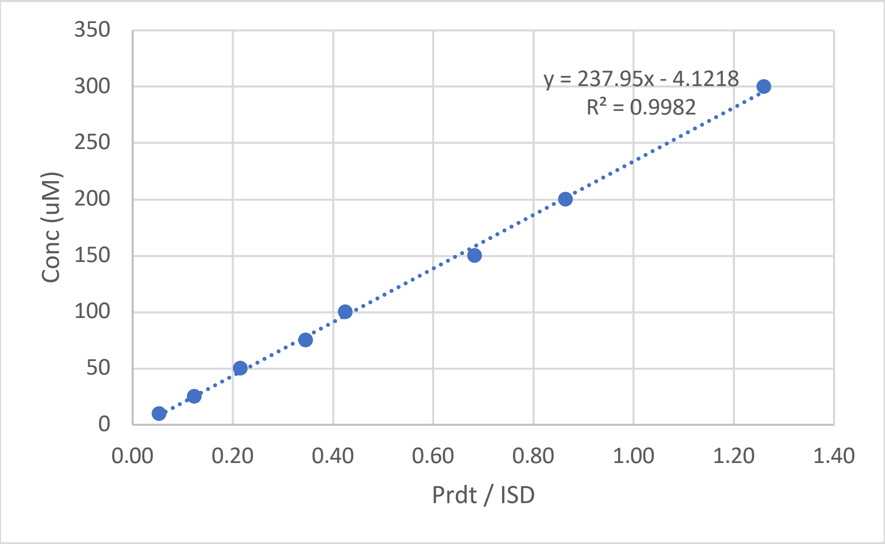

Calibration curve of substrate **3** using *p*-bromoanisole as internal standard:

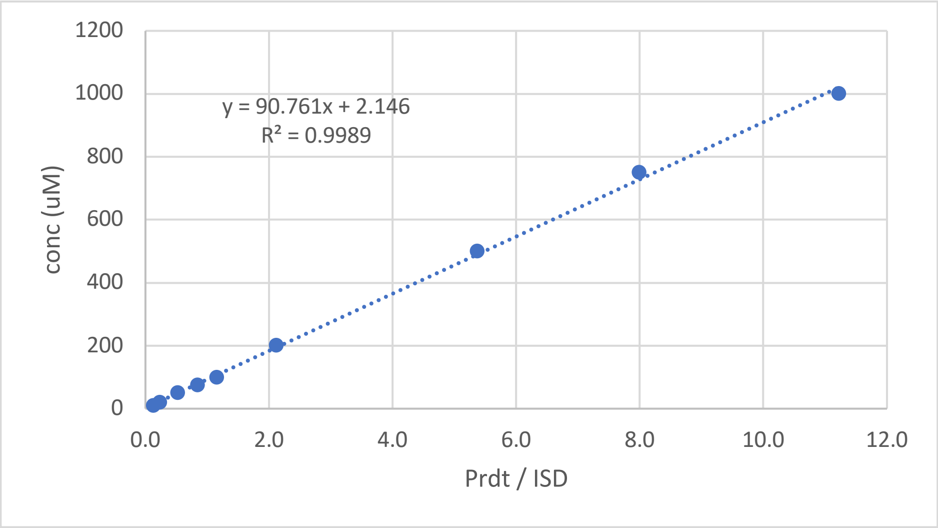

Calibration curve of substrate **4** using *p*-bromoanisole as internal standard:

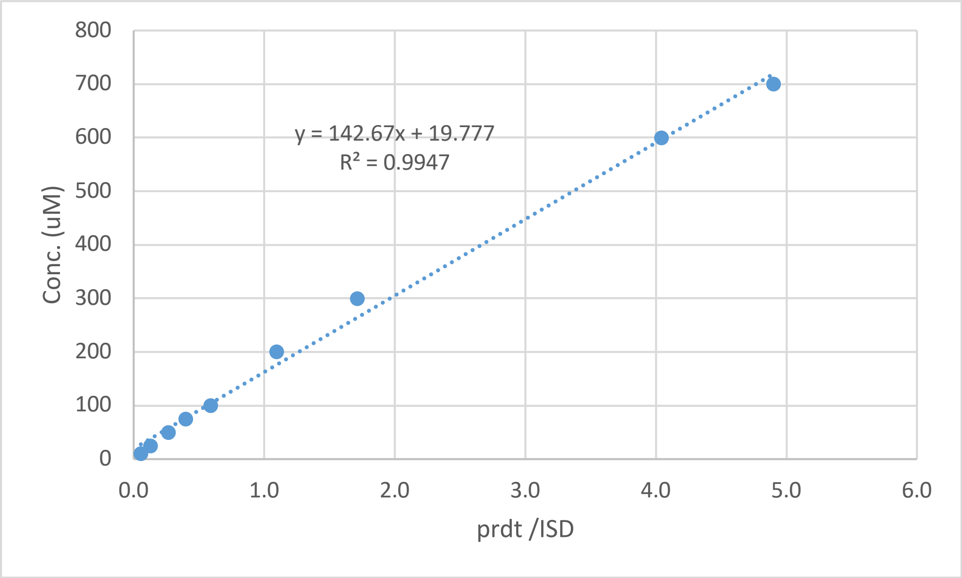

Calibration curve of substrate **5** using *p*-chloroanisole as internal standard:

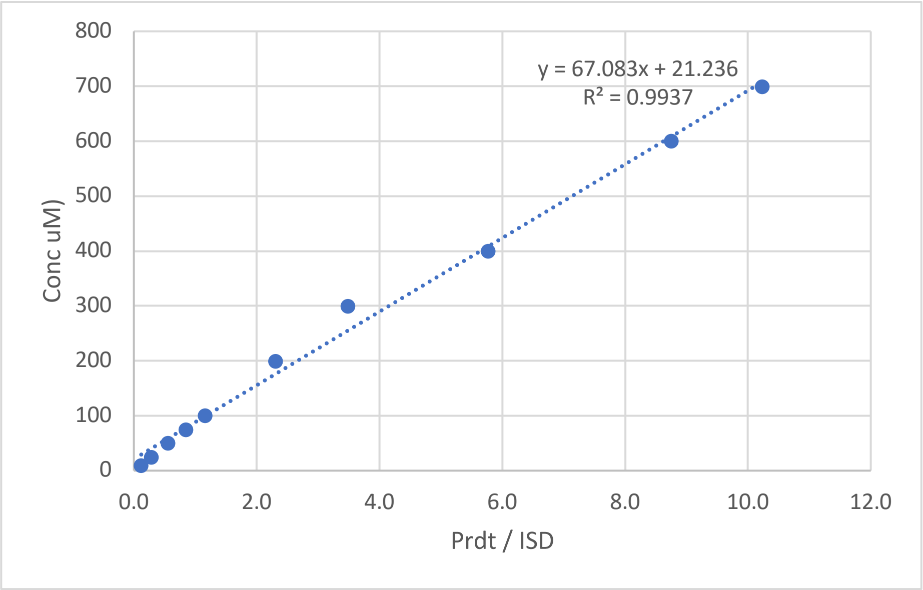

Calibration curve of substrate **6** using *p*-chloroanisole as internal standard:

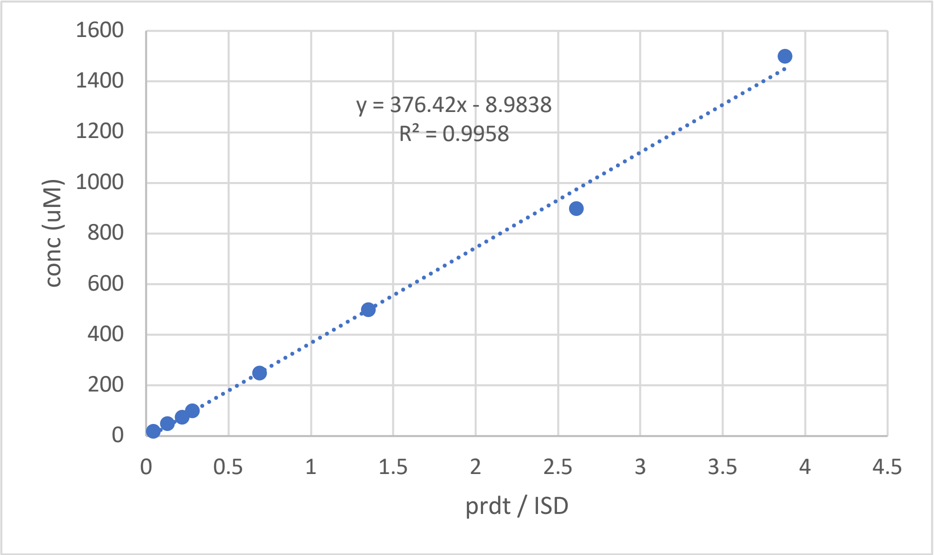

Calibration curve of substrate **7** using *p*-bromoanisole as internal standard:

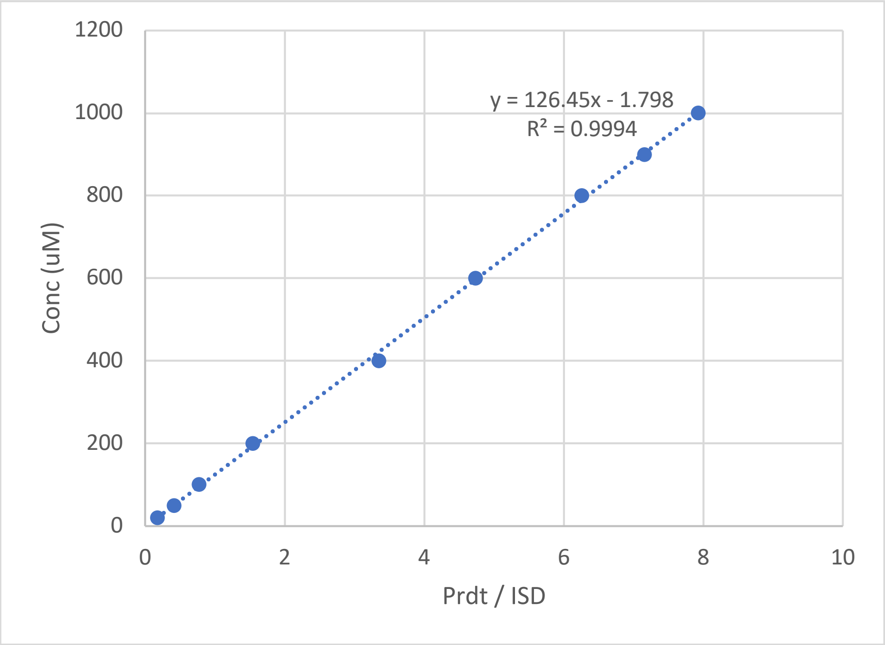

Calibration curve of substrate **8** using *p*-bromoanisole as internal standard:

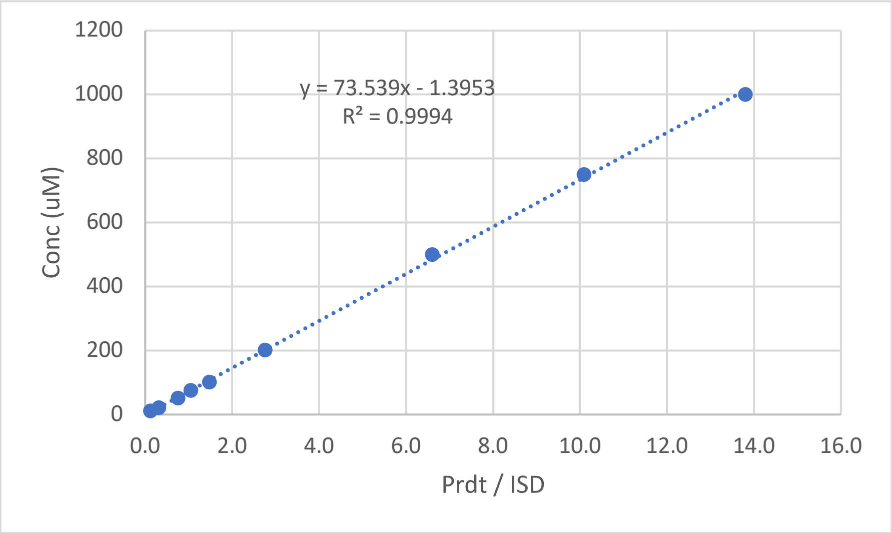

Calibration curve of substrate **1** using 2,6-dimethyl benzonitrile as internal standard:

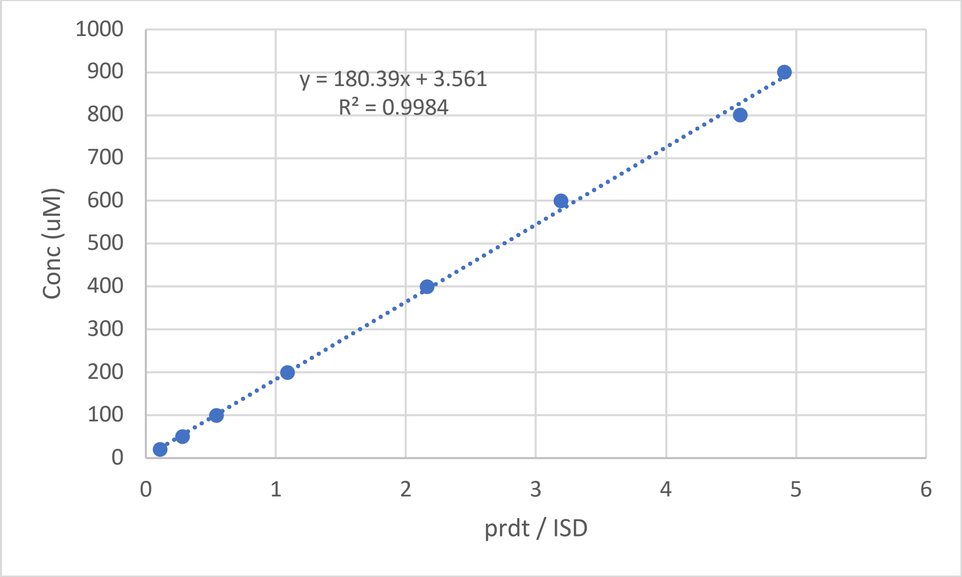

Calibration curve of substrate **1c** using 2,6-dimethyl benzonitrile as internal standard:

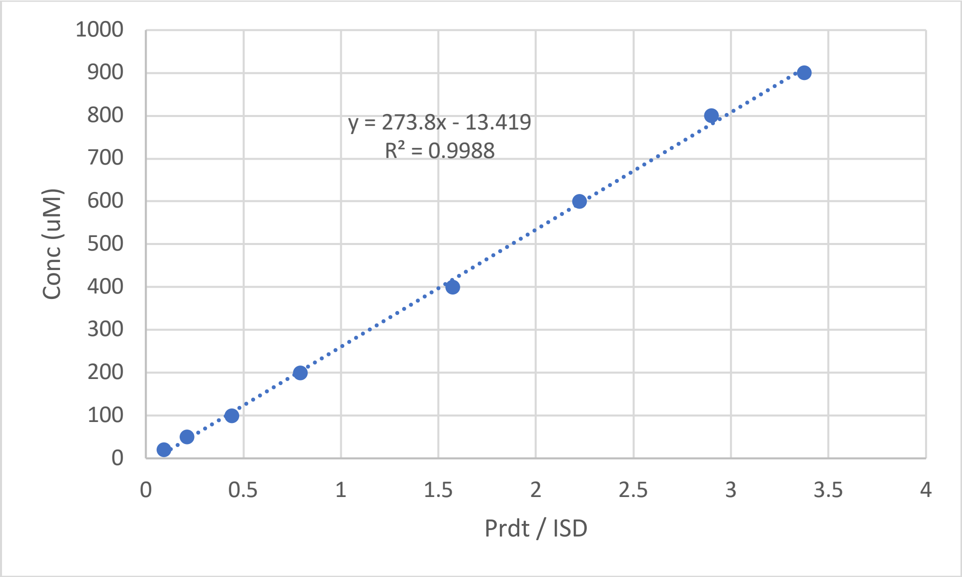

VIII. Chiral HPLC Analysis

Chiral Analysis of Product **1**:

Racemic

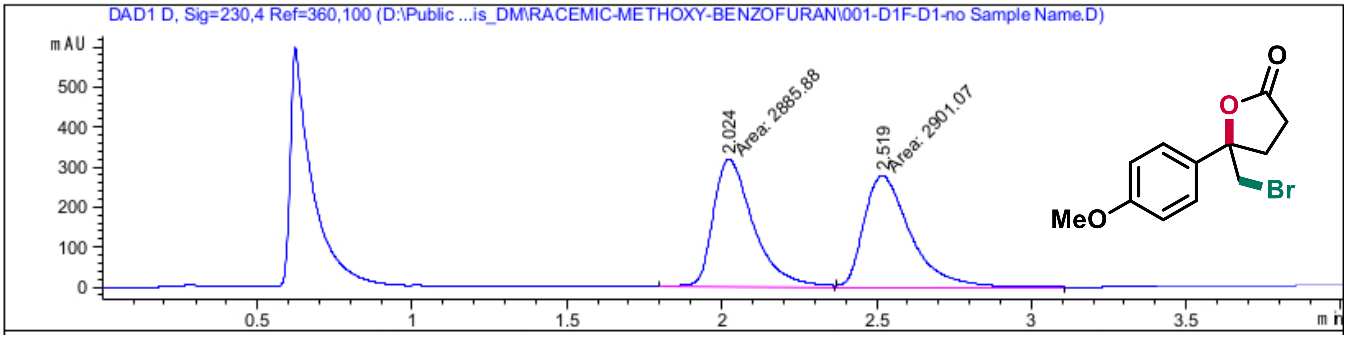

Bioconversion

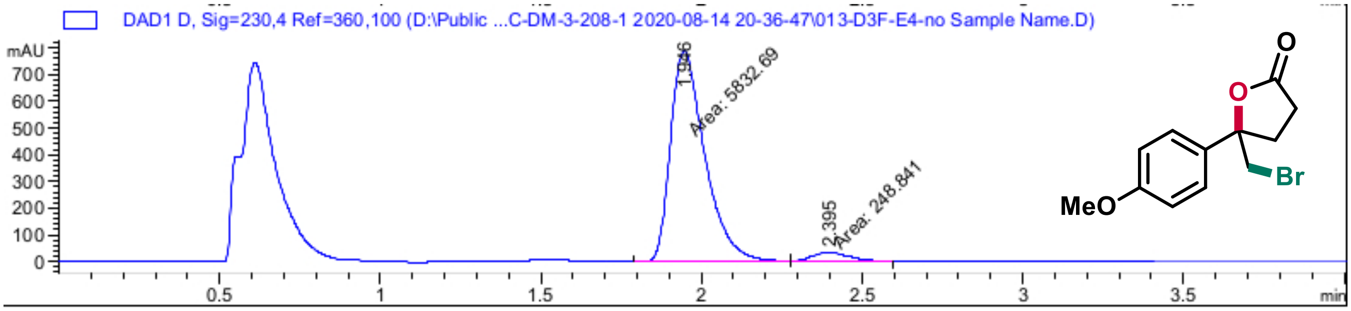

Chiral Analysis of Product **2**:

racemic

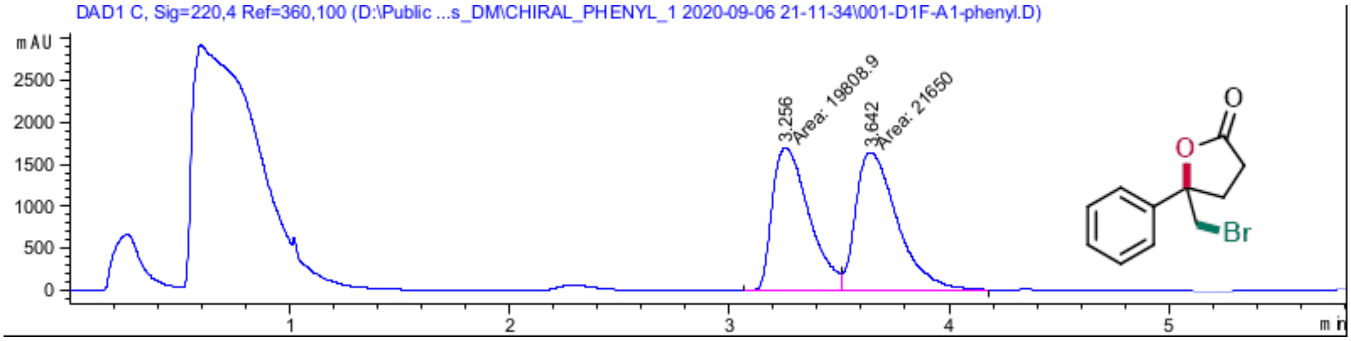

bioconversion

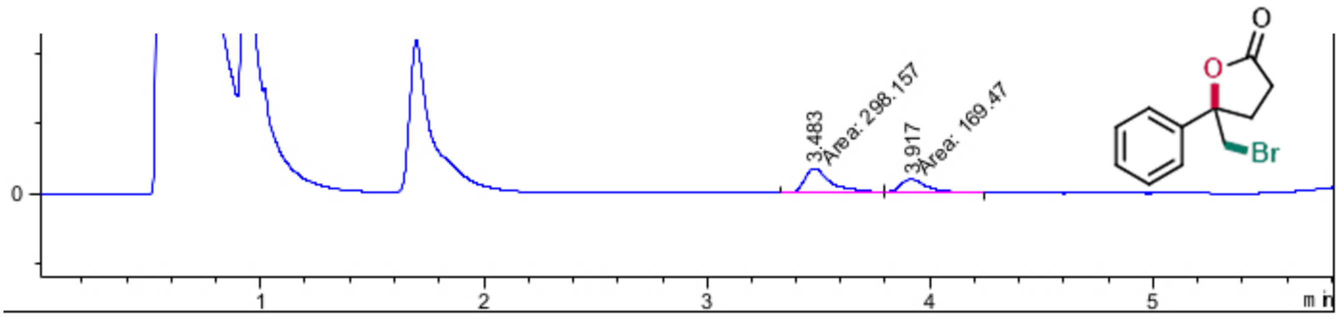

Chiral Analysis of Product **3**:

racemic

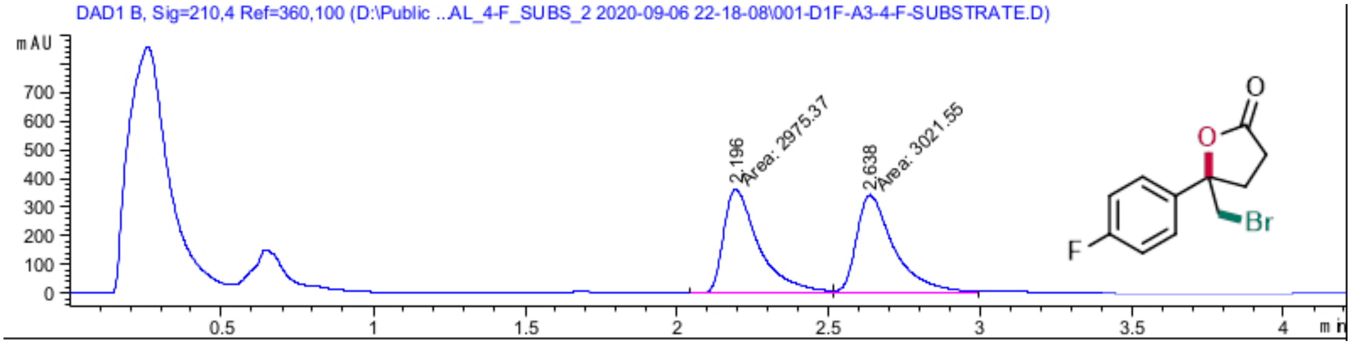

bioconversion

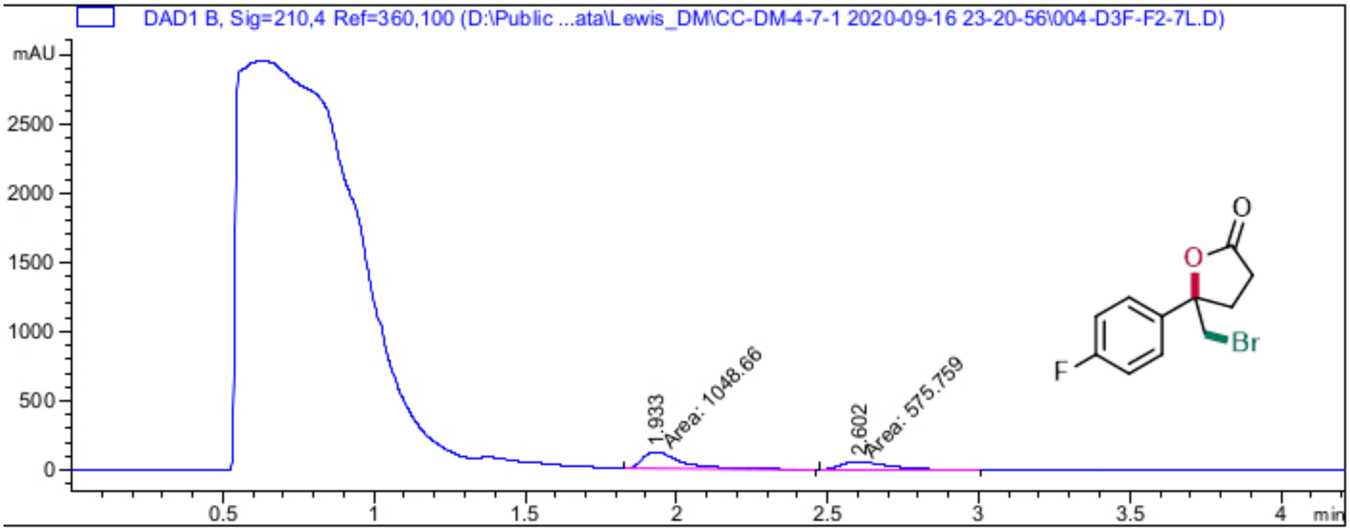

Chiral Analysis of Product **4**:

racemic

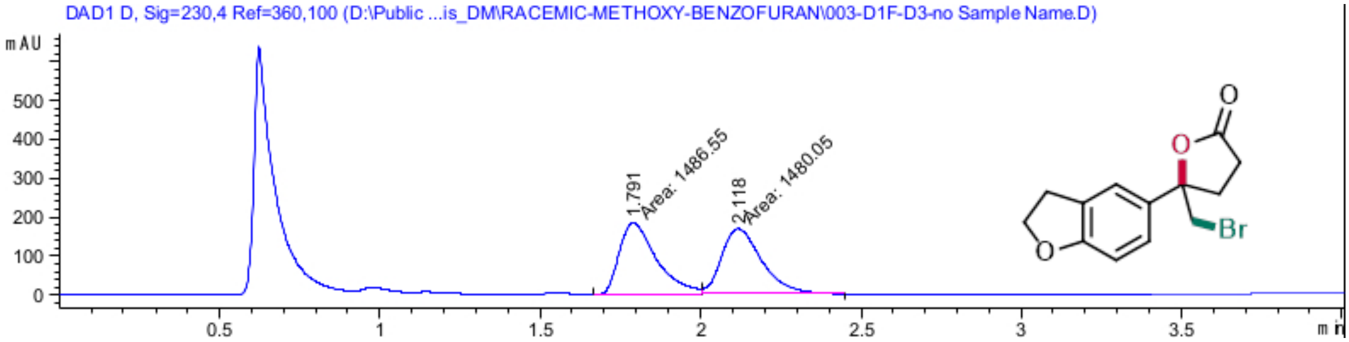

bioconversion

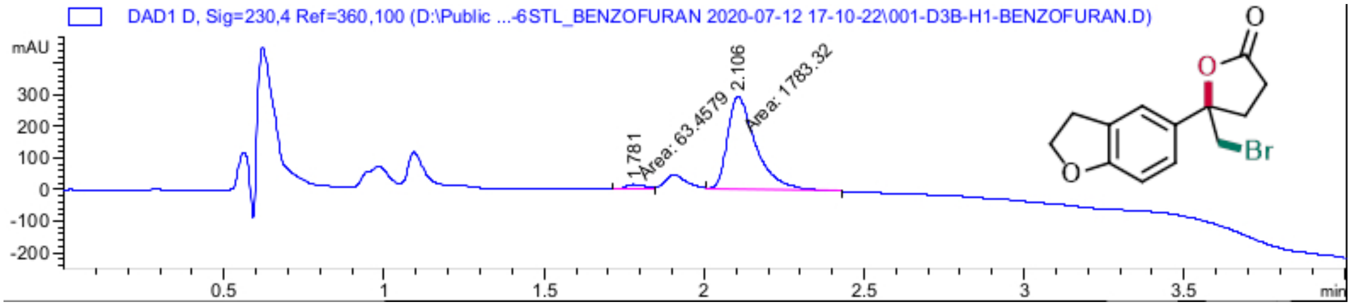

Chiral Analysis of Product **5**:

racemic

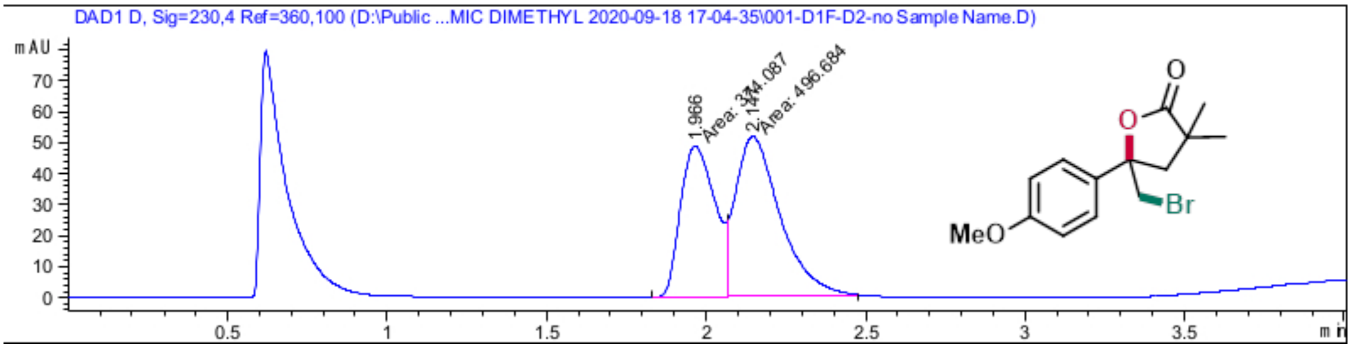

bioconversion

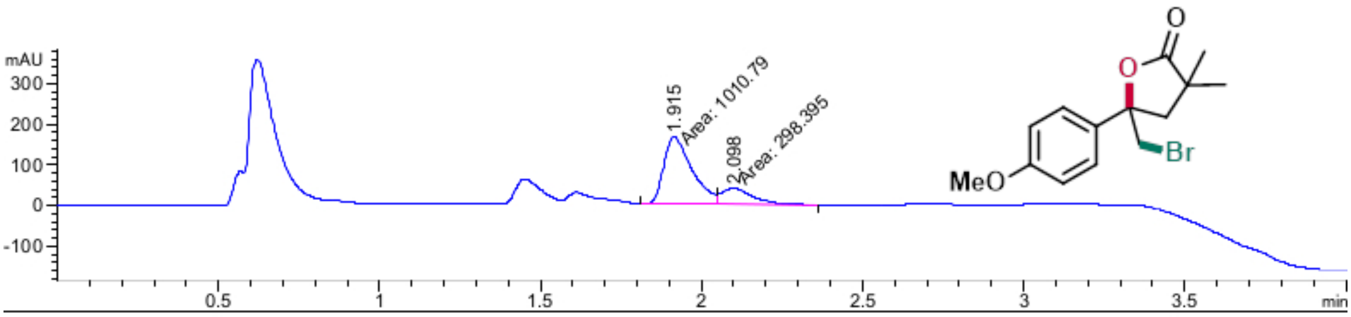

Chiral Analysis of Product **6**:

racemic

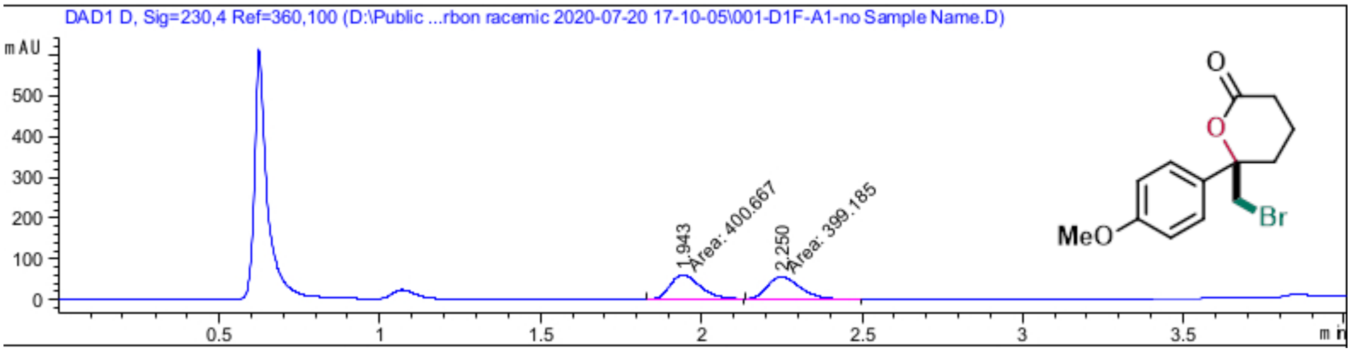

bioconversion

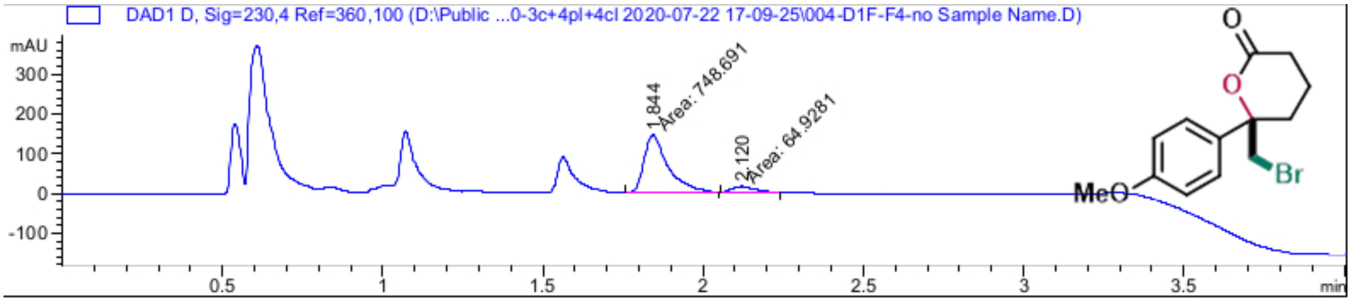

Chiral Analysis of Product **8**:

racemic

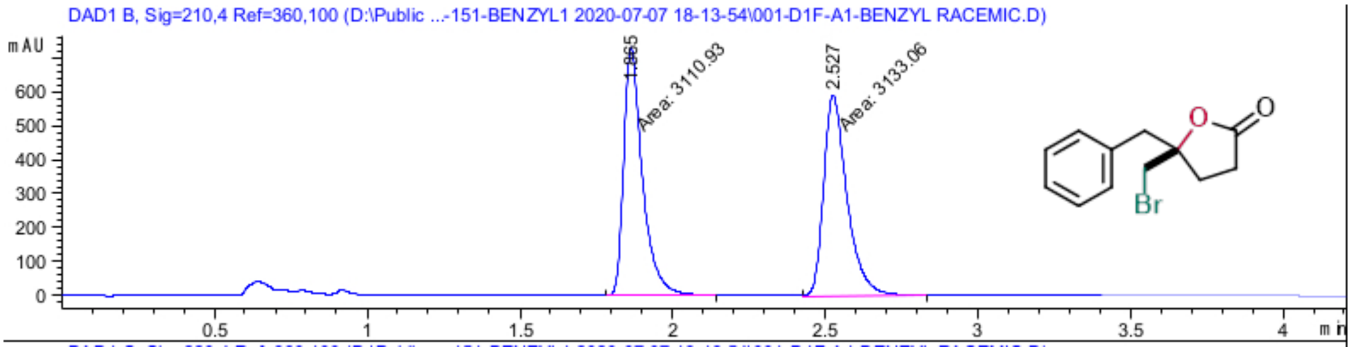

bioconversion

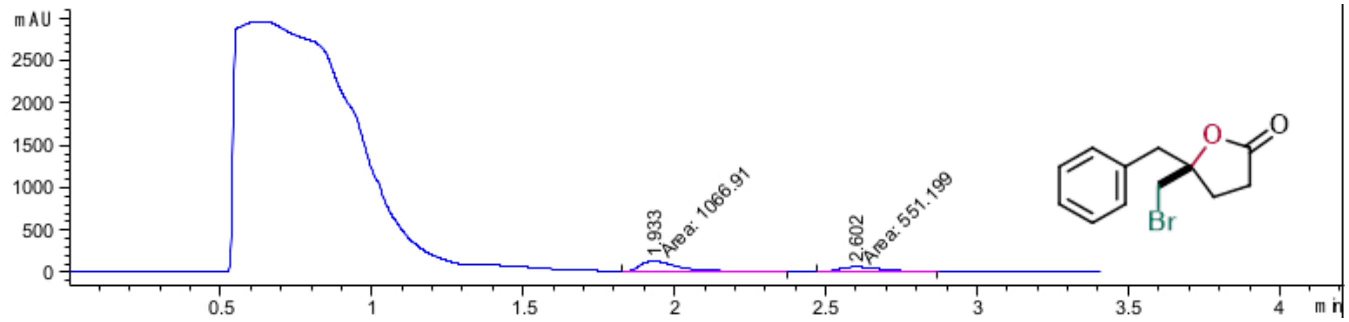

Chiral Analysis of Product **1C**:

Racemic

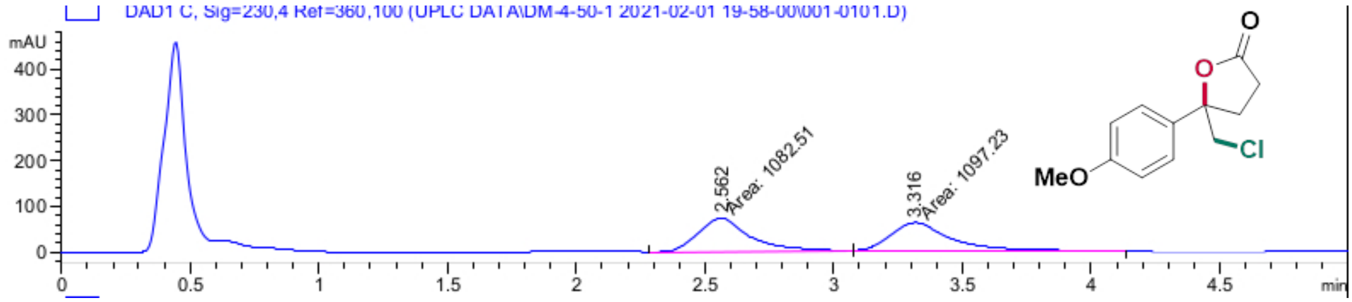

bioconversion

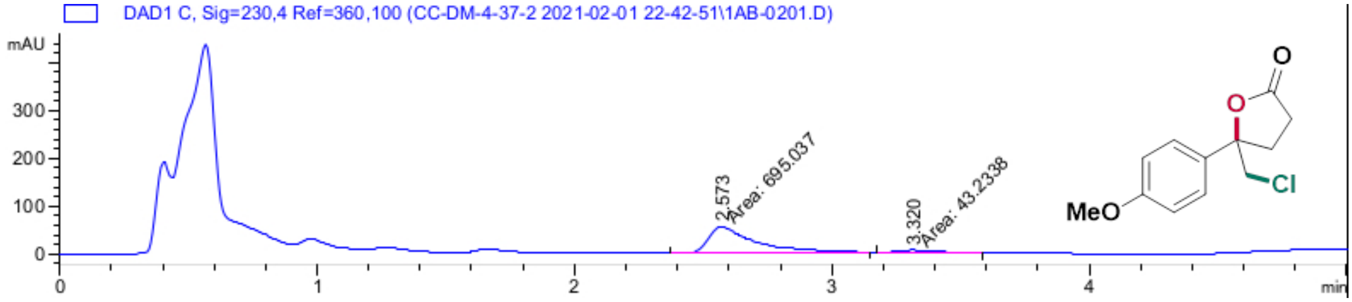

IX. NMR Spectra

Supplementary figure 16: ^{1}H NMR spectrum of Substrate **1**

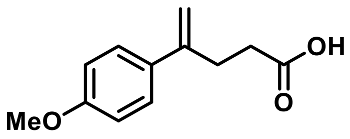

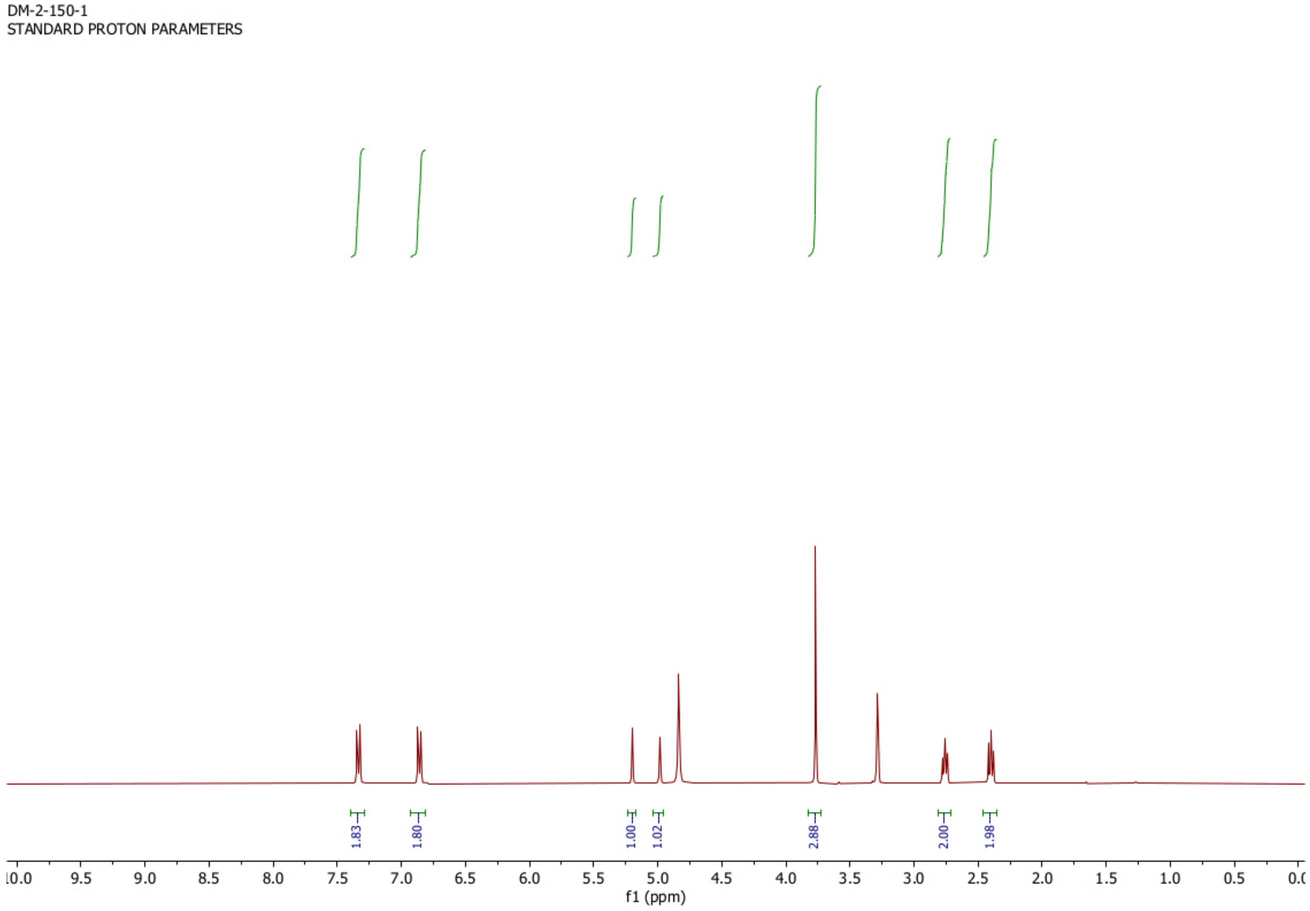

Supplementary figure 17: ^{1}H NMR spectrum of Substrate **2.**

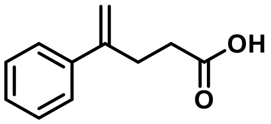

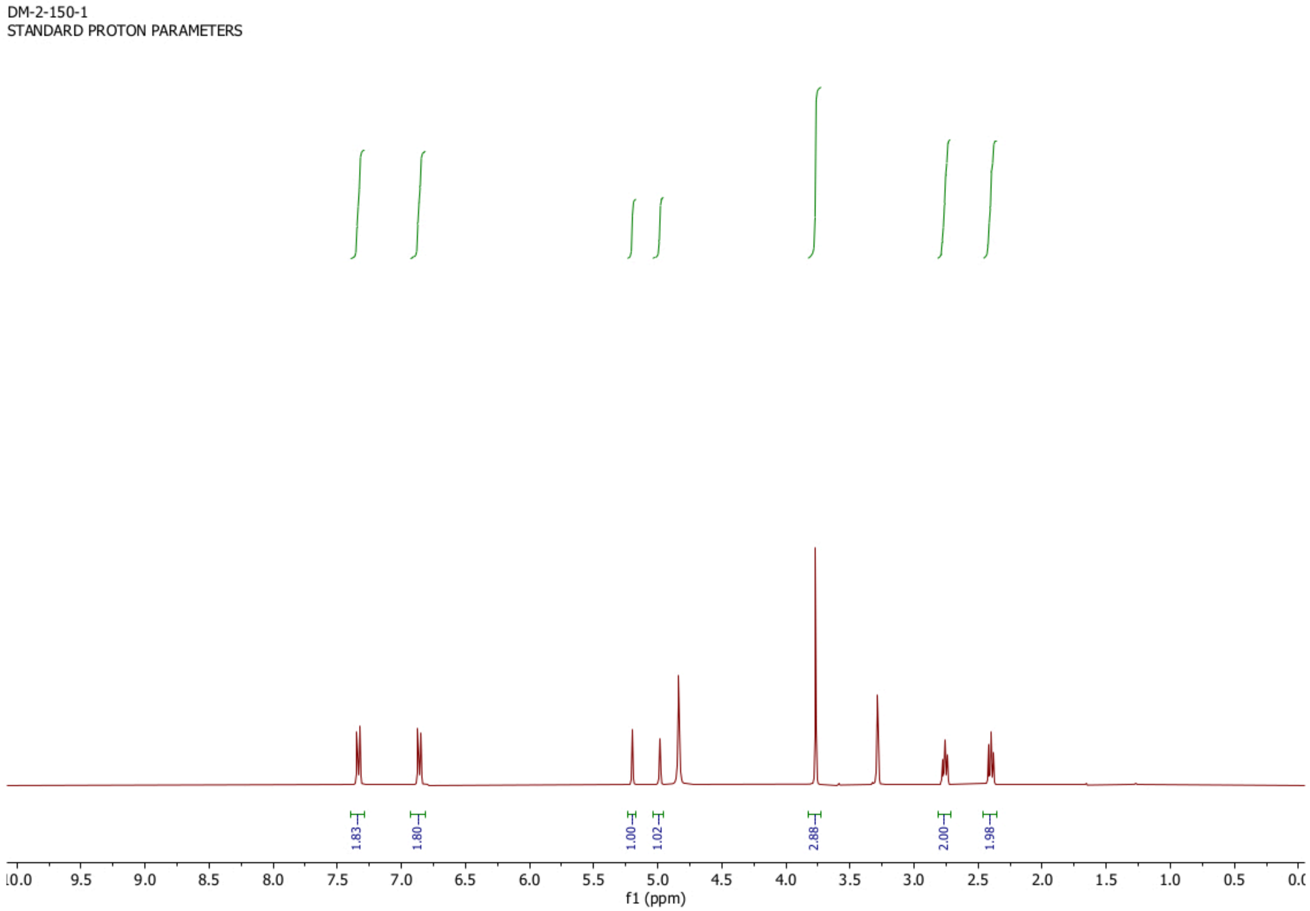

Supplementary figure 18: ^{1}H NMR spectrum of Substrate **3.**

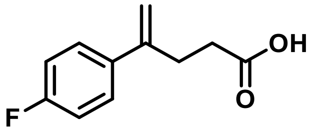

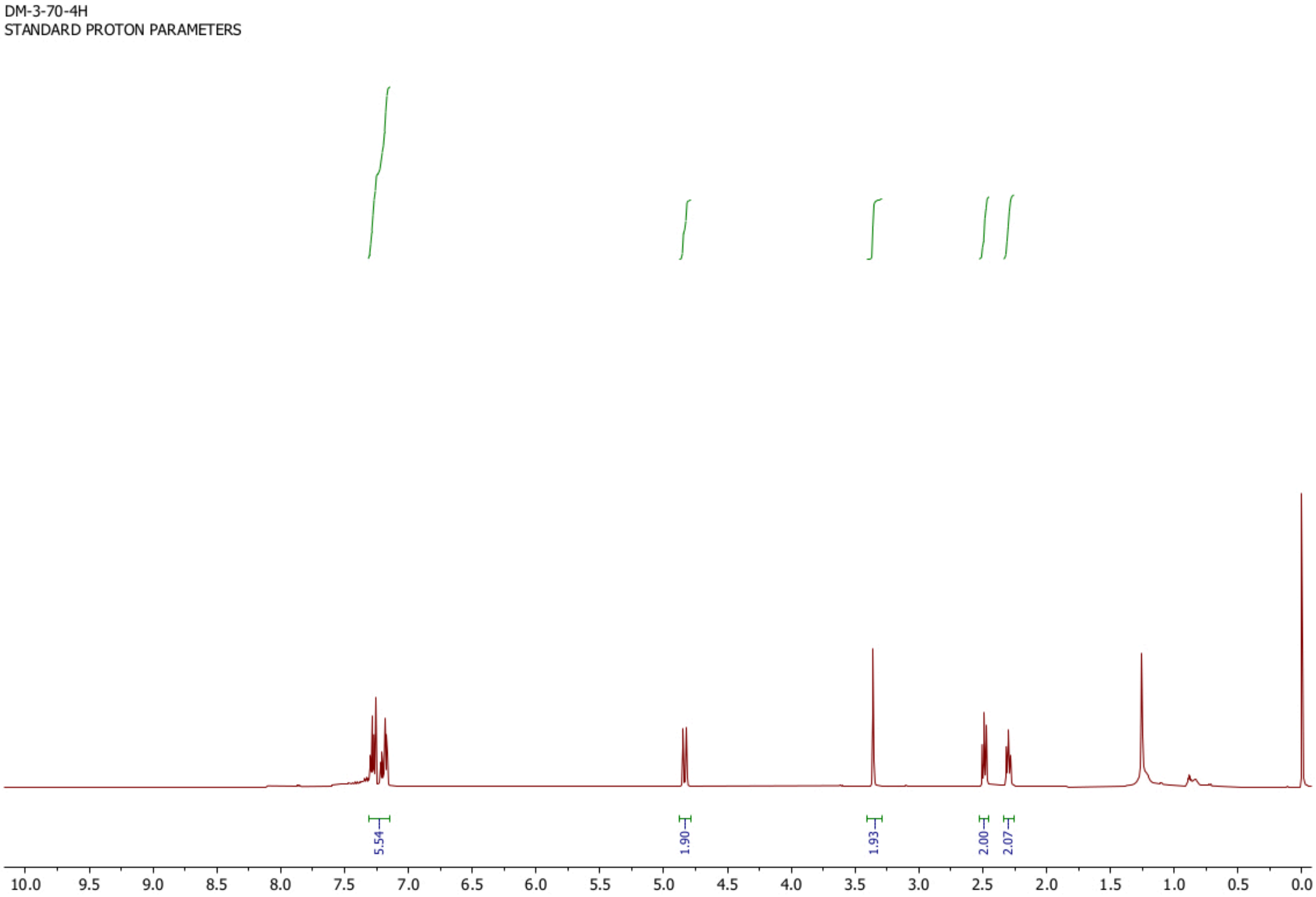

Supplementary figure 19: ^{1}H NMR spectrum of Substrate **4.**

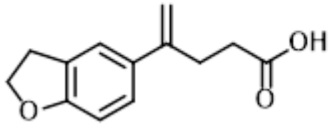

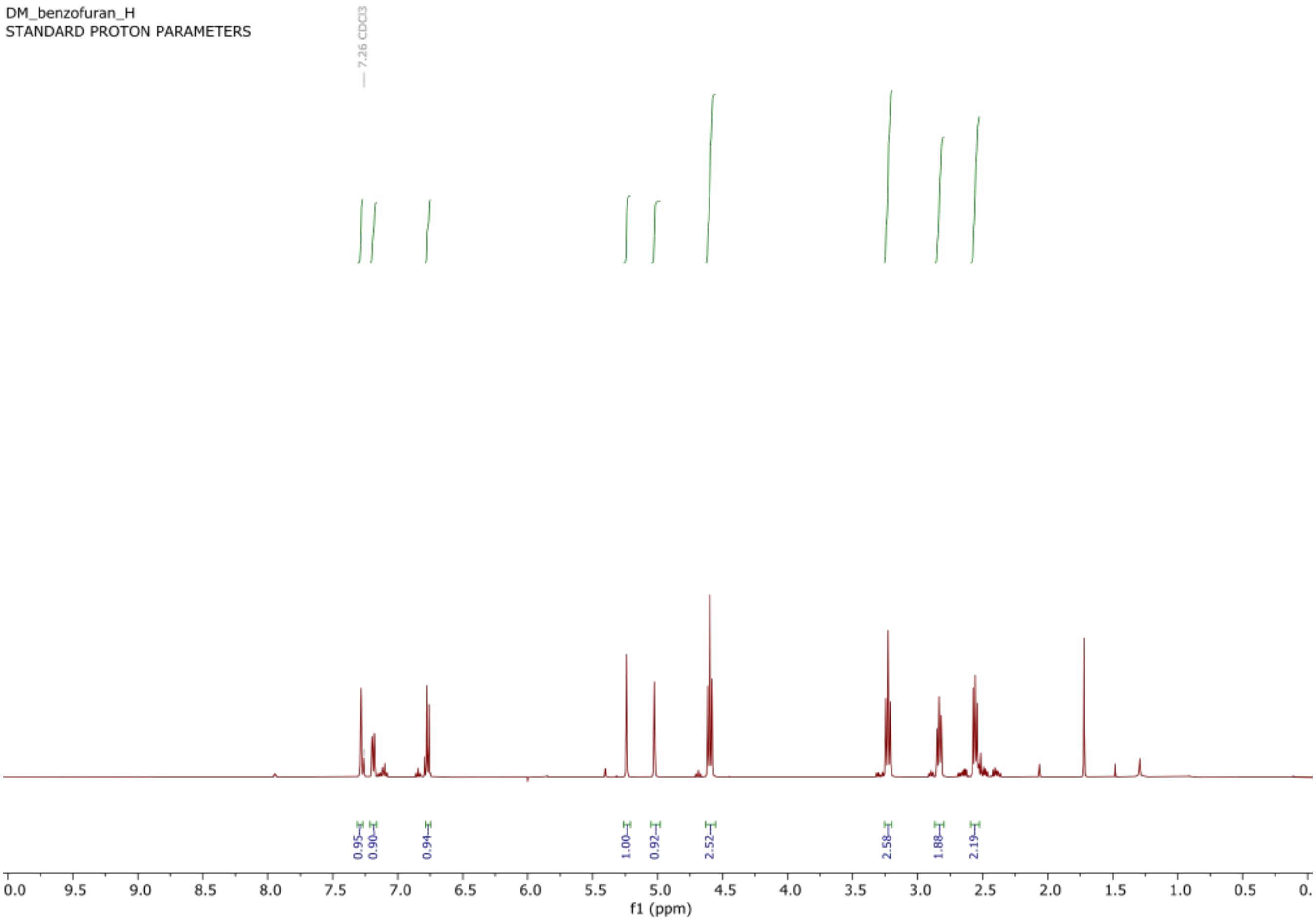

Supplementary figure 20: ^{13}C NMR spectrum of Substrate **4**.

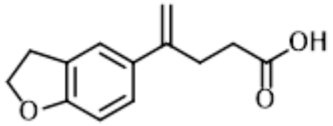

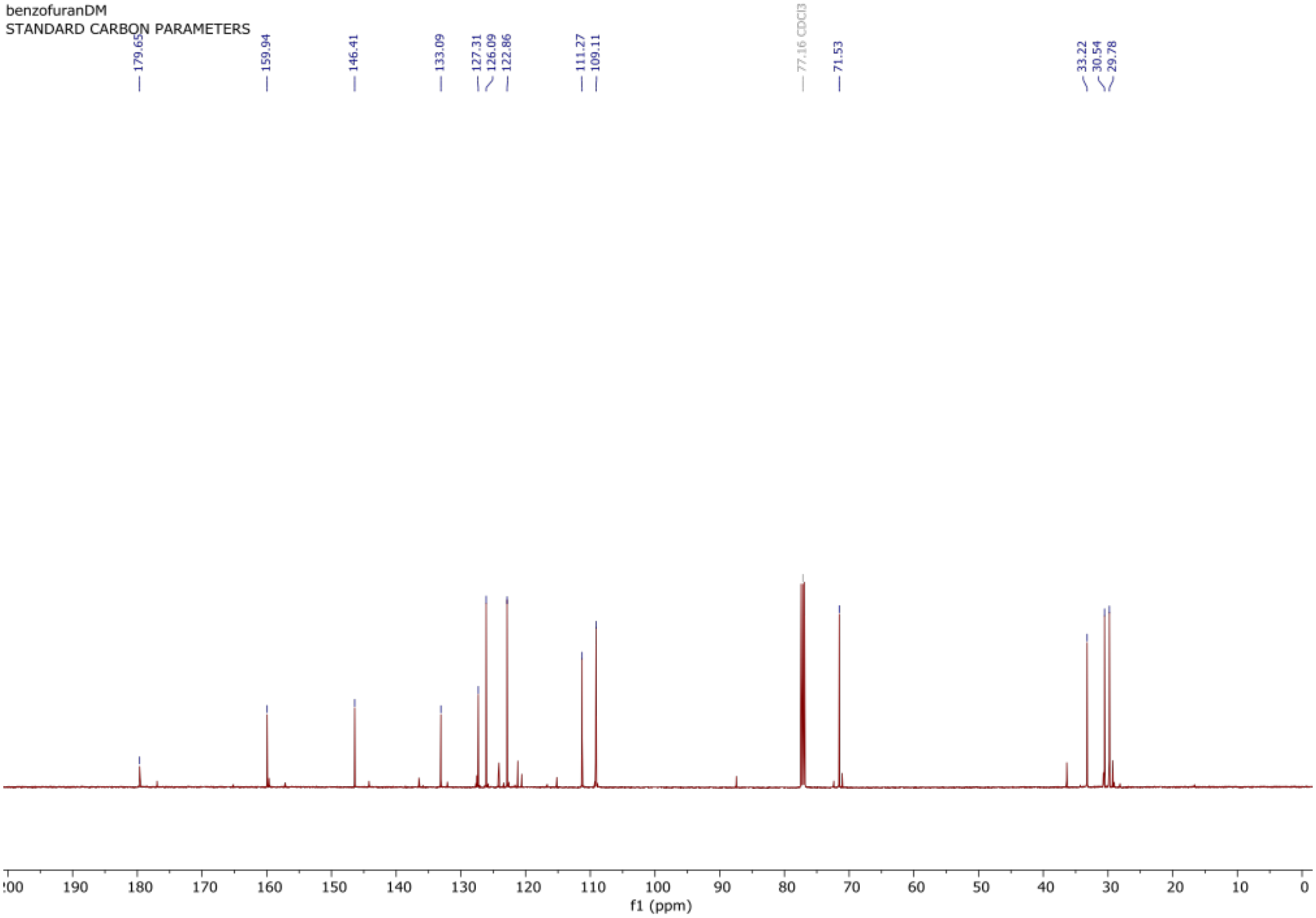

Supplementary figure 21: ^{1}H NMR spectrum of Substrate **5.**

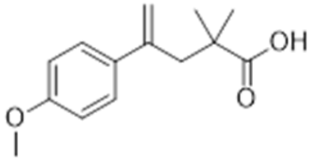

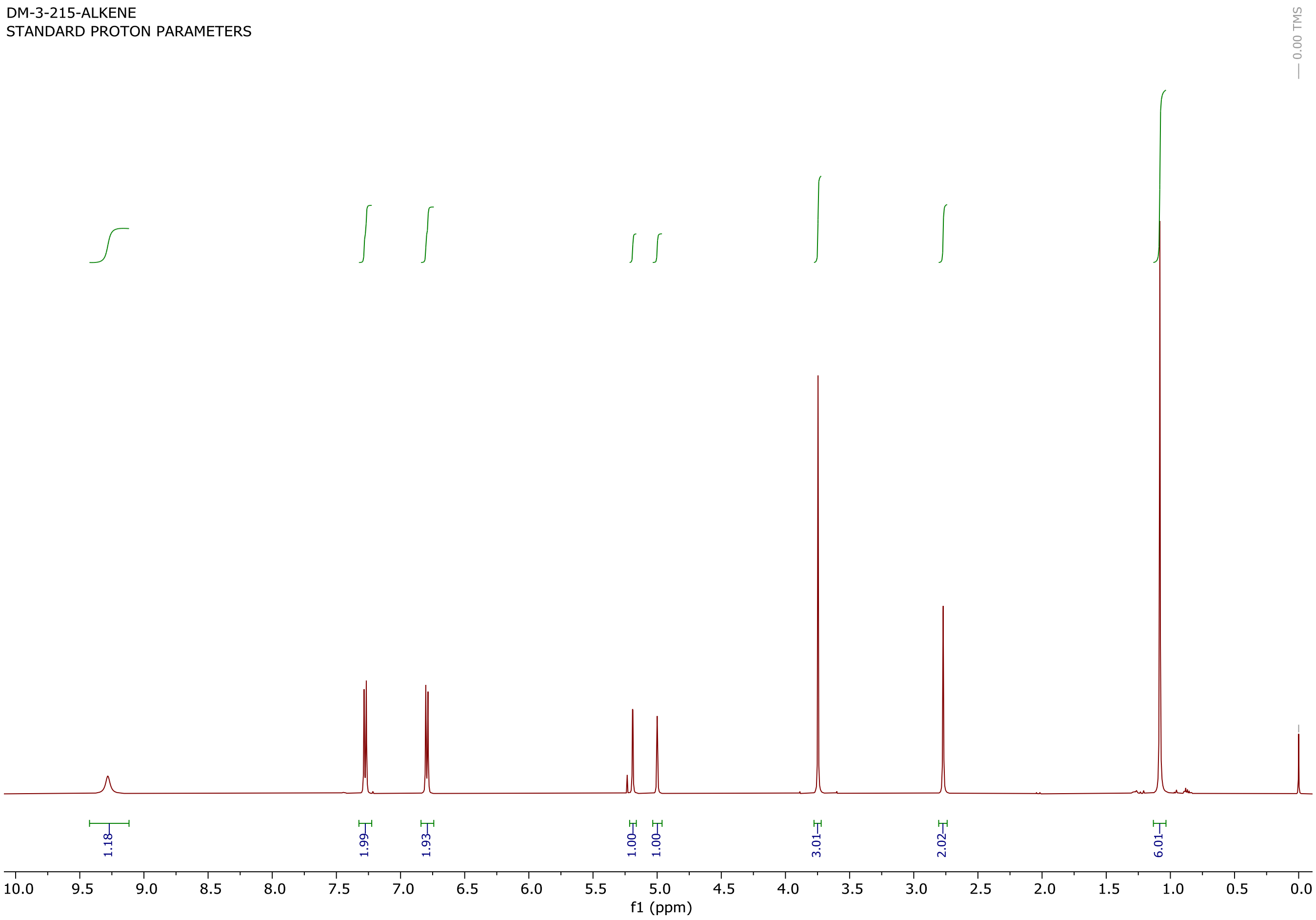

Supplementary figure 22: ^{13}C NMR spectrum of Substrate **5.**

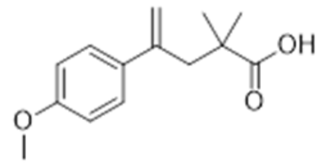

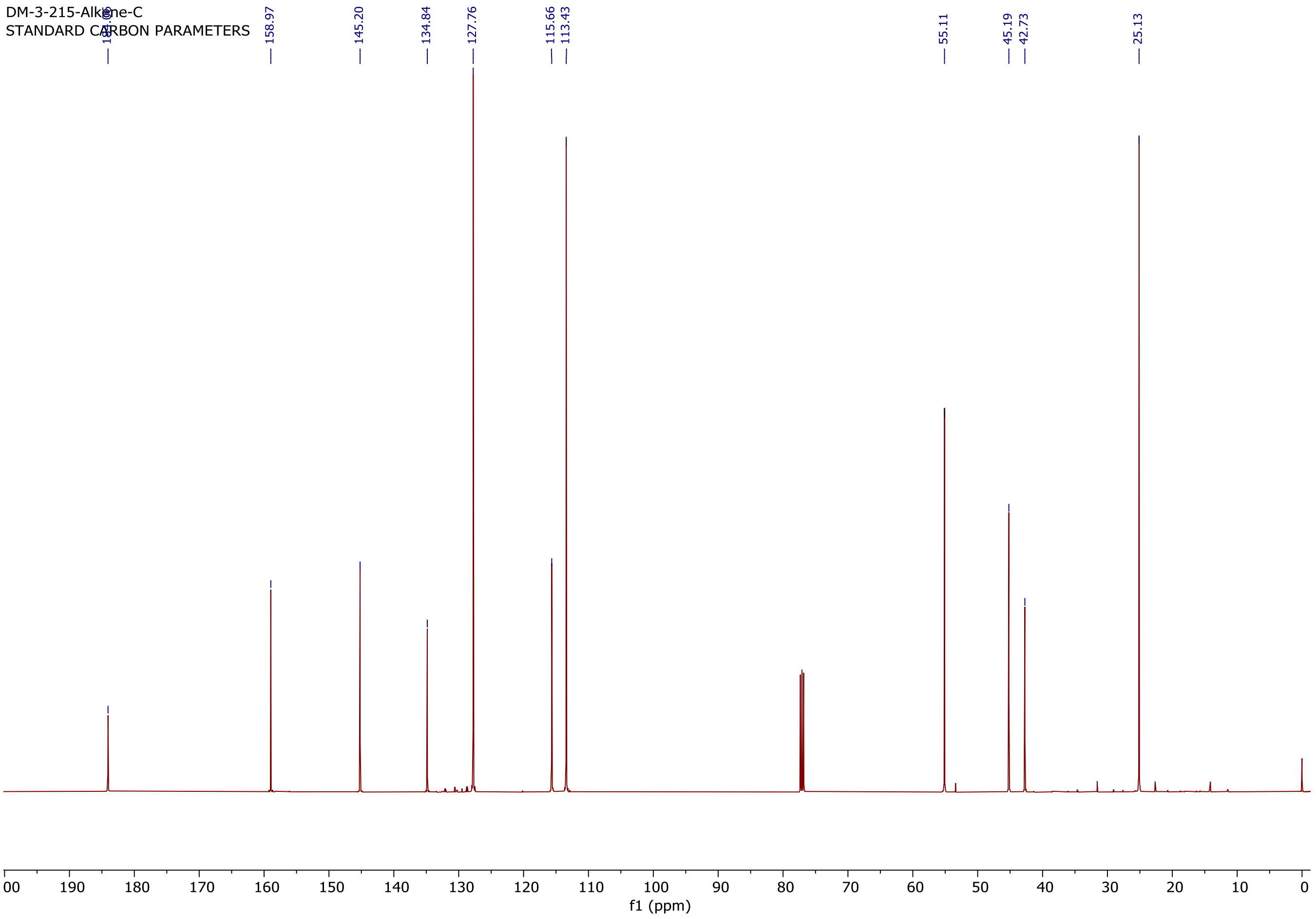

Supplementary figure 23: ^{1}H NMR spectrum of Substrate **6.**

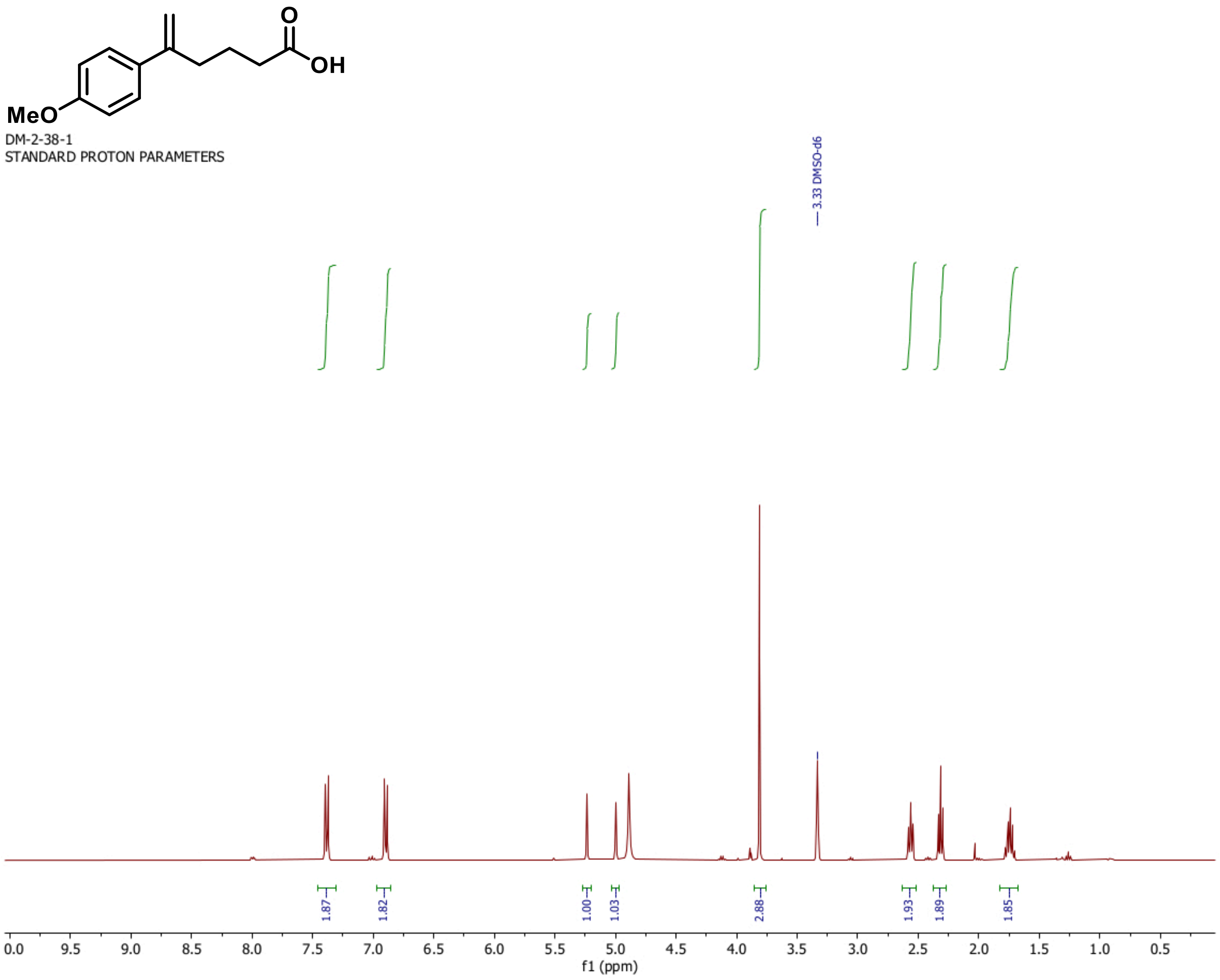

Supplementary figure 24: ^{1}H NMR spectrum of Substrate **7.**

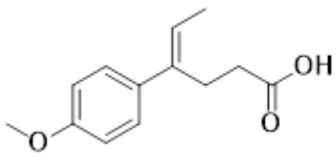

YJ-0906-E-acid
STANDARD PROTON PARAMETERS

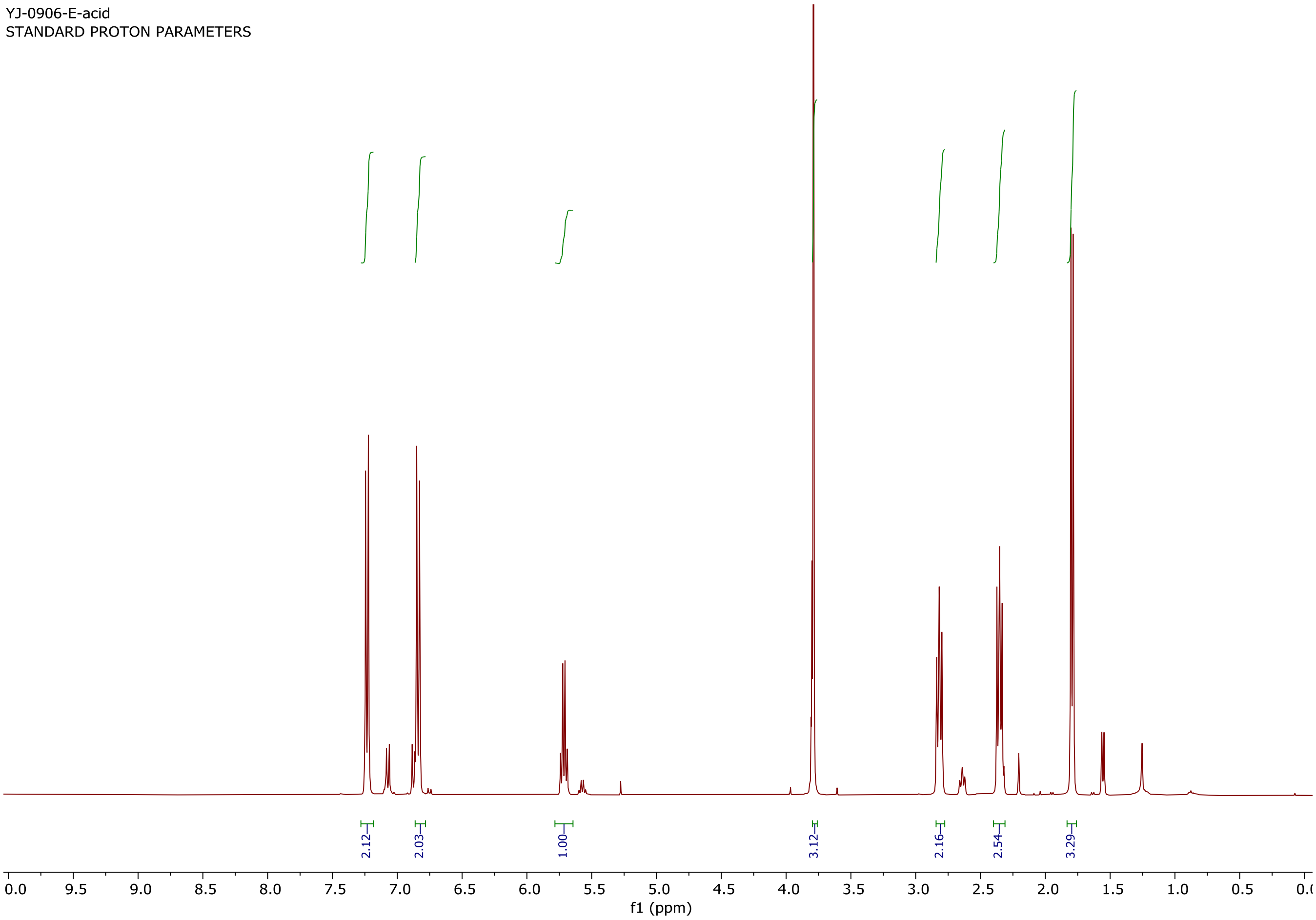

Supplementary figure 25: ^{13}C NMR spectrum of Substrate **7.**

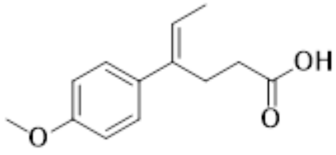

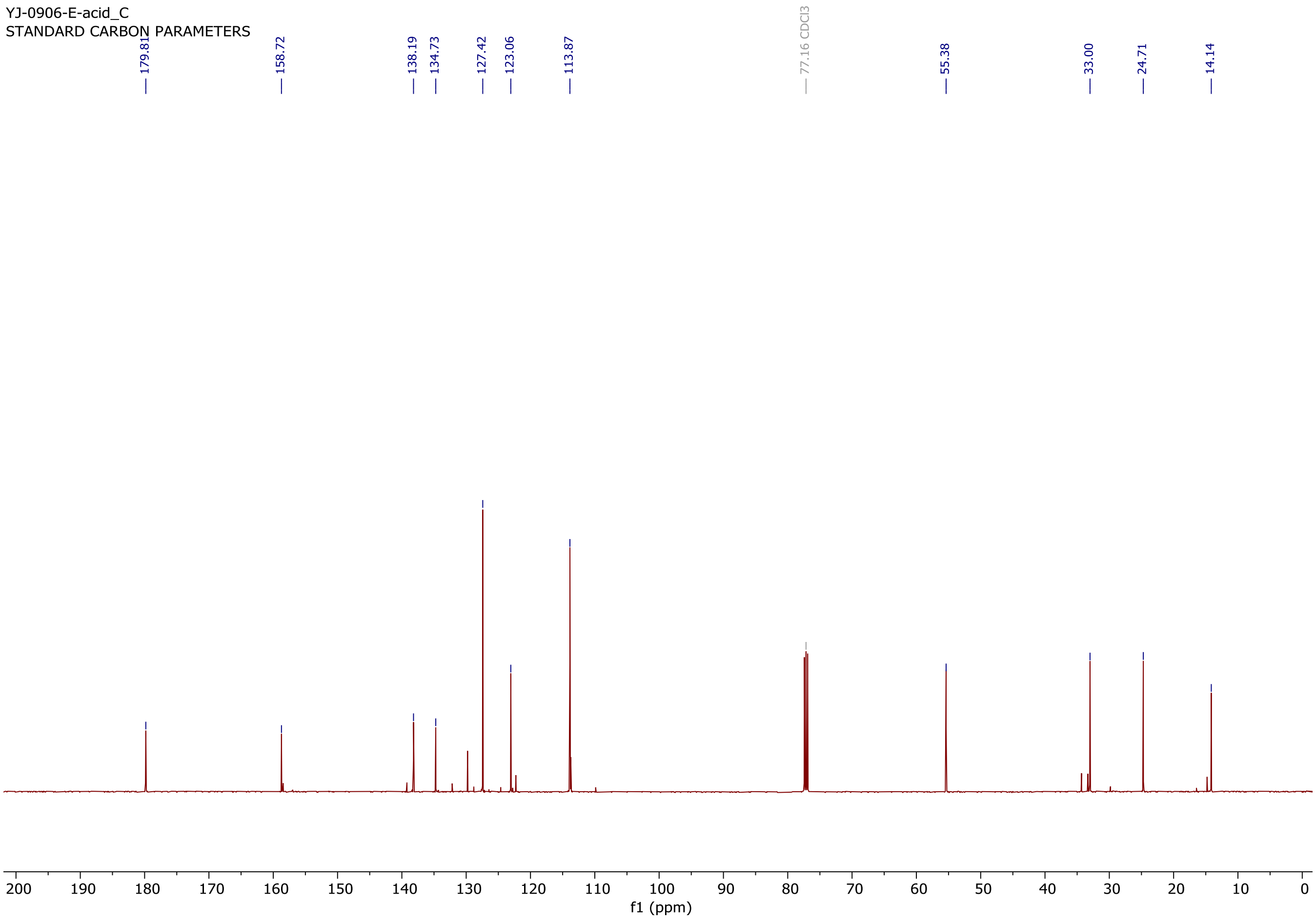

Supplementary figure 26: ^{1}H NMR spectrum of Substrate **8.**

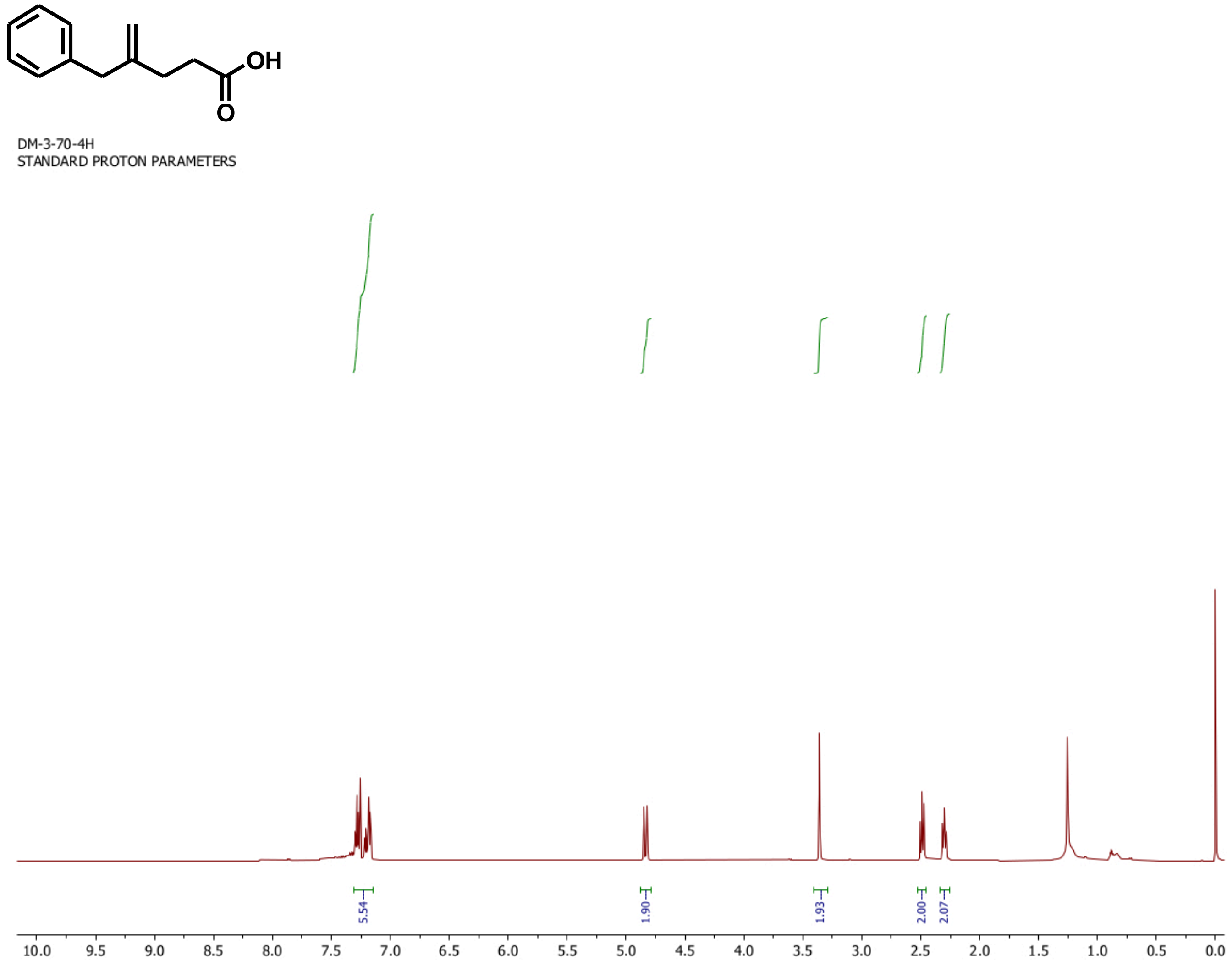

Supplementary figure 27: ^{1}H NMR spectrum of Product **1a**.

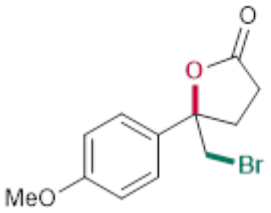

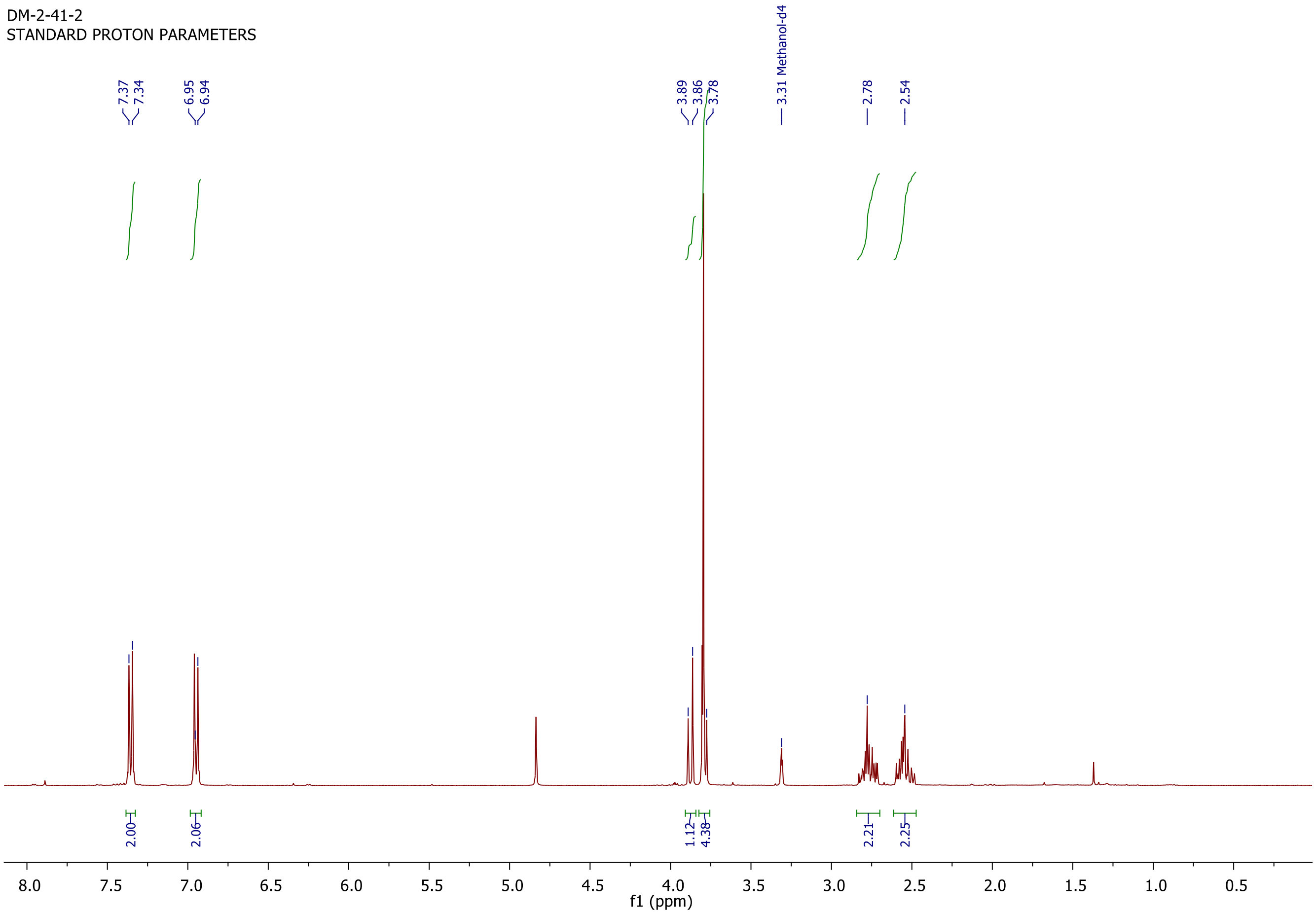

Supplementary figure 28: ^{1}H NMR spectrum of Product **1a** from preparative bioconversion:

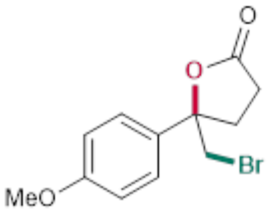

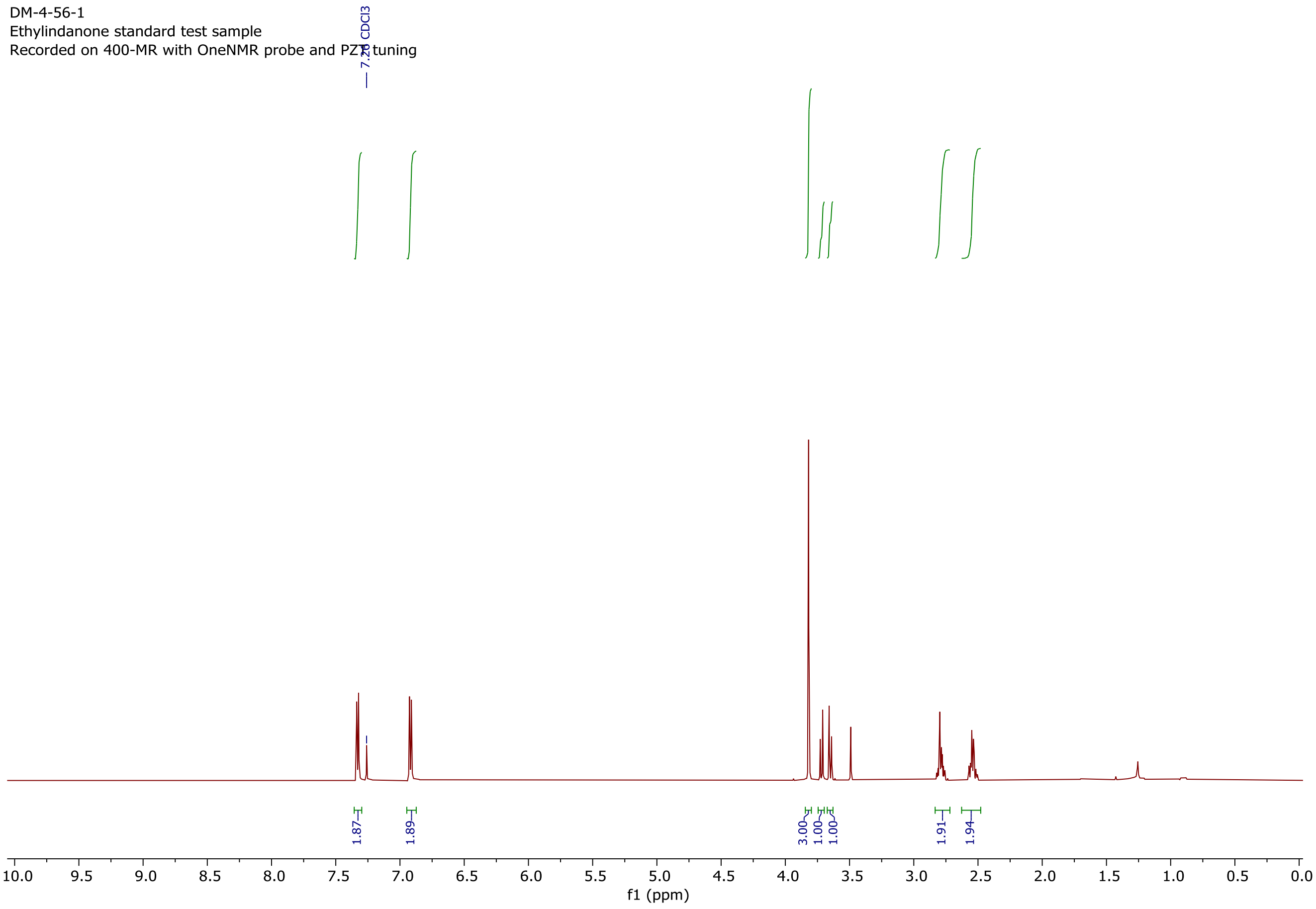

Supplementary figure 29: ^{1}H NMR spectrum of Product **1c**.

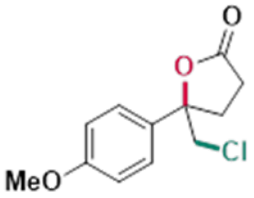

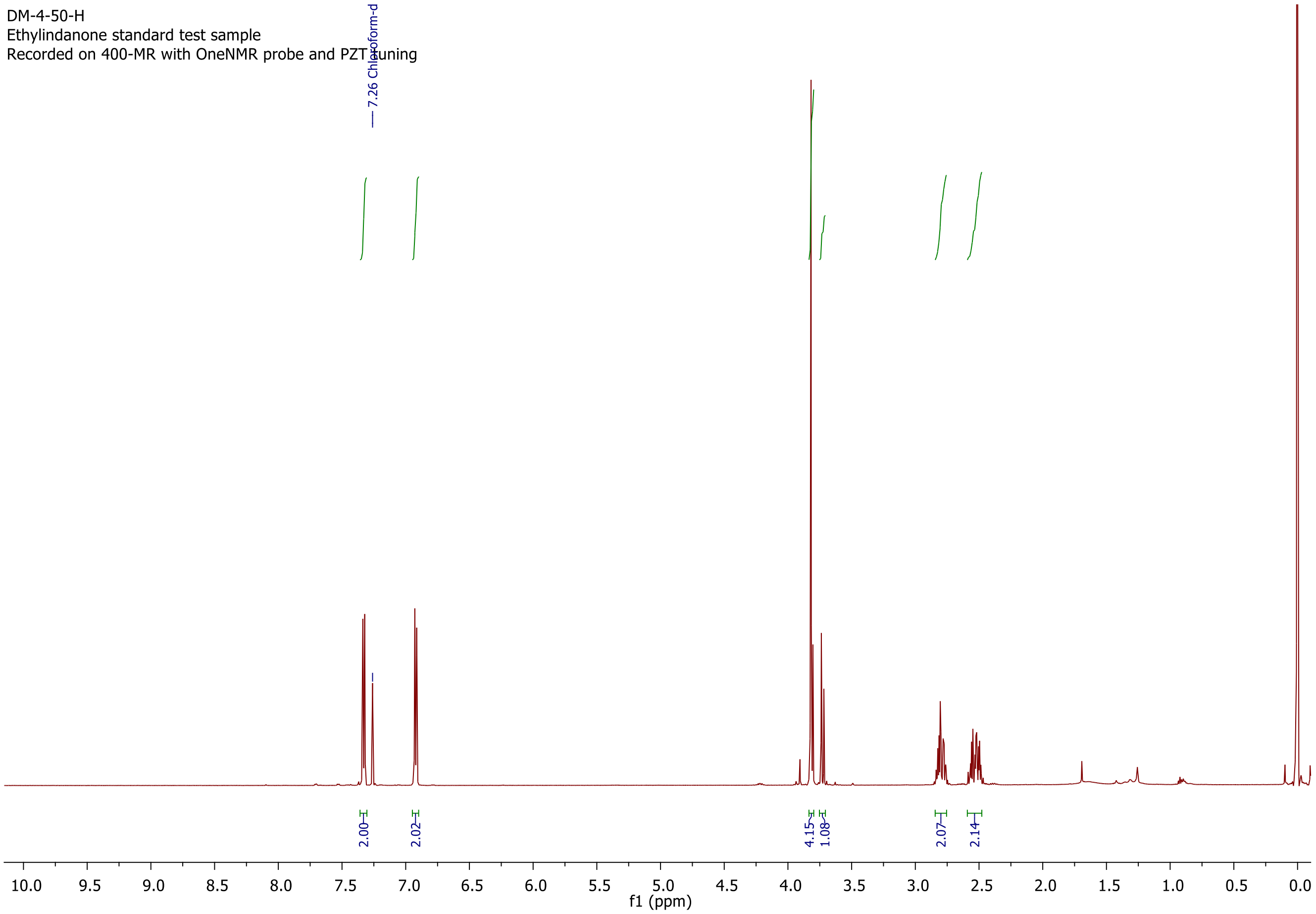

Supplementary figure 30: ^{1}H NMR spectrum of Product **2a**.

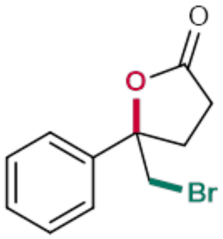

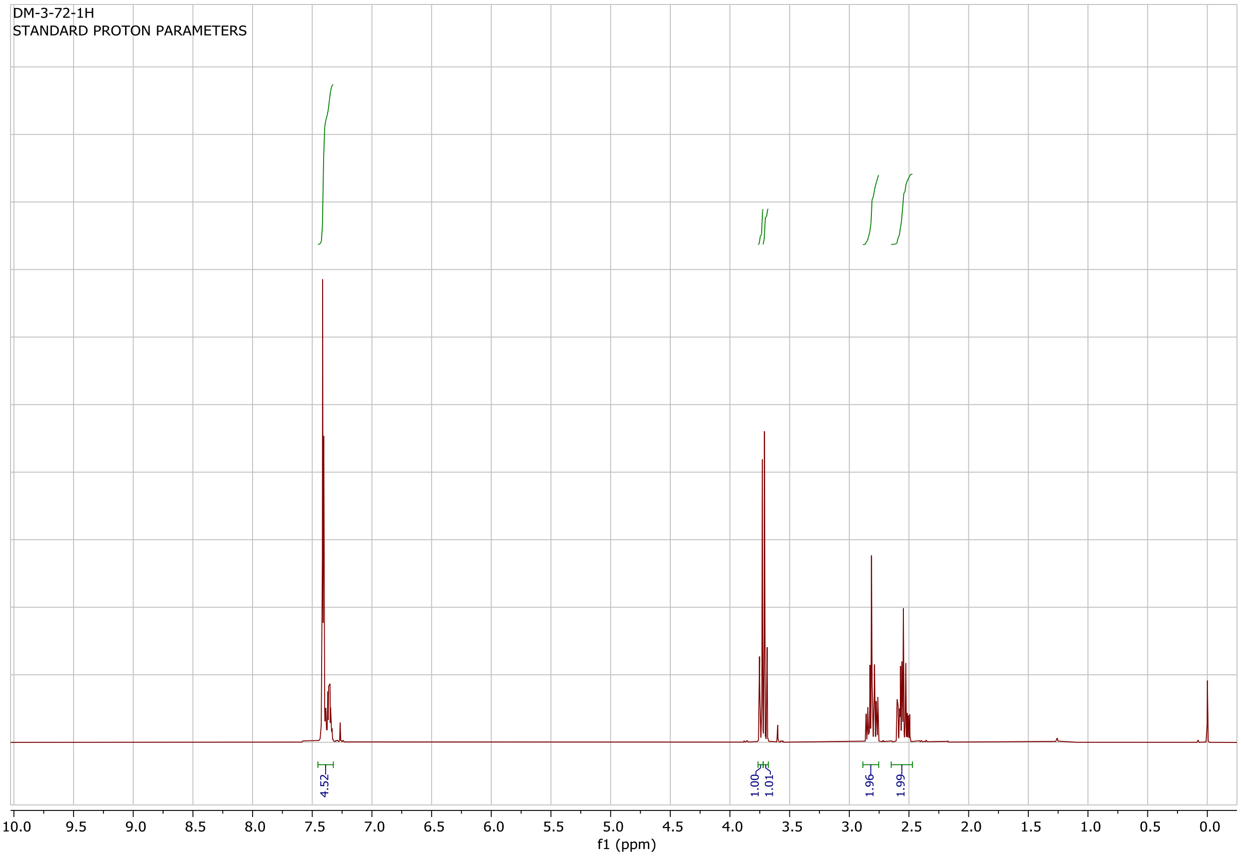

Supplementary figure 31: ^{1}H NMR spectrum of Product **3a**.

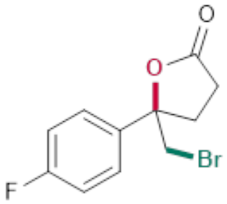

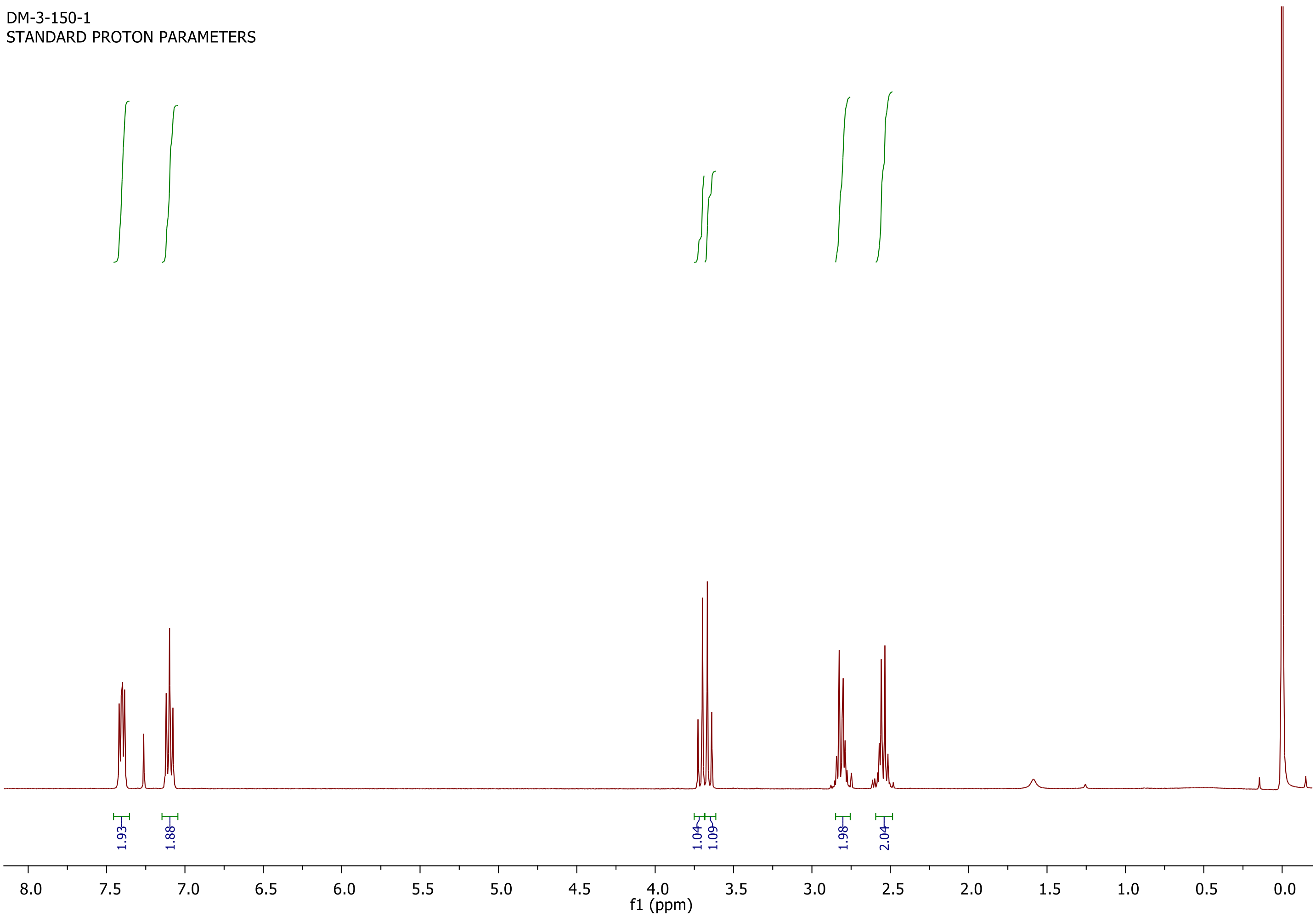

Supplementary figure 32: ^{1}H NMR spectrum of Product **4a**.

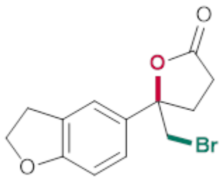

DM-3-60-4
STANDARD PROTON PARAMETERS

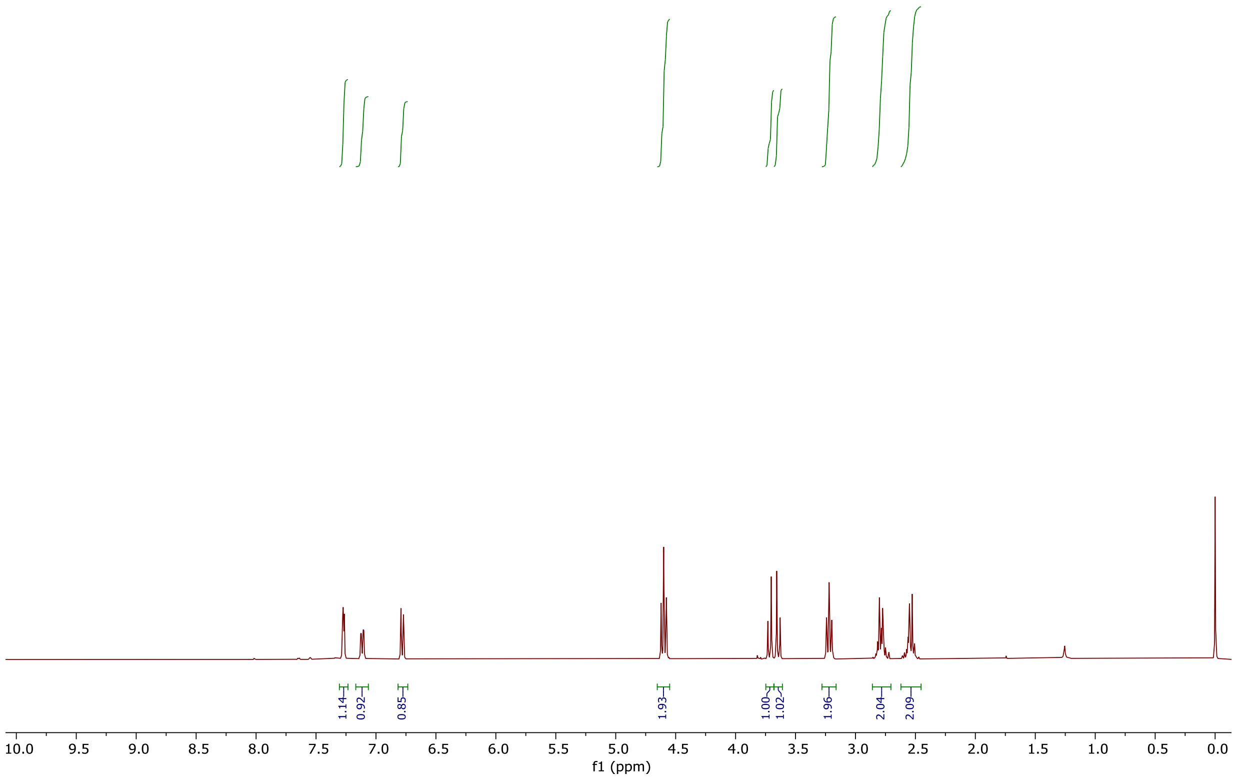

Supplementary figure 33: ^{1}H NMR spectrum of Product **5a**.

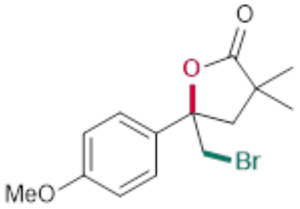

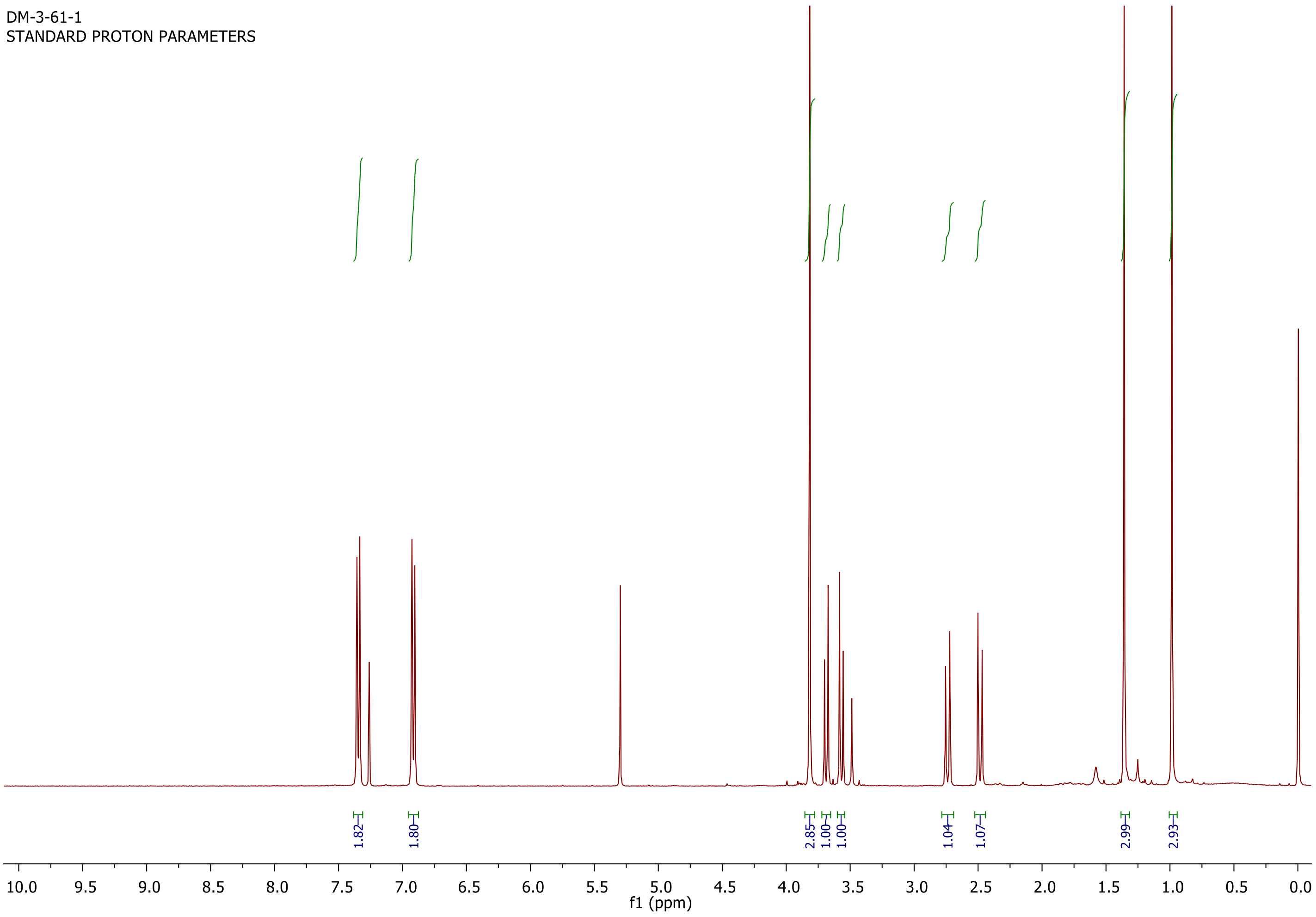

Supplementary figure 34: ^{1}H NMR spectrum of Product **6a**.

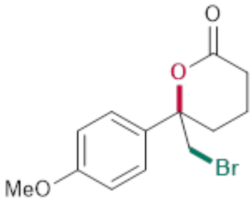

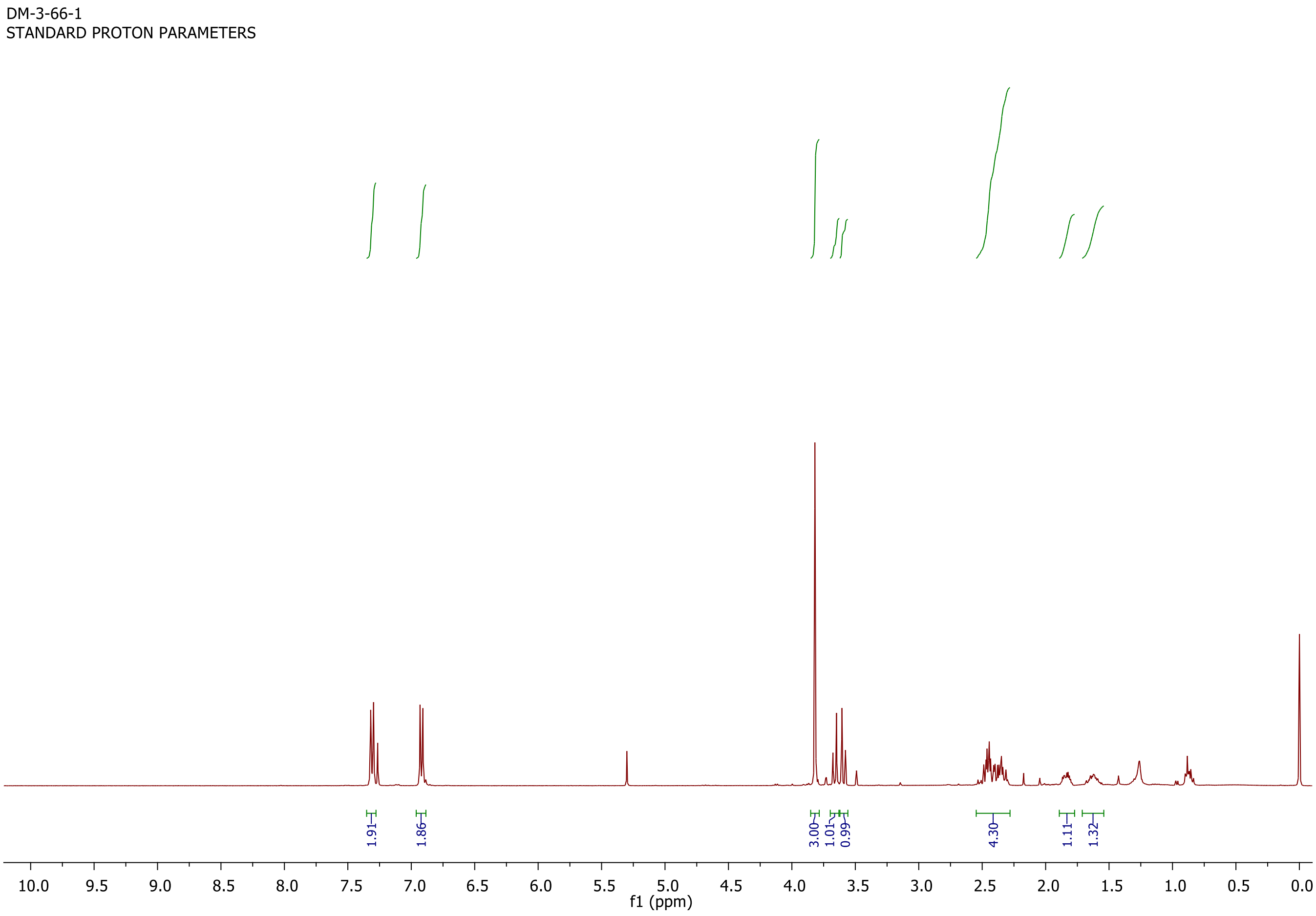

Supplementary figure 35: ^{1}H NMR spectrum of Product **7a**.

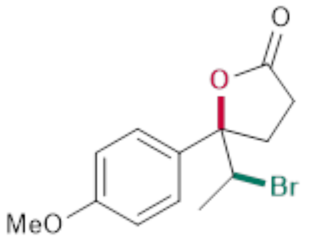

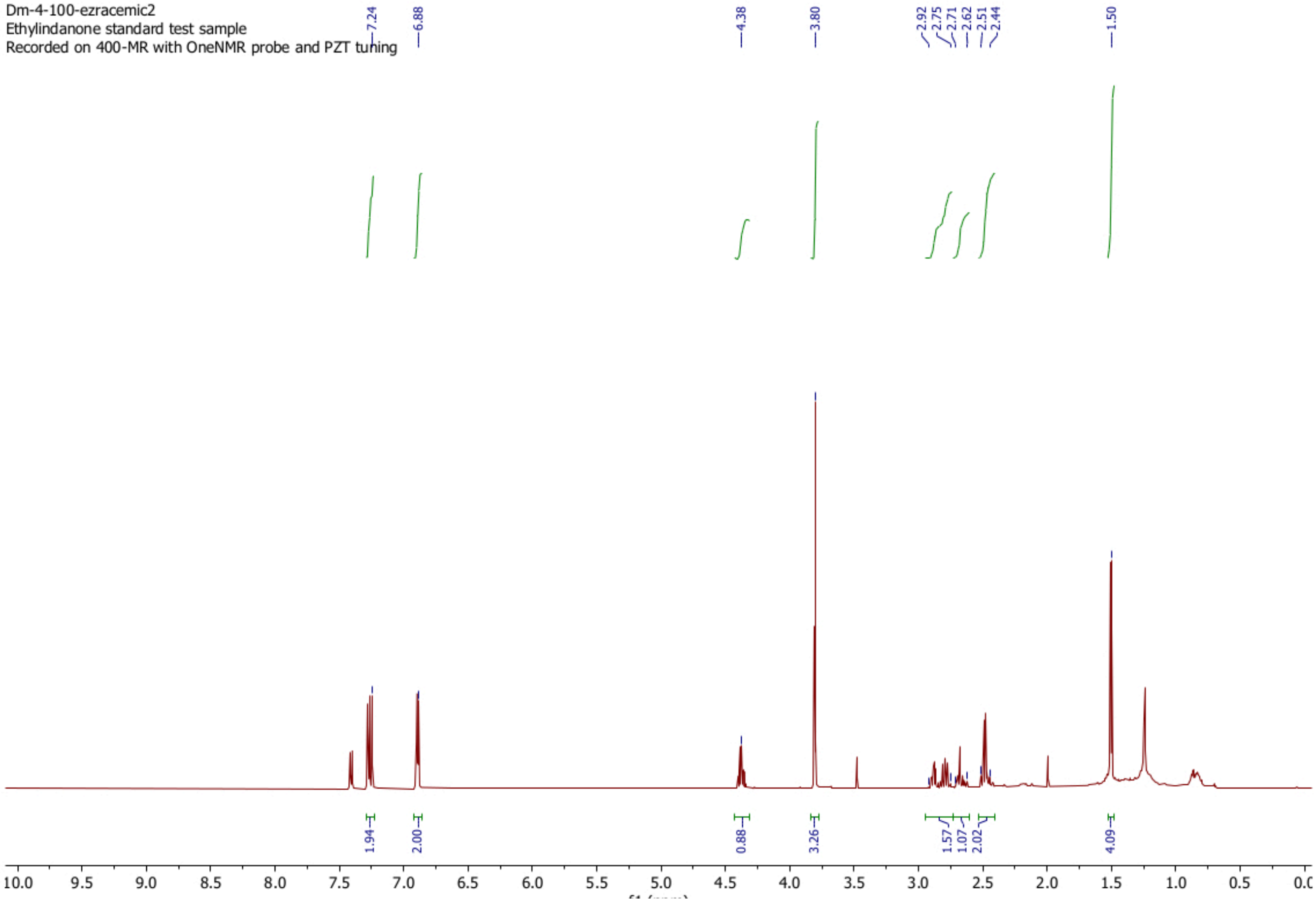

Supplementary figure 36: ^{1}H NMR spectrum of Product **8a**.

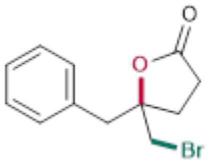

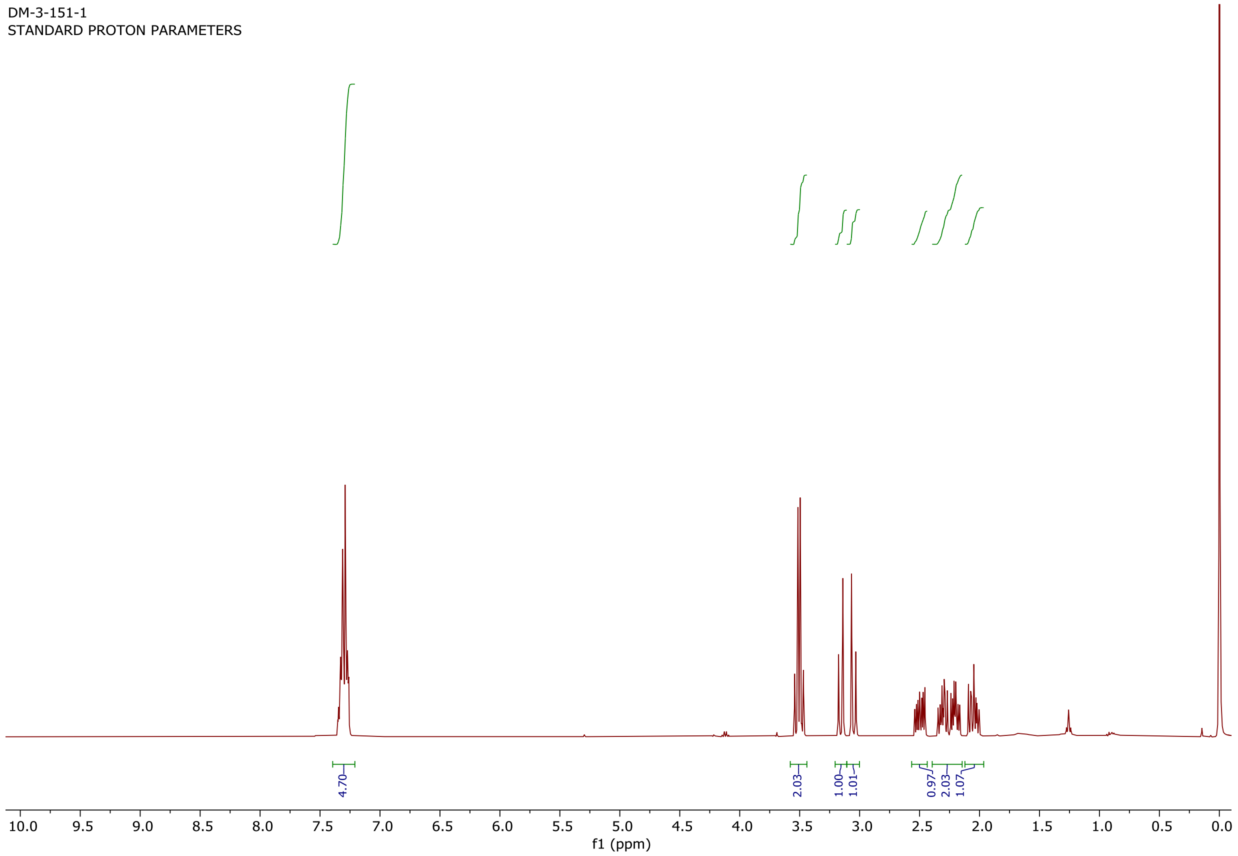

X. Supplementary References

1 Payne, J. T., Andorfer, M. C. & Lewis, J. C. Regioselective Arene Halogenation using the FAD-Dependent Halogenase RebH. *Angew. Chem. Int. Ed.* **52**, 5271-5274 (2013).

2 Ashtekar, K. D., Vetticatt, M., Yousefi, R., Jackson, J. E. & Borhan, B. Nucleophile-Assisted Alkene Activation: Olefins Alone Are Often Incompetent. *J. Am. Chem. Soc.* **138**, 8114-8119 (2016).

3 Jana, S., Verma, A., Rathore, V. & Kumar, S. Synthesis of Novel C2-Symmetric Sulfur-Based Catalysts: Asymmetric Formation of Halo- and Seleno-Functionalized Normal- and Medium-Sized Rings. *Synlett* **30**, 1667-1672, doi:10.1055/s-0037-1610715 (2019).

4 Jefferies, L. R. & Cook, S. P. Iron-catalyzed arene alkylation reactions with unactivated secondary alcohols. *Org. Lett.* **16**, 2026-2029 (2014).

5 Nakatsuji, H., Sawamura, Y., Sakakura, A. & Ishihara, K. Cooperative Activation with Chiral Nucleophilic Catalysts and N-Haloimides: Enantioselective Iodolactonization of 4-Arylmethyl-4-pentenoic Acids. *Angew. Chem.* **126**, 7094-7097 (2014).

6 Yan, Q., Shen, X., Zi, G. & Hou, G. Rh-Catalyzed Asymmetric Hydrogenation of α, β-and β, β-Disubstituted Unsaturated Boronate Esters. *Chemistry–A European Journal* (2020).

7 Murai, K., Nakamura, A., Matsushita, T., Shimura, M. & Fujioka, H. C3-symmetric trisimidazoline-catalyzed enantioselective bromolactonization of internal alkenoic acids. *Chemistry–A European Journal* **18**, 8448-8453 (2012).
